# Loss of SATB2 expression correlates with cytokeratin 7 and PD-L1 tumor cell positivity and aggressiveness in colorectal cancer

**DOI:** 10.1038/s41598-022-22685-0

**Published:** 2022-11-09

**Authors:** Jan Hrudka, Radoslav Matěj, Andrej Nikov, Igor Tomyak, Hana Fišerová, Karolína Jelínková, Petr Waldauf

**Affiliations:** 1 Department of Pathology, 3rd Faculty of Medicine, Charles University, University Hospital Kralovske Vinohrady, Prague, Czech Republic; 2 Department of Pathology and Molecular Medicine, 3rd Faculty of Medicine, Charles University, Thomayer University Hospital, Prague, Czech Republic; 3 Department of Pathology, 1st Faculty of Medicine, Charles University, General University Hospital, Prague, Czech Republic; 4 Department of General Surgery, 3rd Faculty of Medicine, Charles University, University Hospital Kralovske Vinohrady, Prague, Czech Republic; 5 Department of Anaesthesia and Intensive Care Medicine, 3rd Faculty of Medicine, Charles University, University Hospital Kralovske Vinohrady, Prague, Czech Republic

**Keywords:** Gastrointestinal cancer, Oncology, Cancer

## Abstract

Colorectal carcinoma (CRC) is a disease that causes significant morbidity and mortality worldwide. To improve treatment, new biomarkers are needed to allow better patient risk stratification in terms of prognosis. This study aimed to clarify the prognostic significance of colonic-specific transcription factor special AT-rich sequence-binding protein 2 (SATB2), cytoskeletal protein cytokeratin 7 (CK7), and immune checkpoint molecule programmed death-ligand 1 (PD-L1). We analyzed a cohort of 285 patients with surgically treated CRC for quantitative associations among the three markers and five traditional prognostic indicators (i.e., tumor stage, histological grade, variant morphology, laterality, and mismatch-repair/MMR status). The results showed that loss of SATB2 expression had significant negative prognostic implications relative to overall survival (OS) and cancer-specific survival (CSS), significantly shortened 5 years OS and CSS and 10 years CSS in patients with CRC expressing CK7, and borderline insignificantly shortened OS in patients with PD-L1 + CRC. PD-L1 showed a significant negative impact in cases with strong expression (membranous staining in 50–100% of tumor cells). Loss of SATB2 was associated with CK7 expression, advanced tumor stage, mucinous or signet ring cell morphology, high grade, right-sided localization but was borderline insignificant relative to PD-L1 expression. CK7 expression was associated with high grade and SATB2 loss. Additionally, a separate analysis of 248 neoadjuvant therapy-naïve cases was performed with mostly similar results. The loss of SATB2 and CK7 expression were significant negative predictors in the multivariate analysis adjusted for associated parameters and patient age. In summary, loss of SATB2 expression and gain of CK7 and strong PD-L1 expression characterize an aggressive phenotype of CRC.

## Introduction

In 2020, there were 1.93 million cases of colorectal carcinoma (CRC), making it the third most common human malignancy. The associated 935,000 deaths make CRC the second most significant cancer-related cause of death worldwide^1^ and comprise about 10% of human malignant tumors and cancer-related deaths^2^. Localized tumors and the subset of tumors with lymph node metastases can be successfully treated with surgical resection; patients with distant metastases may benefit from (neo)adjuvant treatment. New biomarkers are needed to identify high-risk tumors and stratify patients in terms of prognosis.

Special AT-rich sequence-binding protein 2 (SATB2) is an evolutionarily conserved protein that binds to the matrix attachment regions activating gene transcription in a matrix attachment region-dependent manner, as described in 2003^3,4^. Matrix attachment regions are regulatory DNA sequences important for higher-order chromatin organization and extension of chromatin modifications. Two homologous proteins, SATB1 and SATB2 bind to these DNA sequences with various regulatory functions in gene expression. In mice, SATB2 transcripts have been identified in pre-B cells, the brain, kidneys, thymus, and testis^4^. SATB2 is constitutively expressed in humans and has developmental roles in craniofacial, neural, and osteoblastic differentiation^3^. Haploinsufficiency, caused by the deletion of several genes, has been reported to induce cleft palates and craniofacial malformations^5^. It has been shown that SATB2 is constitutively expressed in colonic mucosa. A recent murine study showed that colonic stem cell identity was lost when SATB2 expression was lost; inversely, the gain of SATB2 expression in small bowel stem cells leads to the conversion into the colonic phenotype^6^. In human pathology, SATB2-immunohistochemistry has gained importance over the last two decades as a marker of osteosarcoma^7,8^. SATB2 is constitutively expressed in the physiological colorectal mucosa and a majority of colorectal adenocarcinomas^9–14^ and is widely used as a routine immunohistochemical marker indicating a colorectal origin to differentiate colorectal adenocarcinomas from other adenocarcinoma primaries. Previous studies described an association between diminished SATB2 expression and a poor CRC prognosis^15–18^.

Cytokeratins (CK7) are cytoskeletal structural proteins present in epithelia and in epithelial tumors. CRC mostly expresses CK20 like normal colon mucosa. CK7 occurs in various glands (breast, skin adnexa, salivary, pancreatobiliary ducts) and in many types of adenocarcinomas (breast, lung, pancreatobiliary, salivary). It is also in a minority of CRC, although the CK20-CK7 + expression profile is often thought to indicate a non-colorectal origin. The rate of CK7 + CRCs varies between 0 and 22% in published studies^19–35^. According to several reports, including our recent study, CK7-expression in CRC is associated with aggressive tumor properties relative to more advanced stages^23,26,36^ and shorter survival times^27,32,34,35^.

The programmed death-ligand 1 (PD-L1) is a cell surface molecule expressed in various immune and tumor cell types. PD-L1 expression allows tumor cells to escape anti-tumor cytotoxic immunity and has been described to worsen the prognosis of several gastrointestinal malignancies, including gastric cancer^36–39^, esophageal cancer^40,41^, and specific subtypes of pancreatic cancer^42,43^. Despite the small subset of CRCs expressing PD-L1, evidence documents its negative impact on the prognosis^44–56^.

To identify particularly aggressive CRC phenotypes, we analyzed a cohort similar to our previous study^57^ with nearly 10 years of follow-up focusing on the association between SATB2, CK7, and PD-L1 expression and correlations with traditional prognostic parameters such as stage, grade, anatomical site, and mismatch-repair (MMR) status along with prognostic implications. As a secondary aim, we analyzed the prognostic impact of PD-L1 expression according to tumor cell expression rate.

## Material and methods

### Study cohort

We selected the medical records from the pathology department, from 2010 to 2013, of 285 patients with surgically resected histopathologically verified adenocarcinoma of the colon and rectum with known follow-ups and formalin-fixed paraffin-embedded (FFPE) resection specimen tumor tissue available. In first step, cases of all stages were included without selection according to neoadjuvant/adjuvant therapy. Additionally, 37 cases which underwent neoadjuvant radio- and/or chemotherapy (prior to surgery) were excluded and a separate analysis of 248 neoadjuvant therapy-naïve cases was performed. No patients received immune-checkpoint inhibitor treatment. Among these cases were conventional adenocarcinoma, mucinous adenocarcinoma, and signet-ring adenocarcinoma. The grade and stage of the tumor were recorded based on medical records. Stages I–IV was assigned using the TNM Classification^58^ and the Union for International Cancer Control (UICC).

### Tissue microarray and immunohistochemistry

Tissue microarray (TMA) techniques were used to make paraffin blocks for immunohistochemical slides using a TMA Master 3D Histech manual tissue arrayer. From each paraffin block containing invasive adenocarcinoma tissue, two cylindrical cores measuring 2 mm were taken from random tumor tissue sites. All cores were collected in a recipient TMA paraffin block. Each recipient block contained 20 samples from 10 cases. For immunohistochemistry, four µm-thick tissue sections were stained in a Ventana BenchMark ULTRA auto-stainer (Ventana Medical Systems, Tucson, Arizona). Monoclonal antibodies against SATB2 (CellMarque, EP281, 1:200), CK7 (clone OV-TL, BioSB, 1:500), PD-L1 (clone SP263, Roche, diagnostic kit), MutS homolog 2 (MSH2, clone G219-1129, Roche, ready to use), postmeiotic segregation 2 (PMS2, clone A16-4, Roche, ready to use), MutS homolog 6 (MSH6, clone 44, Roche, ready to use), and MutL homolog 1 (MLH1, clone M1, Roche, ready to use) were used. The positive reactions were visualized using the Ultraview Detection System (Ventana Medical Systems); slides were counterstained with hematoxylin. Stained slides were dehydrated and covered in a xylene-based mounting medium.

### Microscopical analysis, cut points

**Figure 1:**
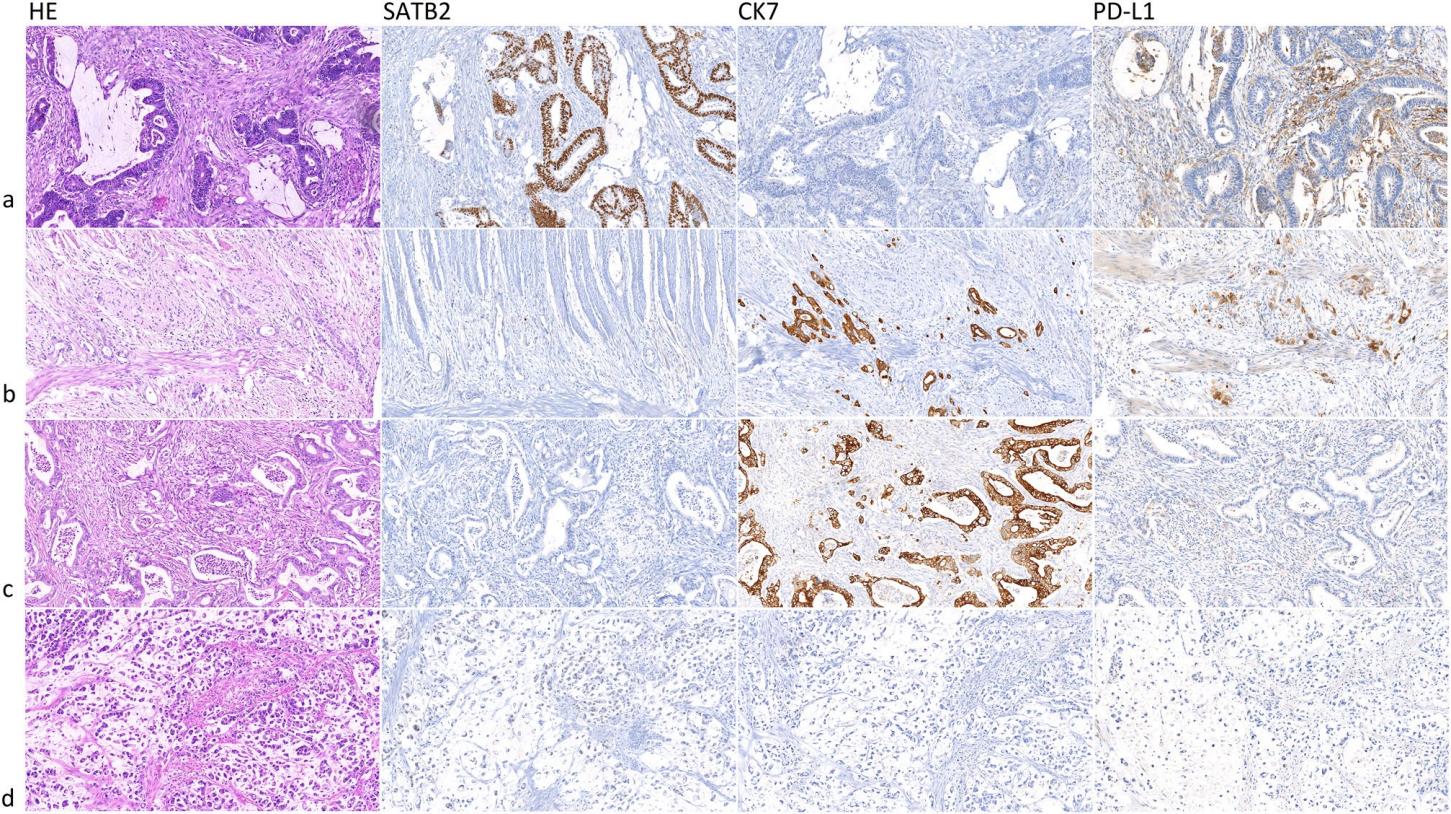
Histological slides showing colorectal carcinoma (CRC), hematoxylin–eosin (HE) stain, and immunohistochemistry, magnification 20×: (**a**) conventional adenocarcinoma with strong nuclear SATB2 positivity, CK7 negativity, and PD-L1 positivity in immune cells and negativity in tumor cells; (**b**) conventional adenocarcinoma with SATB2 negativity (staining in ca 1% tumor cells), strong cytoplasmic CK7 expression in 100% tumor cells, and strong membranous PD-L1 expression in ca 60% tumor cells; (**c**) conventional adenocarcinoma with SATB2 negativity, strong cytoplasmic CK7 positivity in 100% tumor cells, and PD-L1 negativity; (**d**) signet ring cell carcinoma with intracellular and extracellular mucin production showing SATB2 negativity (weak staining in ca 20% tumor cells), CK7 negativity, and PD-L1 negativity.

Using a microscope, two experienced routine pathologists (JH and RM) assessed all immunohistochemical examinations. For SATB2, only nuclear staining was regarded as positive in both weak and strong staining; the percentage of positive tumor cells in both cores was recorded separately. In cases with discordant finding, a consensus value among both cores was stated and recorded using multihead microscope. The cohort was binarized according to an optimal cut-point calculated using optimization of the log-rank test resulting in 40% of SATB2-positive cells being used as the cut-off value. The surv_cut-point function (survminer) determined the optimal cut-point using the maximally selected rank statistics from the `maxstat` R package (see below). According to the cut-point, the cohort was classified into SATB2 high (hi) and SATB2 low (lo). For CK7, the percentage of positive tumor cells in both cores was recorded separately. In case of discordant finding, a consensus value among both cores obtained using multihead microscope was discussed and recorded; staining in ≥ 10% of tumor cells was considered a positive sample. PD-L1 was considered positive only in membranous staining. The percentage of positive neoplastic cells (tumor proportion score—TPS) was recorded following standardized recommendations in both cores separately by both pathologists. In case of a discordant finding, a consensus value was discussed using multihead microscope. In all three examined markers, the consensus value was used in statistical analysis. Regarding MMR status, tumors with any apparent nuclear staining with MSH2, MSH6, PMS2, and MLH1 were considered MMR-proficient. Tumors with an obvious loss of nuclear staining of anti-MMR antibodies with control positivity in stroma and lymphocytes were considered MMR-deficient. All microscopic analyses (Fig. 1) were performed without knowledge of clinical data and patient follow-up.

### Statistics

Overall survival (OS) and cancer-specific survival (CSS) were calculated from the date of surgery to the date of recorded death or the last known follow-up date (censoring). Concerning CSS, patients with non-CRC-related causes of death were censored on the date of death. Separate analyses for 5-year and 10-year follow-ups were performed. For survival analysis, we performed a univariate Kaplan–Meier analysis with the log-rank test and confidence intervals calculated using the log–log method. For estimation of the restricted mean (rmean) survival in each subgroup, a restricted mean survival time (RMST) analysis with a 95% confidence interval (CI) was used. To calculate the hazard ratio (HR) for each parameter, univariate Cox regressions with 95% CIs were performed. Logistic regressions (Pearson's chi-squared test) were used to assess associations between measured variables.

Furthermore, we performed separate 5-year and 10-year OS and CSS analyses on three subgroups, i.e., PD-L1 negative, weak and moderate expressors (CRCs expressing PD-L1 in 1–49% of tumor cells), and strong expressors (50–100%). Survival analysis, RMSE, Cox regression, and logistic regression were performed separately for the binarized cohort as follows: SATB2hi (> 40%) vs. SATB2lo (≤ 40%), CK7+ (≥ 10%) vs. CK7− (< 10%), PD-L1 + (≥ 1%) vs. PD-L1− (< 1%), low grade (grade 1 + 2) vs. high grade (grade 3), conventional adenocarcinoma vs. adenocarcinoma with variant morphology (mucinous + signet ring carcinoma), MMR-proficient vs. MMR-deficient, right sided tumors (cecum, ascendens, hepatic flexure, transversum) versus left sided tumors (lienal flexure, descendens, sigmoid, rectum), and localized tumors (UICC stages 1 + 2) vs. advanced tumors (UICC stages 3 + 4).

In the next step, multivariate Cox regressions adjusting associated parameters and adjusting analyzed variables on the patient's age were performed. To evaluate reliability rates among two tumor tissue cores and among both pathologists, unweighted Cohen`s Kappa value was calculated. The Cohen´s kappa test was used to analyze binarized variables using cut-off values as described above (SATB2hi vs. SATB2lo, CK7+ vs. CK7−, PD-L1+ (≥ 1%) vs. PD-L1− (< 1%)). *P* values < 0.05 were considered statistically significant. All analyses were performed in R version 4.0.3 (2020-10-10)^59^; survival analyses were done using package survival version 3.2-7^60^.

### Ethics

The study was approved by the University Hospital Královské Vinohrady ethics committee, approval number EK-R/04/012022. The University Hospital Královské Vinohrady waived the need for informed consent due to the study's retrospective nature. The research was performed following the Declaration of Helsinki.

## Results

### Cohort description

**Figure 2:**
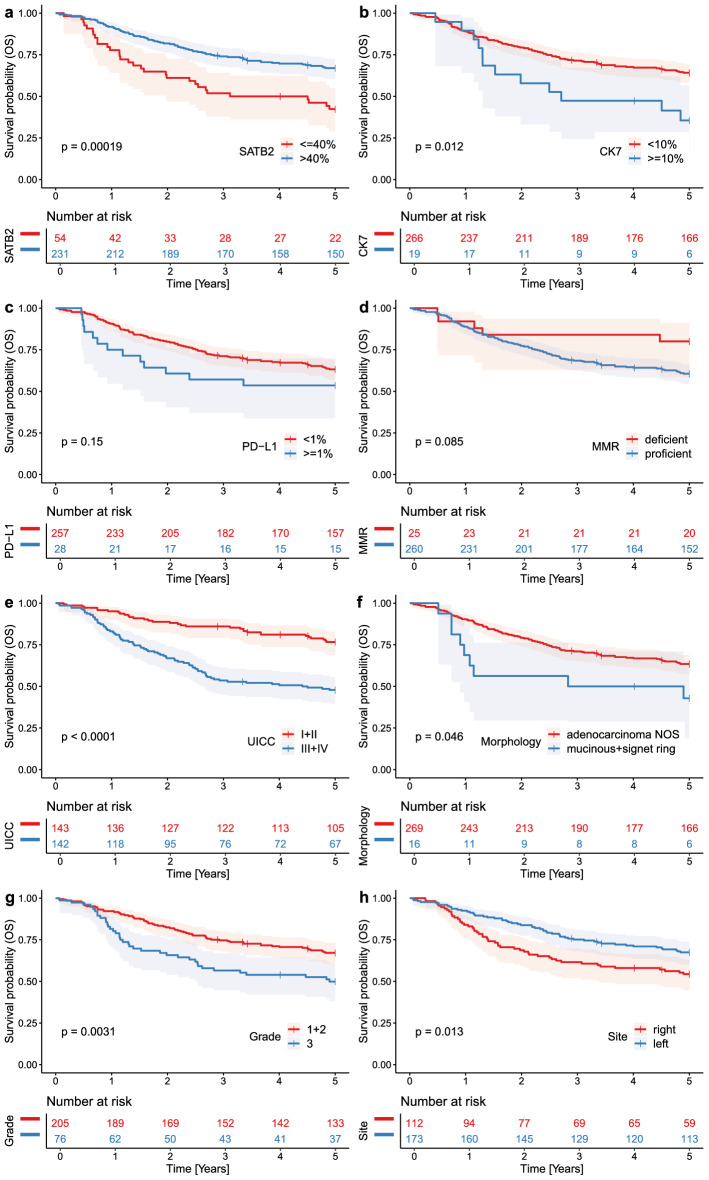
Entire cohort, Kaplan Meier curves documenting 5-year overall survival (OS) in patients with CRC. Note decreased survival in patients with CRC with SATB2 low expression (**a**), CK7 high expression (**b**), PD-L1 expression (**c**), mismatch-repair proficient status (**d**), advanced stage (**e**), mucinous or signet ring cell morphology (**f**), high-grade tumors (**g**), and right-sided tumor localization (**h**). P values from the log-rank test < 0.05 indicate a significant difference in survival.

**Figure 3:**
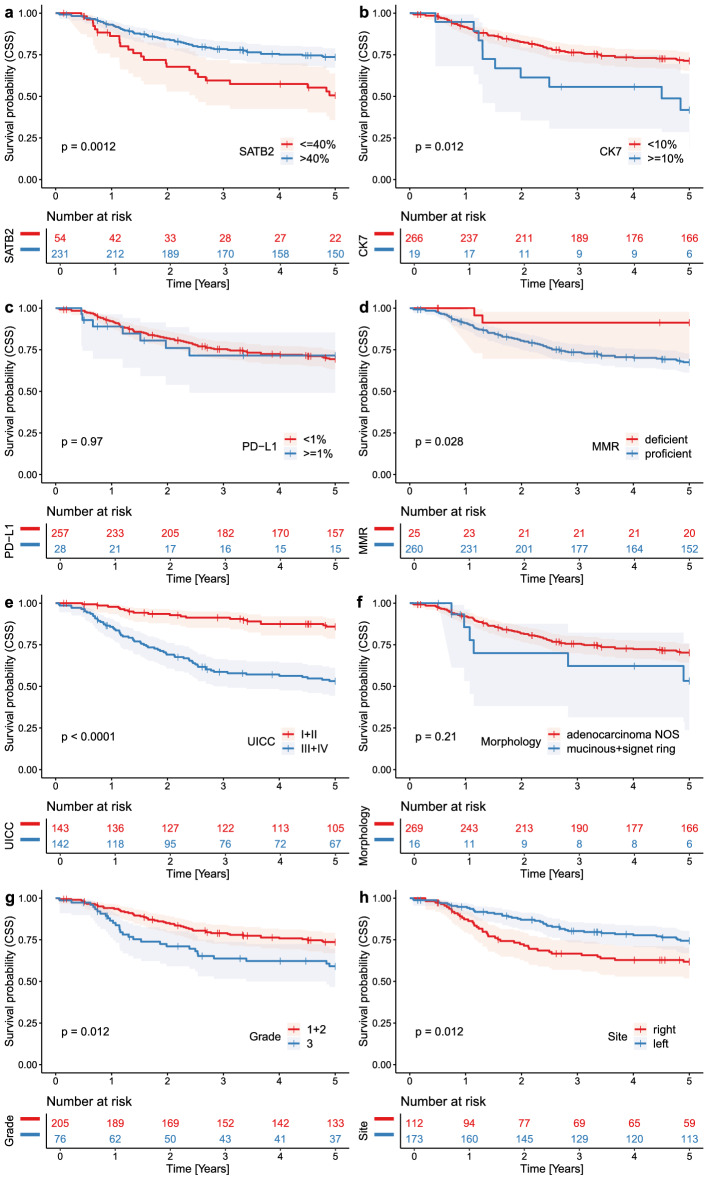
Entire cohort, Kaplan Meier curves documenting worse 5-year cancer-specific survival (CSS) in patients with CRC with SATB2 low expression (**a**), CK7 high expression (**b**), mismatch-repair proficient status (**d**), advanced stage (**e**), high-grade tumors (**g**), and right-sided tumor localization (**h**). There is little and no impact of a mucinous or signet ring morphology (**f**), and PD-L1 expression (**c**), respectively. P values from the log-rank test < 0.05 indicate a significant difference in survival.

The entire cohort consisted of 161 male and 124 female patients, with a mean age of 68.55 years, a median age of 69 years, a mode of 70 years, and a range of 30–94 years. The neoadjuvant therapy-naïve cohort consisted of 136 male and 112 female patients, with a mean age of 69.2 years, and a range of 30–94 years. 37 cases which underwent neoadjuvant radio- and/or chemotherapy had a tumor situated in rectum or rectosigmoid. All variables analyzed in the study are summarized in Table 1 (entire cohort) and Table 2 (neoadjuvant therapy-naïve cohort) and Supplementary Table 1. Results of the 5-year OS and CSS analyses of the entire cohort are summarized in Supplementary Table 2, and the 10-year OS and CSS analyses of the entire in Supplementary Table 3. The results of 5- and 10-year survival analysis of the neoadjuvant therapy-naïve cohort are summarized in Supplementary Tables 4 and 5, respectively. Kaplan–Meier curves showing the 5-year OS and CSS in the entire cohort according to immunohistochemical profile and traditional variables are shown in Figs. 2 and 3, respectively. Kaplan–Meier curves showing the 10-year OS and CSS in the entire cohort are shown in Supplementary Figs. 1 and 2, respectively. Kaplan–Meier curves showing survival in the neoadjuvant therapy-naïve cohort are shown in Supplementary Figs. 3–6.

**Table 1:** Entire cohort. Summary of all cases and all examined variables, and the number of subjects according to their CK7/SATB2/PD-L1 profile.

	CK7− SATB2lo PD-L1negative	CK7+ SATB2lo PD-L1negative	CK7− SATB2hi PD-L1 negative	CK7+ SATB2hi PD-L1negative	CK7− SATB2lo PD-L1 ≥ 1%	CK7+ SATB2lo PD-L1 ≥ 1%	CK7− SATB2hi PD-L1 ≥ 1%	CK7+ SATB2hi PD-L1 ≥ 1%	Total
Total	34	11	206	6	7	2	19	0	285 (100%)
**Age group**									
< 50	1	1	10	0	0	0	1	0	13 (4.6%)
50–59	4	3	29	2	0	0	2	0	40 (14.0%)
60–69	10	4	74	1	0	0	5	0	94 (33.0%)
70–79	12	1	56	2	5	2	7	0	85 (29.8%)
80+	7	2	37	1	2	0	4	0	53 (18.6%)
**Sex**									
F	11	8	88	1	4	1	11	0	124 (43.5%)
M	23	3	118	5	3	1	8	0	161 (56.5%)
**UICC stage**									
I	3	1	22	2	0	0	5	0	33 (11.6%)
II	10	4	82	3	1	0	10	0	110 (38.6%)
III	14	5	77	1	5	0	4	0	106 (37.2%)
IV	7	1	25	0	1	2	0	0	36 (12.6%)
**Situs**									
Cecum	6	4	23	0	1	1	3	0	38 (13.3%)
Ascendens	4	3	23	1	1	0	2	0	34 (11.9%)
Hepatic flexure	3	0	9	0	1	0	1	0	14 (4.9%)
Transversum	2	0	10	0	0	0	2	0	14 (4.9%)
Lienal flexure	1	0	8	1	0	0	0	0	10 (3.5%)
Descendens	0	1	9	1	0	0	1	0	12 (4.2%)
Sigmoideum	8	1	49	1	0	0	7	0	66 (23.2%)
Rectosigmoideum	2	0	18	0	0	0	3	0	23 (8.1%)
Rectum	8	2	55	2	3	1	0	0	71 (24.9%)
Multiple	0	0	20	0	1	0	0	0	3 (1.1%)
**MMR**									
Deficient	4	1	13	1	2	0	4	0	25 (8.8%)
Proficient	30	10	193	5	5	2	15	0	260 (91.2%)
**Grade**									
Low grade (1 + 2)	23	5	158	3	3	1	12	0	205 (73.0%)
High grade (3)	11	6	46	3	2	1	7	0	76 (27.0%)
Unknown	0	0	2	0	2	0	0	0	4
**Morphology**									
Adenocarcinoma NOS	30	9	198	6	6	2	18	0	269 (94.4%)
Mucinous + signet ring	4	2	8	0	1	0	1	0	16 (5.6%)

**Table 2:** Neoadjuvant therapy-naïve cases and all examined variables, and the number of subjects according to their CK7/SATB2/PD-L1 profile.

	CK7− SATB2lo PD-L1negative	CK7+ SATB2lo PD-L1negative	CK7− SATB2hi PD-L1 negative	CK7+ SATB2hi PD-L1negative	CK7− SATB2lo PD-L1 ≥ 1%	CK7+ SATB2lo PD-L1 ≥ 1%	CK7− SATB2hi PD-L1 ≥ 1%	CK7+ SATB2hi PD-L1 ≥ 1%	Total
Total	31	11	175	5	5	2	19	0	248 (100%)
**Age group**									
< 50	1	1	10	0	0	0	1	0	13 (5.2%)
50–59	4	3	25	1	0	0	2	0	35 (14.1%)
60–69	7	4	57	1	0	0	5	0	74 (29.8%)
70–79	12	1	46	2	3	2	7	0	73 (29.4%)
80+	7	2	37	1	2	0	4	0	53 (21.4%)
**Sex**									
F	11	8	77	1	3	1	11	0	112 (45.2%)
M	20	3	98	4	2	1	8	0	136 (54.8%)
**UICC stage**									
I	1	1	18	2	0	0	5	0	27 (10.9%)
II	10	4	69	2	1	0	10	0	96 (38.7%)
III	13	5	63	1	4	0	4	0	90 (36.3%)
IV	7	1	25	0	0	2	0	0	35 (14.1%)
**Situs**									
Cecum	6	4	23	0	1	1	3	0	38 (15.3%)
Ascendens	4	3	23	1	1	0	2	0	34 (13.7%)
Hepatic flexure	3	0	9	0	1	0	1	0	14 (5.6%)
Transversum	2	0	10	0	0	0	2	0	14 (5.6%)
Lienal flexure	1	0	8	1	0	0	0	0	10 (4.0%)
Descendens	0	1	9	1	0	0	1	0	12 (4.8%)
Sigmoideum	8	1	48	1	0	0	7	0	65 (26.2%)
Rectosigmoideum	2	0	16	0	0	0	3	0	21 (8.5%)
Rectum	5	2	27	1	1	1	0	0	37 (14.9%)
Multiple	0	0	2	0	1	0	0	0	3 (1.2%)
**MMR**									
Deficient	4	1	13	1	2	0	4	0	25 (10.1%)
Proficient	27	10	162	4	3	2	15	0	223 (89.9%)
**Grade**									
Low grade (1 + 2)	20	5	130	3	3	1	12	0	174 (74.7%)
High grade (3)	9	6	36	2	1	1	7	0	59 (25.3%)
Unknown	0	0	2	0	1	0	0	0	4
**Morphology**									
Adenocarcinoma NOS	27	9	168	5	4	2	18	0	233 (94.0%)
Mucinous + signet ring	4	2	7	0	1	0	1	0	15 (6.0%)

The unweighted Cohen’s kappa test showed almost perfect intercore reliability in SATB2 (К = 0.85) and CK7 (К = 0.913); in PD-L1, there was a substantial agreement among both pathologists in core 1 (К = 0.795), and nearly perfect agreement among both pathologists in core 2 (К = 0.884). All findings in individual cores are listed in Supplementary Table 1, the kappa values are listed in Supplementary Table 6.

### Entire cohort

#### SATB2 survival analysis

The patients with SATB2lo tumors (n = 54) had significantly shorter 5-year OS (rmean = 3.157 vs. 4.008 years, HR = 2.17, p = 0.00019, Fig. 2a) and 5-year CSS (rmean = 3.494 versus 4.171 years, HR = 2.16, p = 0.0012, Fig. 3a) compared to those with SATB2hi tumors (n = 231). Analyzing the 10-year follow-up, there was significantly shorter OS (restricted mean/rmean = 4.921 years vs. 6.943 years, hazard ratio = 1.96, p = 0.00042, Sup. Fig. 1a) and CSS (rmean = 5.633 years versus 7.648 years, HR = 2.21, p = 0.00027, Sup. Fig. 2a) in the patients with SATB2lo tumors.

#### CK7 survival analysis

In the 5-year follow-up, there was a negative prognostic impact associated with CK7 expression with significantly shorter OS (rmean = 3.124 vs. 3.898, HR = 2.13, p = 0.012, Fig. 2b) and CSS (rmean = 3.378 vs. 4.096 years, HR = 2.28, p = 0.012, Fig. 3a) comparing CK7 + (n = 19) with CK7 − (n = 266) cases, respectively. In the 10-year follow-up, CK7 expression had a detrimental prognostic impact with borderline insignificantly shorter OS (rmean 4.9 years vs. 6.677 years, HR = 1.70, p = 0.077, Sup. Fig. 1b) and significantly shorter CSS (rmean = 5.468 years vs. 7.412, HR = 2.0, p = 0.035, Sup. Fig. 2b).

#### PD-L1 survival analysis

In the 5-year follow up, PD-L1 + tumors (n = 28) displayed insignificantly shorter OS (rmean = 3.261 vs. 3.91 years, HR = 1.52, p = 0.156, Fig. 2c) and no differences in CSS (rmean = 3.948 vs. 4.063, HR-1.02, p = 0.97, Fig. 3c) compared to PD-L1- (n = 257) cases. In the 10-year follow up, PD-L1 expression showed insignificantly shorter OS (rmean = 5.527 years vs. 6.670 years, HR = 1.46, p = 0.15, Sup. Fig. 1c) and no significant difference in CSS (rmean = 7.29 years vs. 7.329 years, HR = 0.98, p = 0.97, Sup. Fig. 2c) between PD-L1+ and PD-L1− CRCs.

**Figure 4:**
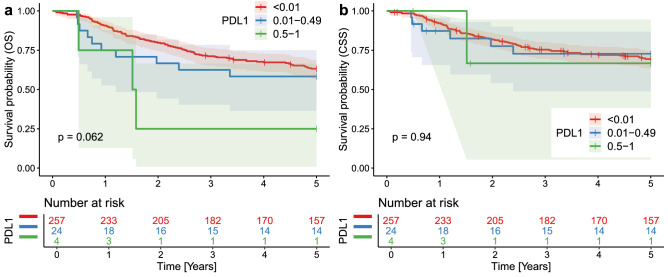
Entire cohort, Kaplan Meier curves documenting (**a**) worse 5-year overall survival (OS) in patients with CRC strongly (50–100% tumor cells) expressing PD-L1 compared to weak and moderate expressors and negative cases; (**b**) no significant differences in cancer-specific survival (CSS). P values from the log-rank test < 0.05 indicate a significant difference in survival.

PD-L1 was further analyzed by categorizing the patients based on the percentage of tumor cells expressing PD-L1 with the following results (Supplementary Table 7): strong PD-L1 expressors (expression in 50–100% of tumor cells, n = 4) compared to weak and moderate (1–49%, n = 24) and negative cases (0% n = 257) displayed borderline insignificantly shorter 5-year OS (rmean = 3.910 vs. 3.446 vs. 2.148 years, respectively, p = 0.062, Fig. 4a) and significantly shorter 10-year OS (rmean = 2.641 vs. 6.033 vs. 6.670 years, respectively, p = 0.01). There were no significant differences in both 5-year CSS (rmean = 3.838 vs. 3.976 vs. 4.063 years, p = 0.94, Fig. 4b) and in 10-year CSS (rmean = 7.171 vs. 7.392 vs. 7.29 years, p = 0.96).

#### Traditional prognosticators

Among traditional prognostic variables studied in the 5-year survival analysis, OS was significantly shorter in case of advanced UICC stage (p < 0.0001, Fig. 2e), grade 3 (p = 0.0031, Fig. 2g), right-sided tumors (p = 0.012, Fig. 2h), and carcinomas with mucinous or signet ring cell morphology (p = 0.046, Fig. 2f), whereas CSS was worse in tumors with advanced UICC stages (p < 0.0001, Fig. 3e), grade 3 (p = 0.012, Fig. 3g), right-sided tumors (p = 0.012, Fig. 3h), and MMR-proficient CRCs (p = 0.028, Fig. 3d)—see Supplementary Table 2. In the 10-year OS, there was a significant negative prognostic impact of advanced UICC stages (p < 0.0001, Sup. Fig. 1e) and grade 3 (p = 0.013, Sup. Fig. 1g). Concerning the 10-year follow-up for CSS, there was a significant worsening effect of the MMR-proficient status (p = 0.0091, Sup. Fig. 2d), advanced UICC stage (p < 0.0001, Sup. Fig. 2e), grade 3 (p = 0.021, Sup. Fig. 2g), and borderline insignificant effect of right-sided tumor sites (p = 0.064, Sup. Fig. 2h)—see Supplementary Table 3.

#### Logistic regression

**Figure 5:**
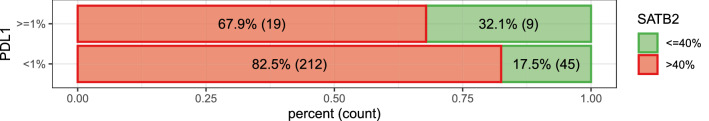
Bar chart documenting borderline insignificant enrichment of SATB2 negative CRCs with PD-L1 expression in ≥ 1% tumor cells (logistic regression, p = 0.066).

The results of the logistic regression analyses (Pearson's chi-squared test) are summarized in Table 3. The analyses revealed significant associations: SATB2lo tumors were prone to be in a more advanced stage (Odds ratio = 0.468, p = 0.016), high-grade (OR = 0.518, p = 0.042), with variant mucinous or signet-ring cell morphology (OR = 3.674, p = 0.014), right-sided (OR = 2.068, p = 0.017), and cytokeratin 7 positive (OR = 0.084, p < 0.001). Furthermore, there was a borderline insignificant association of SATB2 negativity with PD-L1 expression in CRC (OR = 0.448, p = 0.066, Fig. 5). CK7 positive tumors were more frequently high-grade (OR = 3.3, p = 0.013) and borderline insignificantly more right-sided (OR = 0.445, p = 0.093). No association of CK7 expression with UICC stage, morphology, PD-L1 status, or MMR status was found. PD-L1+ CRCs were more frequently MMR-deficient compared to PD-L1− tumors (OR = 0.293, p = 0.018). No association of PD-L1 positivity with UICC stage, grade, morphology, laterality, and CK7 status was identified. A significant association between CK7 expression and SATB2 low status and the borderline insignificant association of PD-L1 expression with SATB2 low status were described above.

**Table 3:**
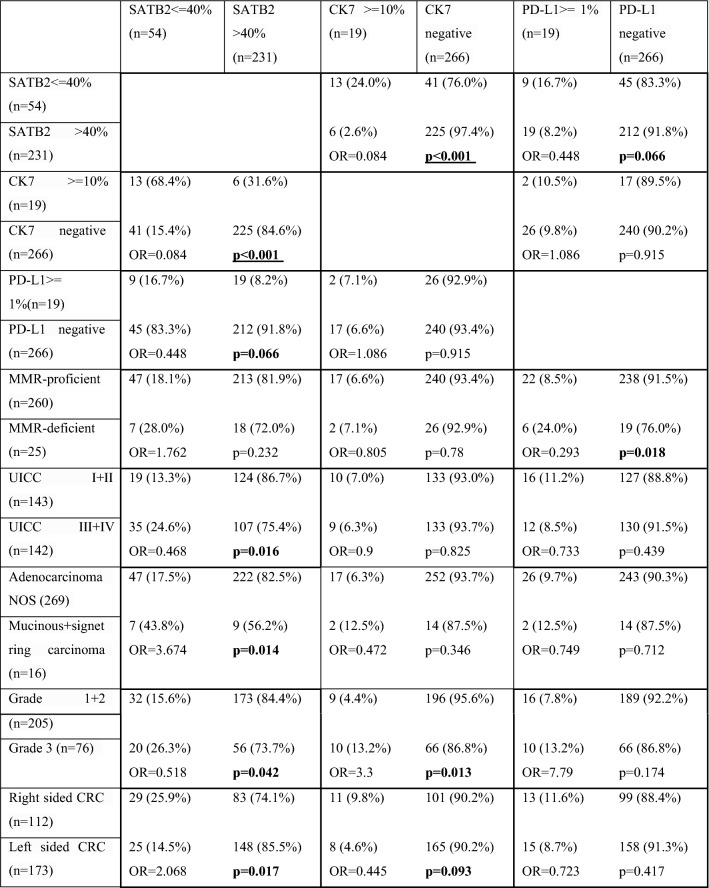
Results of logistic regressi﻿on, significant and borderline insignificant associations are in bold, the main findings of the study are in bold and underlined—association of CK7 expression and SATB2 loss.

### Neoadjuvant therapy-naïve cohort

#### SATB2 survival analysis

After exclusion of patients after neoadjuvant therapy, SATB2lo status (n = 49) remained a significant predictor of shorter 5-year OS (rmean = 3.022 vs. 3.961 years, HR = 2.28, p = 0.00011, Sup. Fig. 3a), 5-year CSS (rmean = 3.383 versus 4.133 years, HR = 2.26, p < 0.001, Sup. Fig. 4a), 10-year OS (rmean = 4.621 years vs. 6.811 years, hazard ratio = 2.04, p = 0.00029, Sup. Fig. 5a), and 10-year CSS (rmean = 5.376 years versus 7.574 years, HR = 2.32, p = 0.00022, Sup. Fig. 6a) compared to those with SATB2hi tumors (n = 199).

#### CK7 survival analysis

After elimination of pretreated patients, CK7 expression retained it’s negative prognostic impact in 5-year OS (rmean = 3.019 vs. 3.835, HR = 2.16, p = 0.01, Sup. Fig. 3b), 5-year CSS (rmean = 3.281 vs. 4.048 years, HR = 2.35, p = 0.0098, Sup. Fig. 4b), and 10-year CSS (rmean = 5.178 years vs. 7.317, HR = 2.12, p = 0.023, Sup. Fig. 5b) in CK7+ (n = 18) versus CK7− (n = 230) tumors, respectively. In 10-year OS, the effect was borderline insignificant (rmean 4.606 years vs. 6.515 years, HR = 1.75, p = 0.062, Sup. Fig. 6b), similarly to the entire cohort.

#### PD-L1 survival analysis

In the neoadjuvant therapy-naïve patients, PD-L1 expression showed insignificantly shorter 5-year OS (rmean = 3.227 vs. 3.84 years, HR = 1.42, p = 0.26, Sup. Fig. 3c) and 10-year OS (rmean = 5.616 years vs. 6.468 years, HR = 1.29, p = 0.37, Sup. Fig. 5c) and no significant difference in both 5-year CSS (rmean = 3.982 vs. 4.0, HR = 0.88, p = 0.77, Sup. Fig. 4c) and 10-year CSS (rmean = 7.678 years vs. 7.133 years, HR = 0.76, p = 0.51, Sup. Fig. 6c) comparing PD-L1+ (n = 26) and PD-L1− (n = 222) tumors, respectively.

PD-L1 was analyzed according to the percentage of tumor cells expressing PD-L1 (Supplementary Table 8). Similarly, to the entire cohort, the patients with strong PD-L1 expression (50–100%, n = 4) compared to both weak and moderate (1–49%, n = 22) and negative tumors (0%, n = 222) displayed significantly shorter 10-year OS (rmean = 6.468 vs. 6.19 vs. 2.641 years, respectively, p = 0.019) and borderline insignificantly shorter 5-year OS (rmean = 6.873 vs. 6.378 vs. 3.398, respectively, p = 0.099). There were almost no differences in both 10-year CSS (rmean = 7.133 vs. 7.781 vs. 7.171, p = 0.74) and 5-year CSS (rmean = 7.367 vs. 7.781 vs. 7.171, p = 0.89).

#### Traditional prognosticators

After exclusion of pretreated cases, 5-year OS was significantly shorter along with advanced UICC stage (p < 0.0001, Sup. Fig. 3e), and grade 3 (p = 0.0033, Sup. Fig. 3g), and borderline insignificantly shorter in right-sided tumors (p = 0.057, Sup. Fig. 3h), in tumors with mucinous and signet ring cell morphology (p = 0.17, Sup. Fig. 3f), and in MMR-proficient CRCs (p = 0.053, Sup. Fig. 3d). 5-year CSS was shorter in tumors with advanced UICC stages (p < 0.0001, Sup. Fig. 4e), grade 3 (p = 0.038, Sup. Fig. 4g), right-sided tumors (p = 0.036, Sup. Fig. 4h), and MMR-proficient CRCs (p = 0.019, Sup. Fig. 4d)—see Supplementary Table 4. In the 10-year OS, there was a significant negative prognostic effect of advanced UICC stages (p < 0.0001, Sup. Fig. 5e) and borderline insignificant impact of MMR-proficient status (p = 0.094, Sup. Fig. 5d), and grade 3 (p = 0.12, Sup. Fig. 5g). In the 10-year CSS, there was significantly worse survival in the MMR-proficient tumors (p = 0.0072, Sup. Fig. 6d), CRCs in advanced UICC stage (p < 0.0001, Sup. Fig. 6e), and borderline insignificantly worse survival in grade 3 tumors (p = 0.087, Sup. Fig. 6g) and right-sided tumors (p = 0.1, Sup. Fig. 6h)—see Supplementary Table 5.

#### Logistic regression

**Figure 6:**
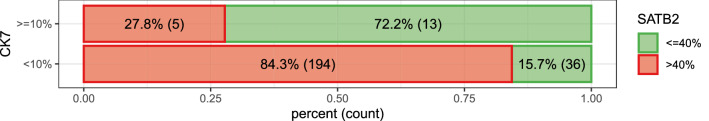
Bar chart documenting significant enrichment of SATB2 negative CRCs with CK7 expression in the neoadjuvant therapy-naïve cohort (logistic regression, p < 0.001).

The results of the logistic regression analyses (Pearson's chi-squared test) after exclusion of cases after neoadjuvant therapy are summarized in Supplementary Table 9. The analyses showed following significant associations: SATB2lo tumors were more frequently in advanced stage (OR = 0.466, p = 0.022), with variant mucinous or signet-ring cell morphology (OR = 5.979, p = 0.011), right-sided (OR = 2.027, p = 0.029), and cytokeratin 7 positive (OR = 0.071, p < 0.001, Fig. 6). CK7 positive tumors were more frequently high-grade, the association was borderline insignificant (OR = 2.486, p = 0.087). PD-L1+ CRCs were again more frequently MMR-deficient compared to PD-L1− tumors (OR = 0.312, p = 0.026).

#### Multivariate Cox regression

In the next step, the prognostic impact of SATB2 and CK7 on the 5-year CSS was analyzed using a multivariate Cox regression with 95% CIs adjusting for associated variables in the neoadjuvant therapy-naïve cohort. The associations were known from previous logistic regression. The 5-year survival was analyzed using a multivariate analysis relative to the higher level of statistical significance compared to the 10-year survival. In this multivariate analysis, SATB2 low status remained a significant predictor (HR = 1.92, p = 0.011) of poor 5-year CSS if adjusted on patient´s age (HR = 1.023, p = 0.032), UICC stage (HR = 5.49, p < 0.001), mucinous or signet ring cell morphology (HR = 1.156, p = 0.759) but SATB2 was insignificant (HR = 1.56, p = 0.131) if adjusted on CK7 positivity (HR = 2.08, p = 0.061). CK7 remained a significant (HR = 2.12, p = 0.038) negative prognostic factor if adjusted on patient´s age (HR = 1.02, p = 0.111) and histopathological grade (HR = 1.6, p = 0.07) but CK7 was insignificant (HR = 1.56, p = 0.262) if adjusted on SATB2 (HR = 1.77, p = 0.05).

#### Summary of results

In summary, our study revealed significantly shorter OS and CSS in patients with SATB2lo and CK7+ CRC and borderline insignificantly shorter OS in patients with PD-L1+ CRC in the neoadjuvant therapy-naïve and in the entire cohort. SATB2 was significant stage- and age-independent negative predictor in the multivariate Cox regression. SATB2 low status was associated with CK7 expression, right-sided site, mucinous or signet ring cell morphology, and advanced UICC stages. CK7 expression was significant predictor of dismal prognosis independently from patient´s age and tumor grade in the multivariate analysis. Both strongly associated SATB2 low status and CK7 expression were insignificant predictors in the multivariate analysis if adjusted on CK7 and SATB2, respectively. The association of SATB2 loss with PD-L1 expression was borderline insignificant (p = 0.066) in the entire cohort and insignificant after exclusion of pretreated patients. PD-L1 expression was a borderline insignificant negative OS predictor without predictive value for CSS; PD-L1 expression was associated with MMR-deficient status; strong PD-L1 expression (> 49% of tumor cells) was a significant poor OS predictor but with no impact on CSS in both analyses.

## Discussion

SATB2 is widely used by pathologists as a sensitive and specific marker of a colorectal origin in adenocarcinomas. As expressed in the majority of CRCs, loss of SATB2 has been identified as a negative prognostic marker in several studies. Wang et al. described an association between SATB2 low expression and the presence of lymph node and distant metastases, advanced Dukes’ stage, shorter overall, and disease-free survival in CRC^15^. Mezheyeuski et al. linked strong SATB2 expression to left-sided tumor localization, low-grade, non-mutated BRAF status, longer overall survival, and better responsiveness to chemotherapy^17^. Schmitt et al. recently compared the negative prognostic roles of SATB2 loss and caudal type homeobox transcription factor 2 (CDX2) loss; the authors found a negative prognostic relevance for SATB2 loss in both univariate and multivariate analyses and confirmed its prognostic superiority compared to the loss of CDX2^18^. Eberhart et al. described SATB2 expression as an independent favorable prognostic marker in CRC and as a predictor of response to adjuvant and neoadjuvant chemotherapy^16^. Relative to SATB2 being a response to chemotherapy predictor, the favorable value of its expression in CRC^16^ sharply contrasts with its negative therapy-predictive value in different cancers, e.g., head and neck squamous carcinoma^61^. This contradiction illustrates the complex role of SATB2 in cancer biology, where it has been primarily described as a transcription factor in craniofacial embryogenesis^62^. In CRC, SATB2 should be regarded as a differentiation marker and, thus, as a beneficial feature of a low-risk phenotype since it may act as an oncogene in non-colonic cell populations. SATB1 is a member of the same protein family that has been described as a cancer promoter in breast tumors and has been linked to poor prognosis^63^. Opposite of SATB2, SATB1 has been linked to rectal cancer progression^64^ but without survival analysis.

Eberhart et al. documented a significant association between SATB2 expression and microsatellite stability^16^. Our data suggest a slight enrichment of MMR-deficient status in SATB2 low tumors, although the difference did not reach statistical significance. Similarly, the loss of widely known colorectal differentiation markers CK20 and CDX2 has been described to be more frequent in MMR-deficient tumors^24^. This may be explained by the genomic instability and high mutation loads in MSI CRC, which lead to the loss of intestinal markers.

Our data are in line with well-established research that led to the identification of SATB2 loss as a robust marker of a poor prognosis in CRC. We found significantly shorter OS and CSS, an inclination toward advanced UICC stage, right-sided localization, and variant (mucinous or signet ring cell) morphology. The prognostic value of SATB2 loss was an age- and stage-independent negative predictor in the multivariate analysis. As a novel finding, we identified a strong association between SATB2 loss with CK7 and a borderline insignificant (p = 0.066) association with PD-L1 expression. Both CK7 and PD-L1 expression were negative prognostic indicators as well. We believe that SATB2 and CK7 immunohistochemistry will allow for the identification of particularly aggressive CRC phenotypes.

CK7, its negative prognostic role, and association with traditional factors indicating a poor prognosis (particularly stage) have been documented in several studies, including ours^26,27,32,34,35,57^. Some recent studies shared the methodology in terms of 10% positivity cut-off value to regard the tumor CK7 positive^34,35,57^. According to Fei et al.^35^ and Kirchner et al.^65^, CK7 expression may be regarded as a hallmark of retro-differentiation or dedifferentiation related to the re-acquiring of a fetal phenotype, which is linked to epithelial-mesenchymal transition that gives the tumor a capacity to metastasize. Kirchner et al. described a lack of glandular differentiation and a primitive duct-like morphology in gastric mucosa expressing CK7^65^. All these facts characterize CK7 expression in CRC as a dedifferentiation marker linked to more aggressive behavior.

PD-L1 has been widely studied in various types of cancer, and multiple immunohistochemistry assays have been approved as complementary diagnostics for patients with non-small cell lung cancer (NSCLC), melanoma, urothelial bladder cancer, esophageal, and gastric cancer^66,67^. In these tumors, PD-L1 is regarded as the target molecule of immune-checkpoint therapy (i.e., nivolumab, pembrolizumab), which significantly improves patient survival. Nevertheless, despite the wide use of PD-L1 expression, which has been shown by immunohistochemistry as a biomarker for PD-1/PD-L1 blockade in many types of cancer, there are growing concerns regarding its true predictability^68^. In CRC, the US Food and Drug Administration approved nivolumab as a treatment modality in MMR-deficient/MSI-H metastatic CRC refractory to fluoropyrimidine, oxaliplatin, and irinotecan^69^, although PD-L1 has not yet been approved as an immunotherapy predictive marker. Concerning the predictive value of tumor cell PD-L1 expression in CRC, results have been inconsistent in various studies, including meta-analyses. There are at least thirteen studies describing its ability to indicate a poor prognosis^44–56^, three studies indicating positive prognostic potential^70–72^, and five studies indicating no prognostic potential on CRC survival^73–77^. Among recent meta-analyses, we found two that described a negative impact^56,78^ and one describing almost no reliable prognostic value^79^.

Our PD-L1 expression analysis in CRC is in line with the ambiguous results of the cited studies: we found a slight (borderline insignificant with p-value = 0.15) negative impact on OS and no impact on CSS, but there was significantly shorter OS in cases that strongly (> 49%) expressed PD-L1 on tumor cell membranes. In terms of CSS, no significant results were obtained. Our CSS analysis failed most likely due to the low number of cases with strong PD-L1 expression.

The contradictory results of various studies can be explained by (1) overall paucity of CRCs expressing PD-L1 even in larger datasets, i.e., usually occurring in < 10% of probands, making it hard to reach statistical significance; (2) different methods of PD-L1 expression assessment either in terms of methodology (tumor proportion score—TPS, combined positive score—CPS, immune cell score—IC), or in terms of different antibodies (e.g., clones 22C3, and SP263), or in terms of percentage threshold value to regard a tumor as positive. In our study, we used a standardized diagnostic kit (SP263), and TPS was assessed in line with the manufacturer’s instructions. The results of our study suggest a minimal prognostic impact for weak PD-L1 expression. Our data clearly document that only membranous PD-L1 expression, in a substantial subset (≥ 50%) of malignant cells, is linked to poor survival. From the biological point of view, this may be explained by PD-L1 on tumor cell surfaces switching off cytotoxic T-lymphocytes, thus enabling the tumor cell to escape anti-tumor immunity. In NSCLC, several studies documented variable effects of therapeutic immune-checkpoint inhibitors (pembrolizumab) depending on the percentage of tumor cells expressing PD-L1 (as assessed through immunohistochemistry and using positivity cut-off values of ≥ 1%^80,81^ or ≥ 50%^82^. The results of our research indicate using either higher cut-off value than 1% for indicating a CRC as PD-L1+ for patient prognostic stratification, despite being limited by a small number of patients with CRC expressing PD-L1.

CRC may be categorized into two distinct groups according to molecular genetic pathways of carcinogenesis. The first involves chromosomal instability (CIN) and the second is characterized by microsatellite instability (MSI)^83^. The CIN pathway concerns about 80% of sporadic CRCs that are characterized by frequent allelic imbalance, chromosomal amplifications, deletions and translocations. The minority of CRCs arise due to MSI caused by hereditary or sporadic mutational or epigenetic deactivation of one from the four most common genes involved in the process of DNA reparation coding DNA mismatch repair (MMR) proteins (MLH1, MSH2, MSH6, PMS2). MSI CRCs are characterized by failed DNA repair resulting in high load of tumor mutation burden. As a result of the high mutation burden of MSI CRC, the tumor cells present multiple neoantigens (i.e. PD-L1)—in line with this theory and several previous studies^46,76,84^, our data document a significant PD-L1 overexpression in MMR-deficient CRC tumor cells.

Although bringing a novel finding (association of two prognostic markers SATB2 and CK7), there are several limitations in our study. Like in all studies using time and cost sparing TMA technique, the question of tumor heterogeneity cannot be ignored. However, microarray technique showed good correlation with whole section immunohistochemistry in a comparative study, and it was previously validated in CRC for several markers^85^. As described in results section, we obtained very good intercore reliability rate. Interobserver variability in immunohistochemical markers with frequent low expression (particularly PD-L1) may represent a potential pitfall in our study. Nevertheless, PD-L1 showed good interobserver agreement in gastric cancer^86,87^, in NSCLC^88^, in breast cancer^89^, and very recently in multiple tumors^90^ in various antibody clones in both TPS and CPS. Although no validation study has been published on CRC, probably due to low number of CRC expressing PD-L1, we can consider PD-L1 clone SP263 a reliable method with good interobserver agreement, as we obtained kappa values around 0.8 documenting substantial interobserver agreement.

As already mentioned, all the three markers examined in our study (SATB2 low status, CK7 expression, and strong PD-L1 positivity) can serve for prognostic stratification of patients suffering from CRCs in all stages. However, the “aggressive CRC phenotype” may be characterized by dedifferentiation, i.e., the loss of the differentiation marker, production of aberrant cytokeratin 7, and eventual high production of a neo-antigen, i.e., PD-L1. The exact link between the three markers is hard to explain and needs further investigation at the molecular level. We suggest that the loss of constitutive colon-specific protein (SATB2) and the gain of aberrant non-colon molecules CK7 and PD-L1, i.e., possibly via genomic instability or a high mutation load, may be involved and needs to be further explored. Our study was limited by the overall paucity of CK7+ and PD-L1+ CRCs; eventual corroboration of these associations in larger patient cohorts will be of great interest.

## Supplementary Information

Supplementary Legends.

Supplementary Figure 1.

Supplementary Figure 2.

Supplementary Figure 3.

Supplementary Figure 4.

Supplementary Figure 5.

Supplementary Figure 6.

Supplementary Table 1.

Supplementary Table 2.

Supplementary Table 3.

Supplementary Table 4.

Supplementary Table 5.

Supplementary Table 6.

Supplementary Table 7.

Supplementary Table 8.

Supplementary Table 9.

## Data Availability

All research data are available in the supplementary (electronic only). If needed, please contact the corresponding author (jan.hrudka@lf3.cuni.cz) to obtain the data from this study.
