# Supplementary material for: Loss of SATB2 expression correlates with cytokeratin 7 and PD-L1 tumor cell positivity and aggressiveness in colorectal cancer
Source: Sci Rep. 2022 Nov 9;12:19152. doi: 10.1038/s41598-022-22685-0 (PMC9646713; doi:10.1038/s41598-022-22685-0)
Supplement: Supplementary file 1 — Supplementary Legends. [file 41598_2022_22685_MOESM1_ESM.doc]

**Supplementary material legends**

**Supplementary tables**

Supplementary Table 1 – excel table including all subjects and all analyzed variables.

Supplementary Table 2 – entire cohort – 5-years follow up - survival analysis - univariate Kaplan-Meier analysis with the log-rank test, restricted mean survival time, Cox regression. Significant p values are in bold.

Supplementary Table 3 – entire cohort – 10-years follow up - survival analysis - univariate Kaplan-Meier analysis with the log-rank test, restricted mean survival time, Cox regression. Significant p values are in bold.

Supplementary Table 4 – neoadjuvant therapy-naïve cohort – 5-years follow up - survival analysis - univariate Kaplan-Meier analysis with the log-rank test, restricted mean survival time, Cox regression. Significant p values are in bold.

Supplementary Table 5 – neoadjuvant therapy-naïve cohort – 10-years follow up - survival analysis - univariate Kaplan-Meier analysis with the log-rank test, restricted mean survival time, Cox regression. Significant p values are in bold.

Supplementary Table 6 – entire cohort – unweighted Cohen’s kappa test describing intercore reliability among two tumor samples in all three examined markers (SATB2, CK7, PD-L1) and interobserver reliability among pathologist 1 (JH) and pathologist 2 (RM).

Supplementary Table 7 – entire cohort – overall survival and cancer specific survival analysis according to percentage of PD-L1 expression - univariate Kaplan-Meier analysis with the log-rank test, restricted mean survival time, Cox regression. Significant p-value in bold.

Supplementary Table 8 – neoadjuvant-therapy naïve cohort – overall survival and cancer specific survival analysis according to percentage of PD-L1 expression - univariate Kaplan-Meier analysis with the log-rank test, restricted mean survival time, Cox regression. Significant p-value in bold.

Supplementary Table 9 – results of logistic regression in the neoadjuvant therapy-naïve cohort; significant and borderline insignificant associations are in bold, the main findings of the study are in bold and underlined – association of CK7 expression and SATB2 loss.

**Supplementary figures**

Supplementary Figure 1 – entire cohort; Kaplan Meier curves documenting 10-year overall survival (OS) in patients with CRC. Note decreased survival in patients with CRC with SATB2 low expression (a), CK7 high expression (b), PD-L1 expression (c), mismatch-repair proficient status (d), advanced stage (e), mucinous or signet ring cell morphology (f), high-grade tumors (g), and right-sided tumor localization (h). P values from the log-rank test < 0.05 indicate a significant difference in survival.

Supplementary Figure 2 – entire cohort; Kaplan Meier curves documenting worse 10-year cancer-specific survival (CSS) in patients with CRC with SATB2 low expression (a), CK7 high expression (b), mismatch-repair proficient status (d), advanced stage (e), high-grade tumors (g), and right-sided tumor localization (h). There is little impact of a mucinous or signet ring morphology (f), and no impact of PD-L1 expression (c). P values from the log-rank test < 0.05 indicate a significant difference in survival.

Supplementary Figure 3 – neoadjuvant therapy-naïve cohort; Kaplan Meier curves documenting 5-year overall survival (OS) in patients with CRC. Note decreased survival in patients with CRC with SATB2 low expression (a), CK7 high expression (b), PD-L1 expression (c), mismatch-repair proficient status (d), advanced stage (e), mucinous or signet ring cell morphology (f), high-grade tumors (g), and right-sided tumor localization (h). P values from the log-rank test < 0.05 indicate a significant difference in survival.

Supplementary Figure 4 – neoadjuvant therapy-naïve cohort; Kaplan Meier curves documenting worse 5-year cancer-specific survival (CSS) in patients with CRC with SATB2 low expression (a), CK7 high expression (b), mismatch-repair proficient status (d), advanced stage (e), high-grade tumors (g), and right-sided tumor localization (h). There no impact of a mucinous or signet ring morphology (f), and PD-L1 expression (c). P values from the log-rank test < 0.05 indicate a significant difference in survival.

Supplementary Figure 5 – neoadjuvant therapy-naïve cohort; Kaplan Meier curves documenting 10-year overall survival (OS) in patients with CRC. Note decreased survival in patients with CRC with SATB2 low expression (a), CK7 high expression (b), PD-L1 expression (c), mismatch-repair proficient status (d), advanced stage (e), mucinous or signet ring cell morphology (f), high-grade tumors (g), and right-sided tumor localization (h). P values from the log-rank test < 0.05 indicate a significant difference in survival.

Supplementary Figure 6 – neoadjuvant therapy-naïve cohort; Kaplan Meier curves documenting worse 10-year cancer-specific survival (CSS) in patients with CRC with SATB2 low expression (a), CK7 high expression (b), mismatch-repair proficient status (d), advanced stage (e), high-grade tumors (g), and right-sided tumor localization (h). There is no impact of a mucinous or signet ring morphology (f), and PD-L1 expression (c). P values from the log-rank test < 0.05 indicate a significant difference in survival.
