# Supplementary figures and images for: Loss of SATB2 expression correlates with cytokeratin 7 and PD-L1 tumor cell positivity and aggressiveness in colorectal cancer
Source: Sci Rep. 2022 Nov 9;12:19152. doi: 10.1038/s41598-022-22685-0 (PMC9646713; doi:10.1038/s41598-022-22685-0)

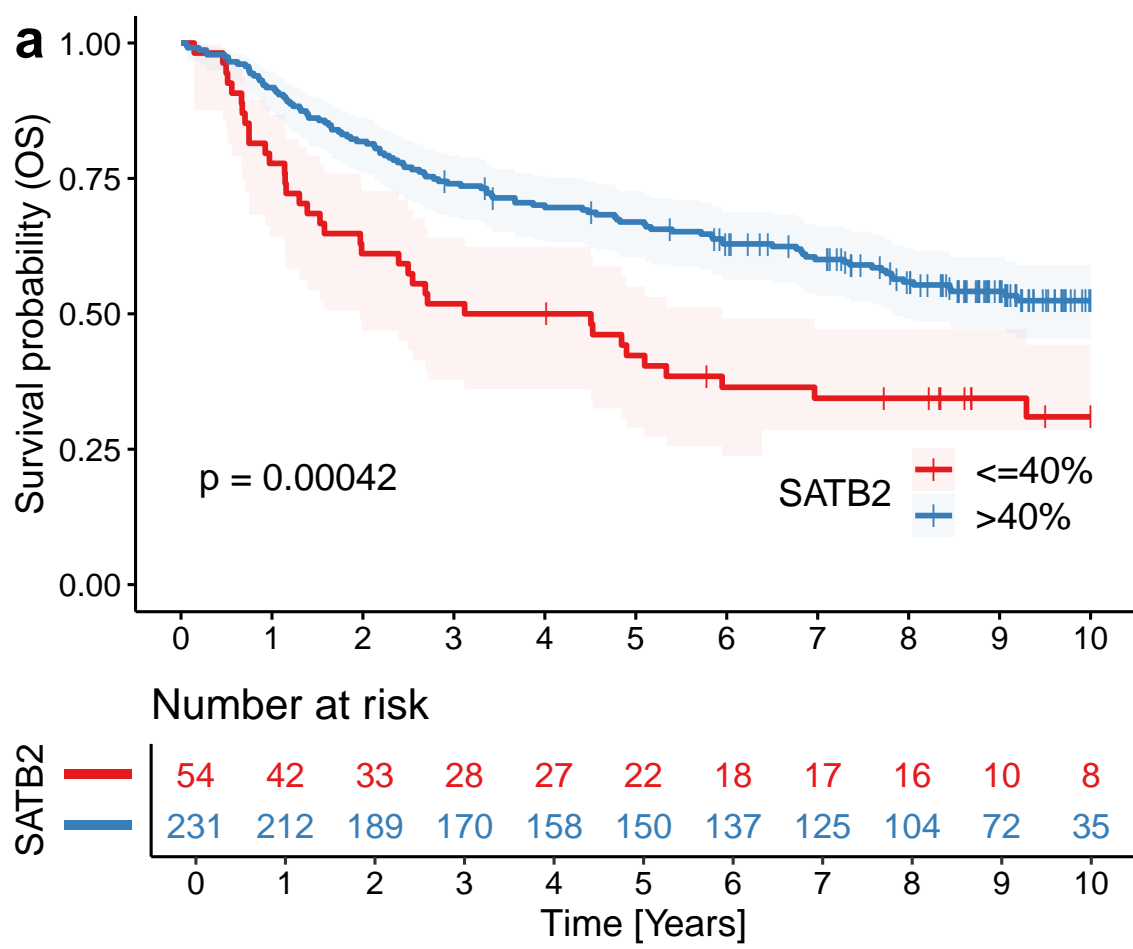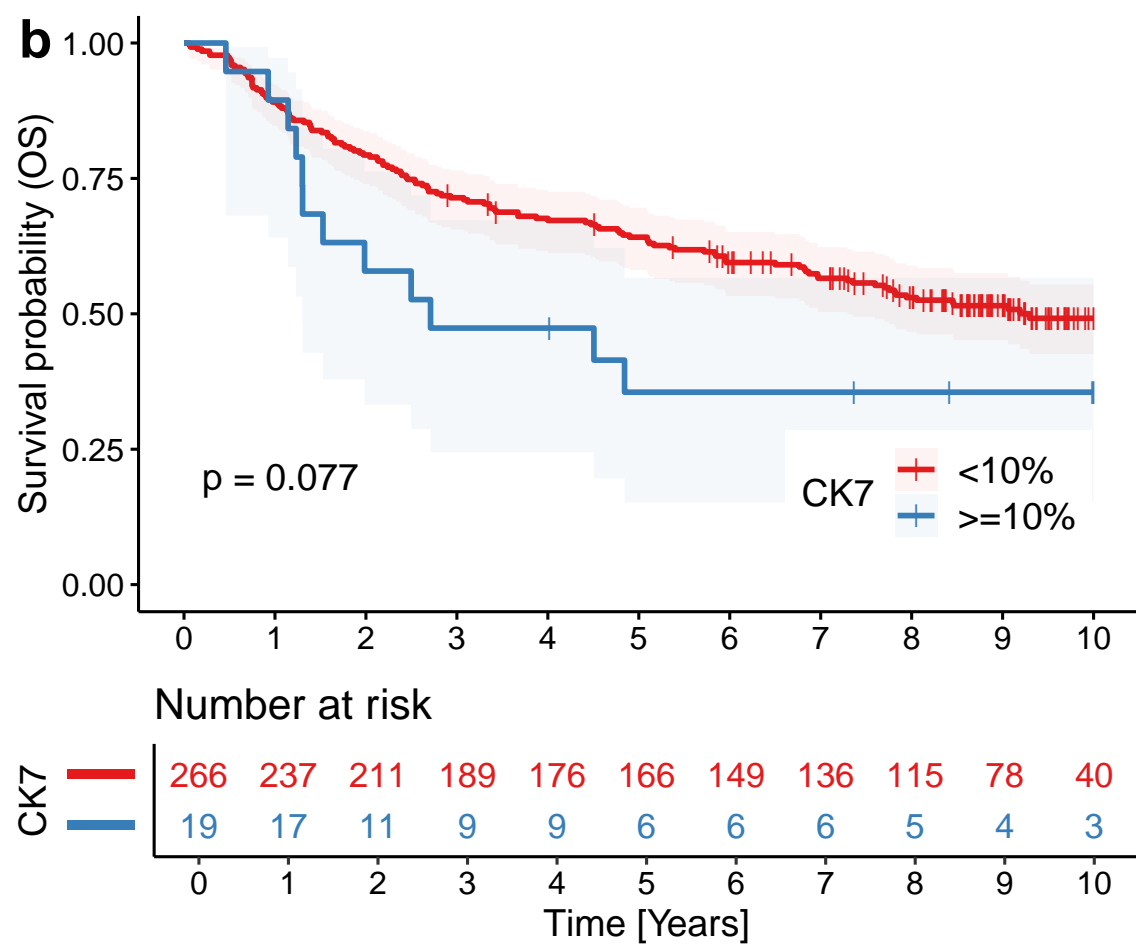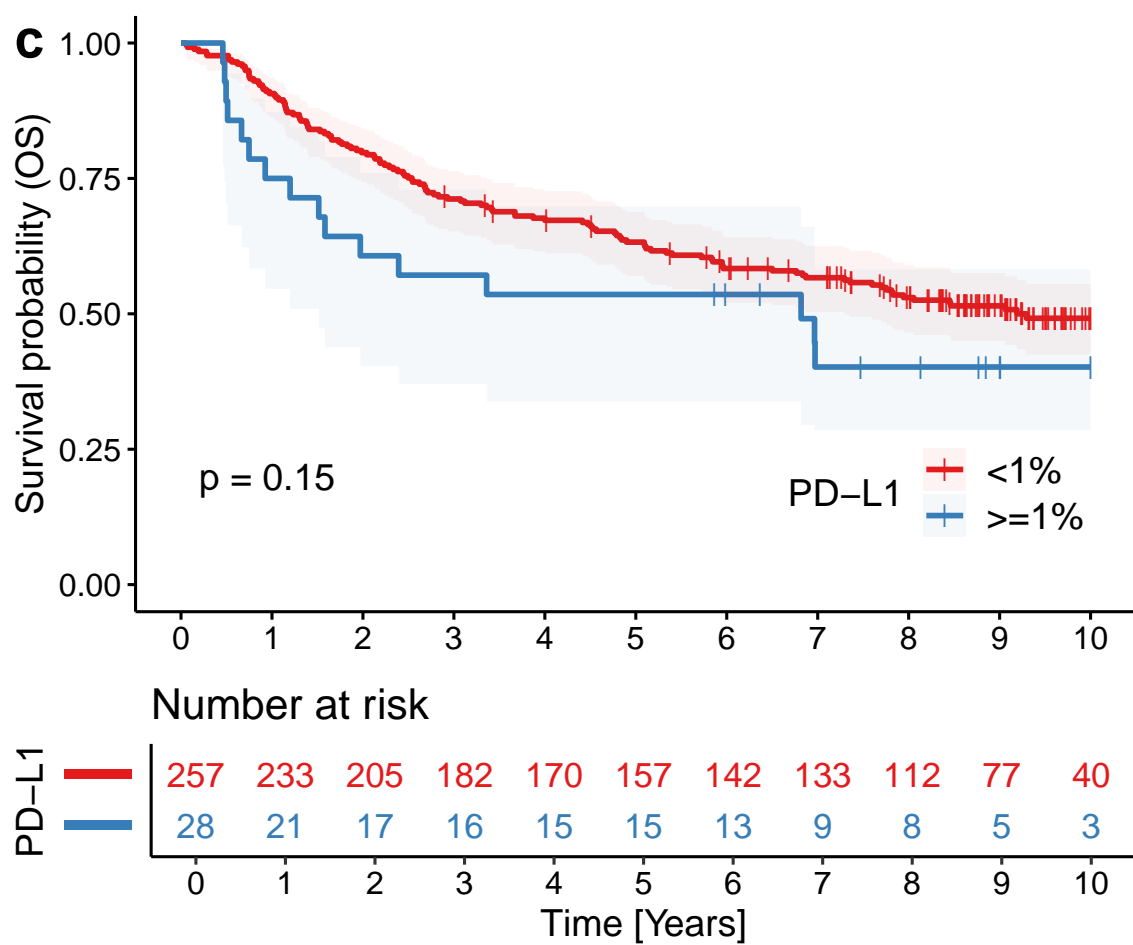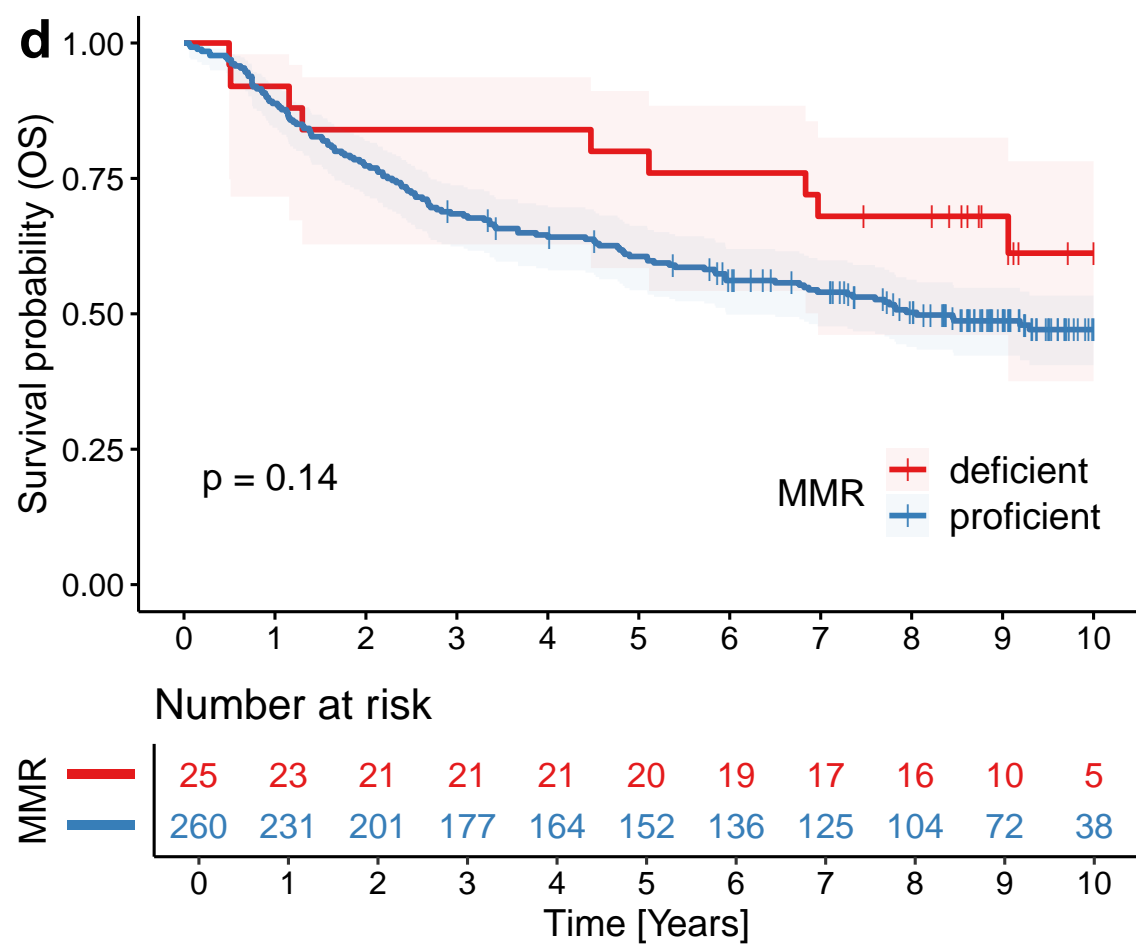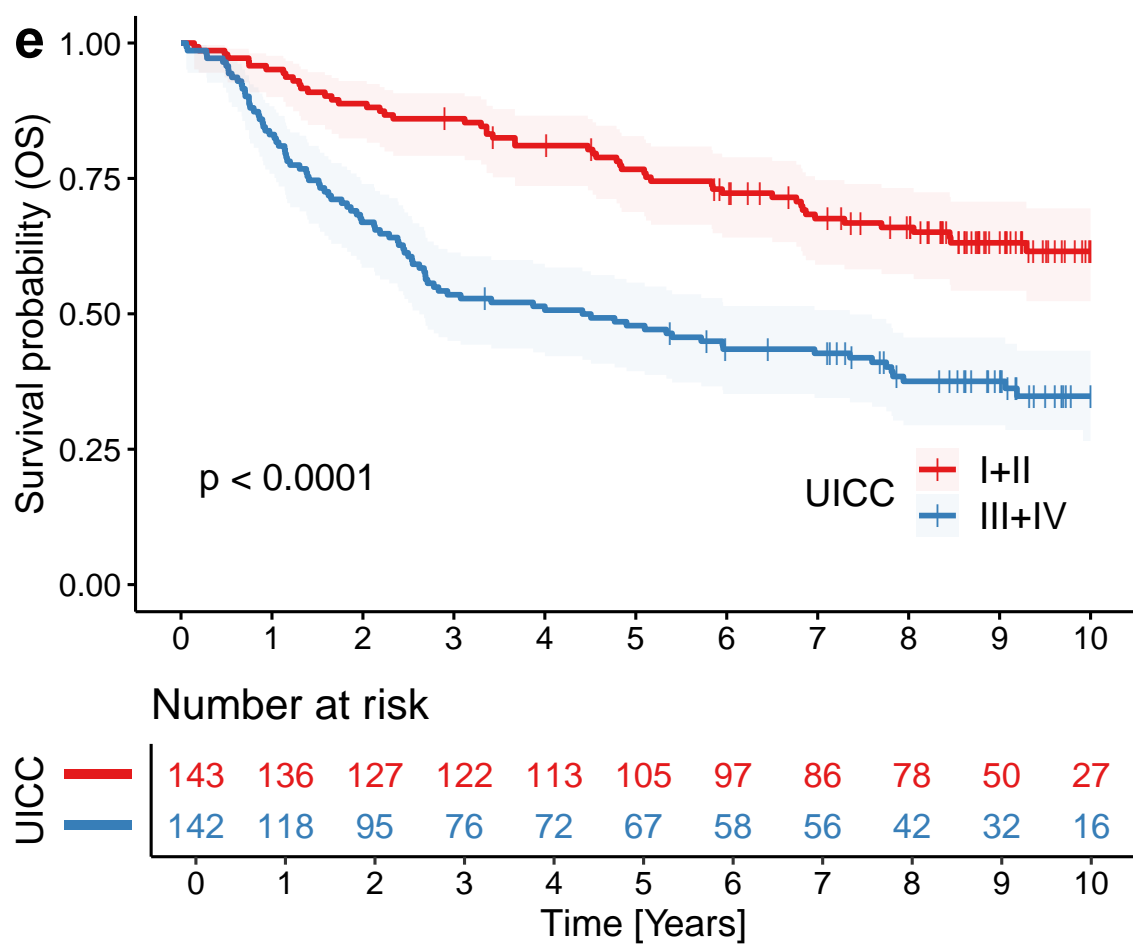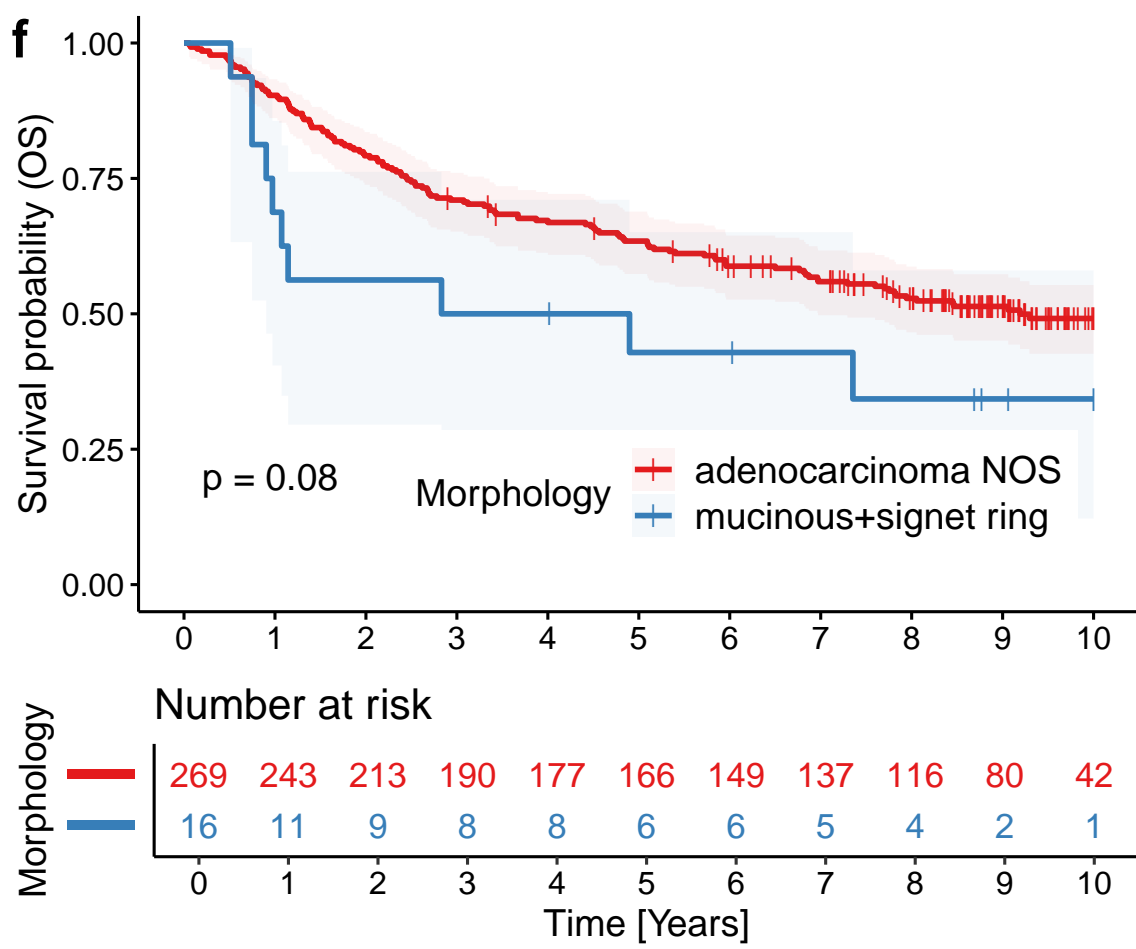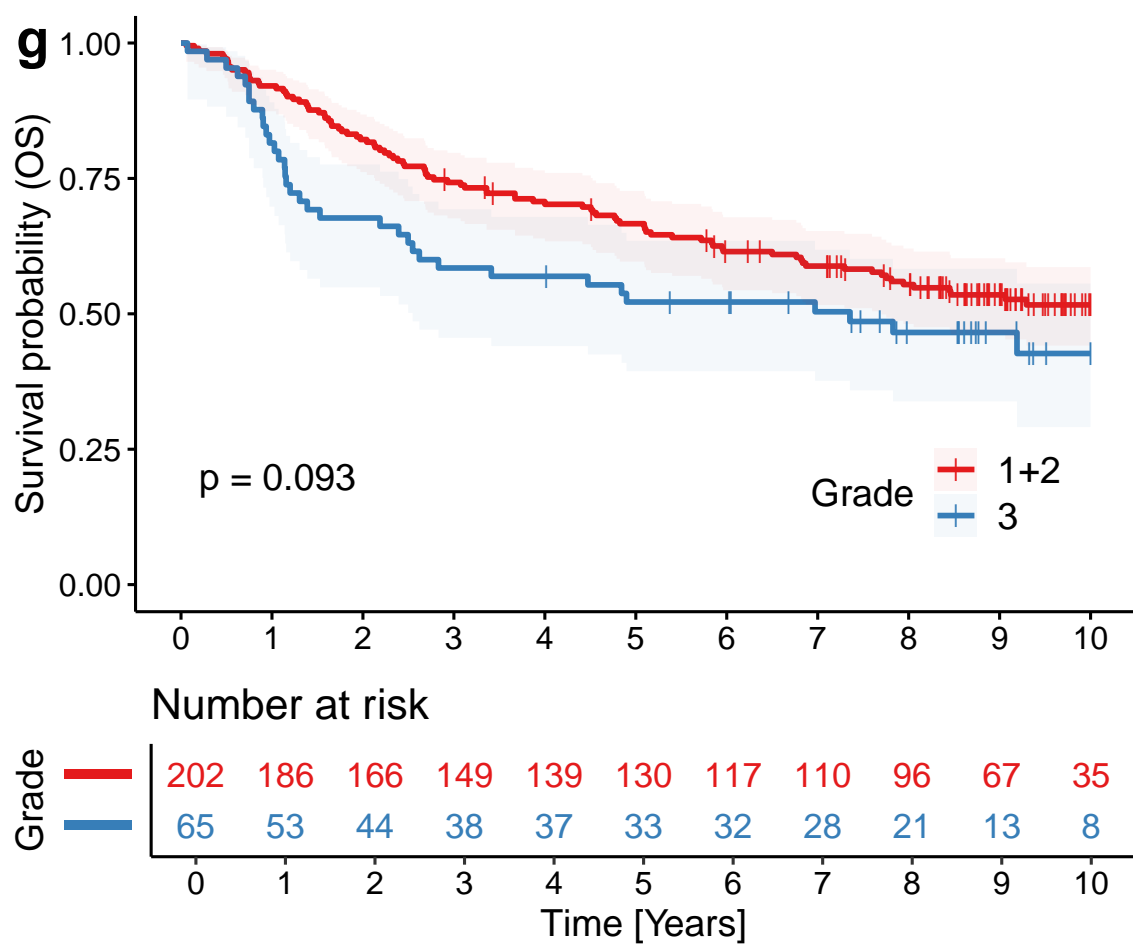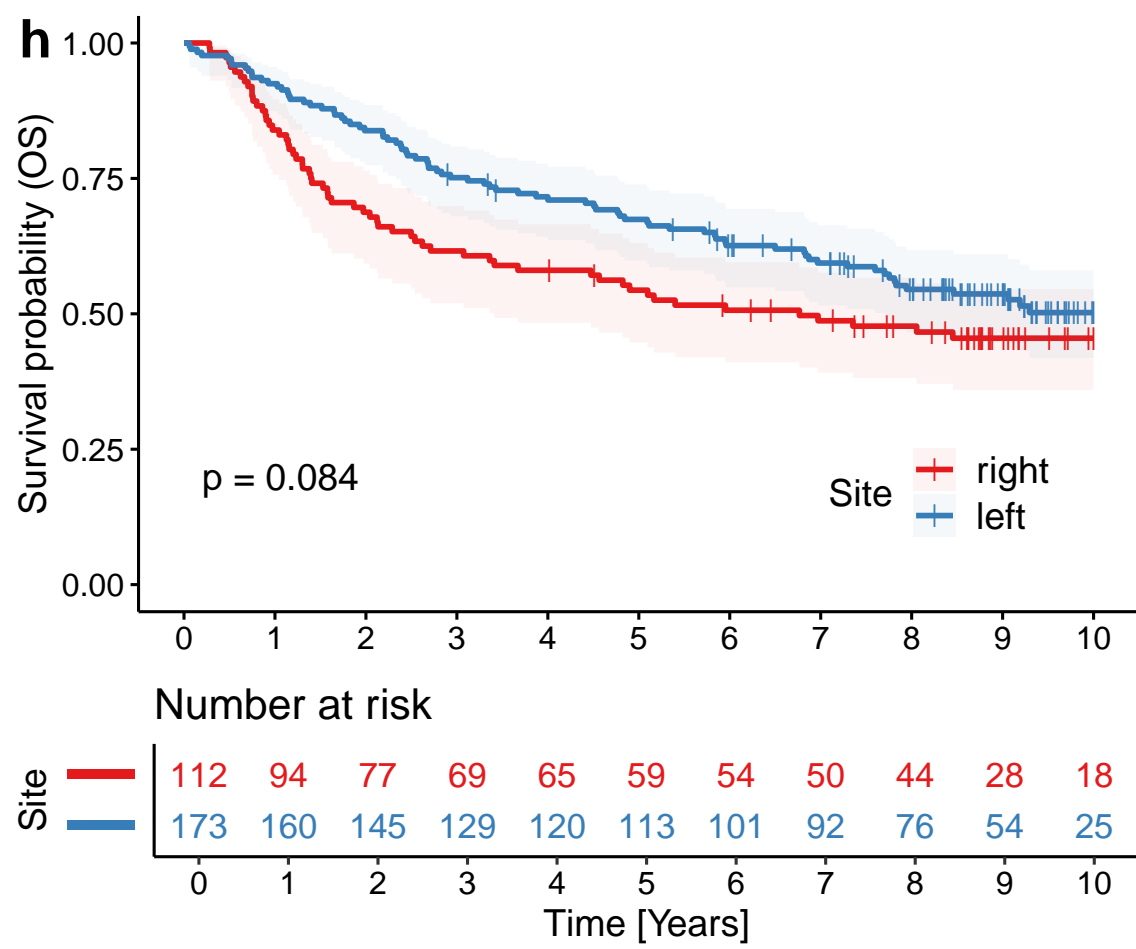

Supplement: Supplementary file 2 — Supplementary Figure 1. [file 41598_2022_22685_MOESM2_ESM.pdf]

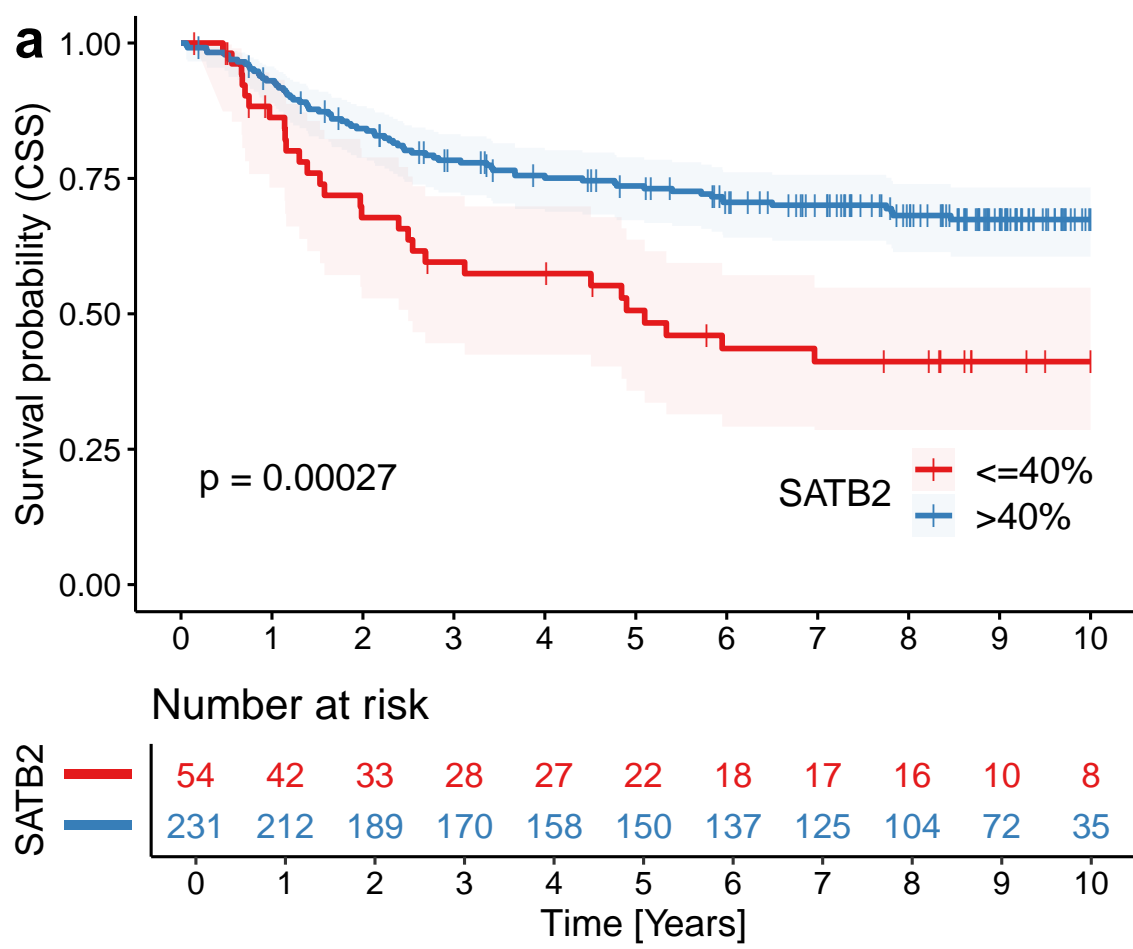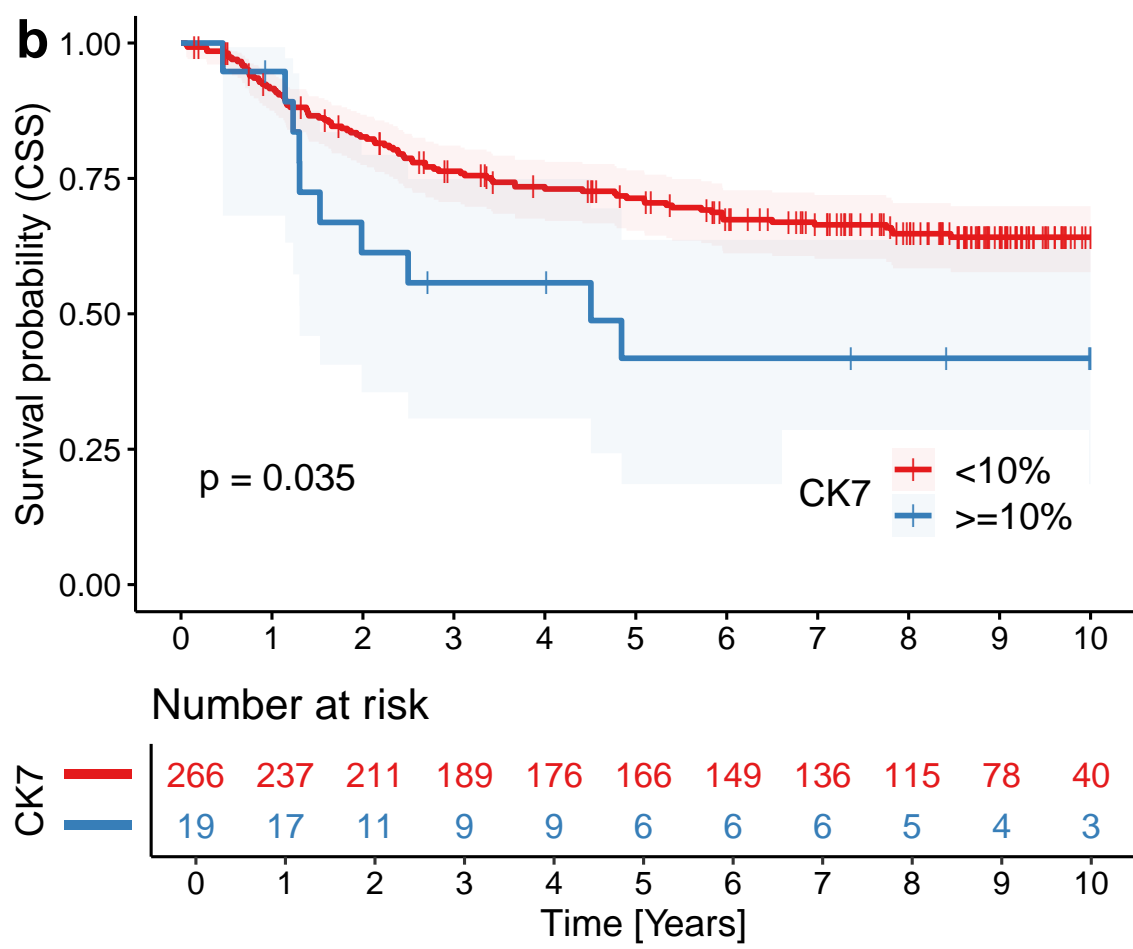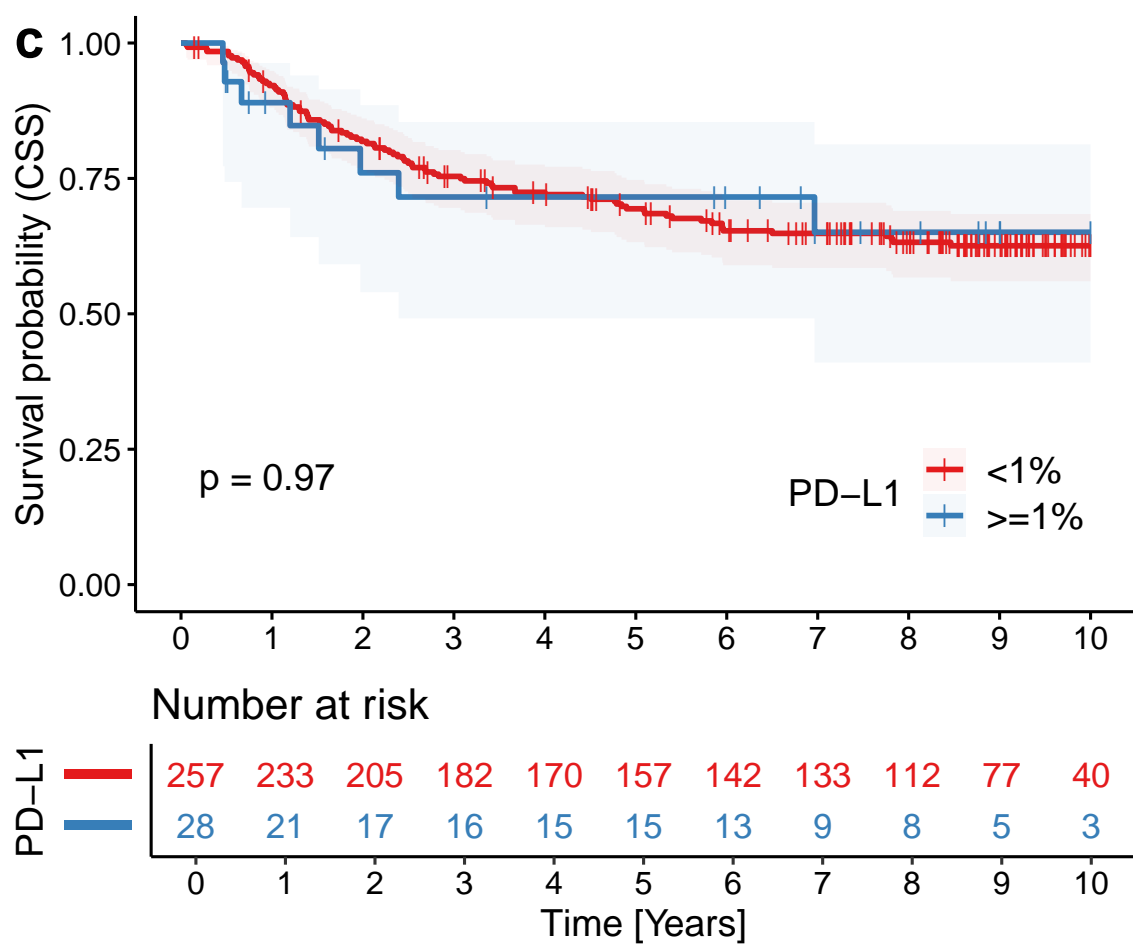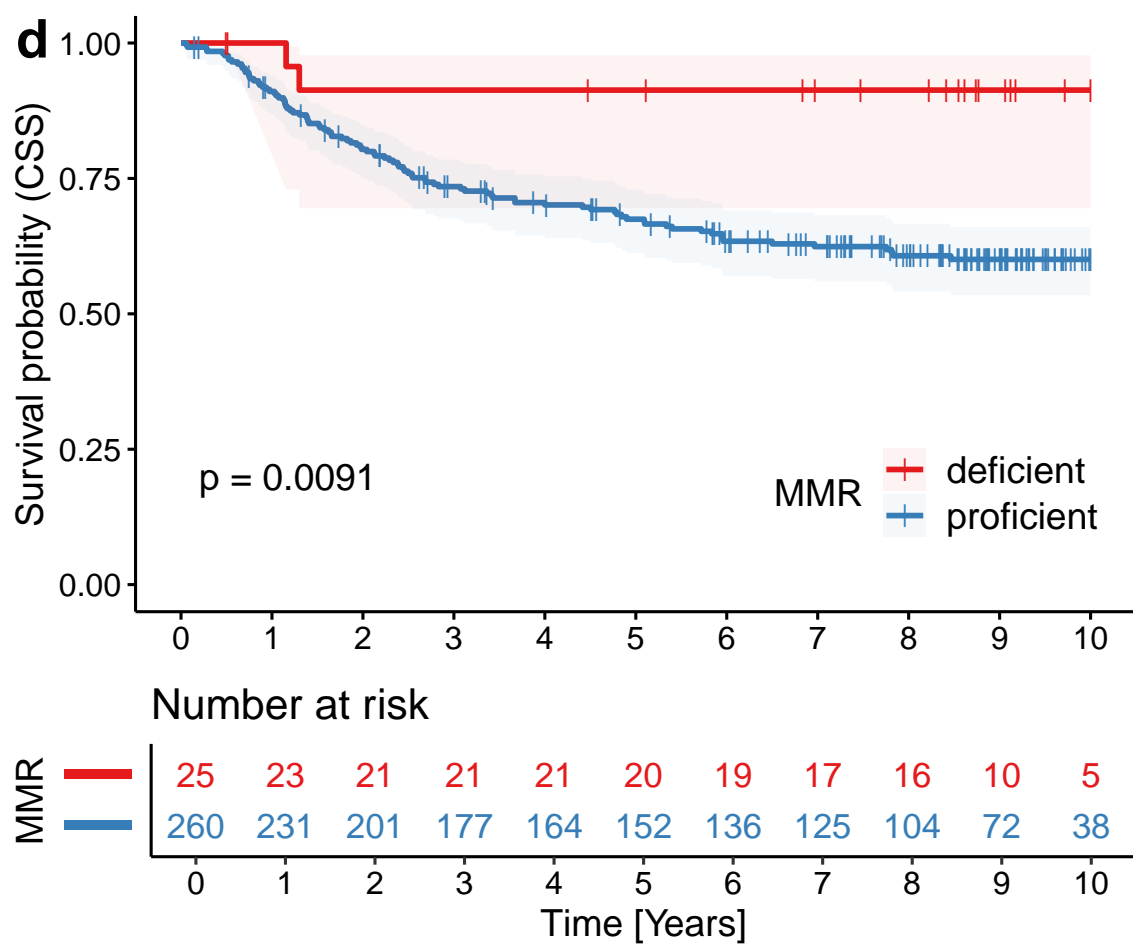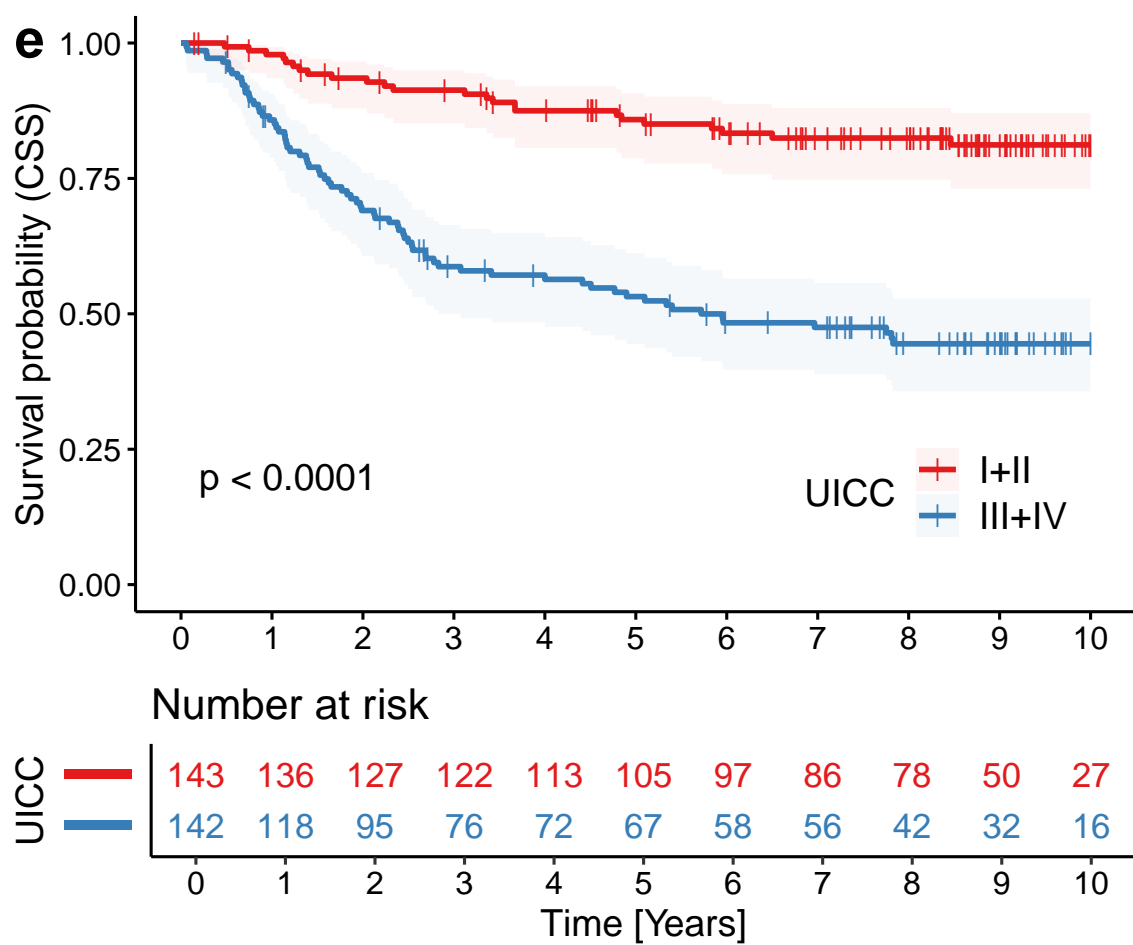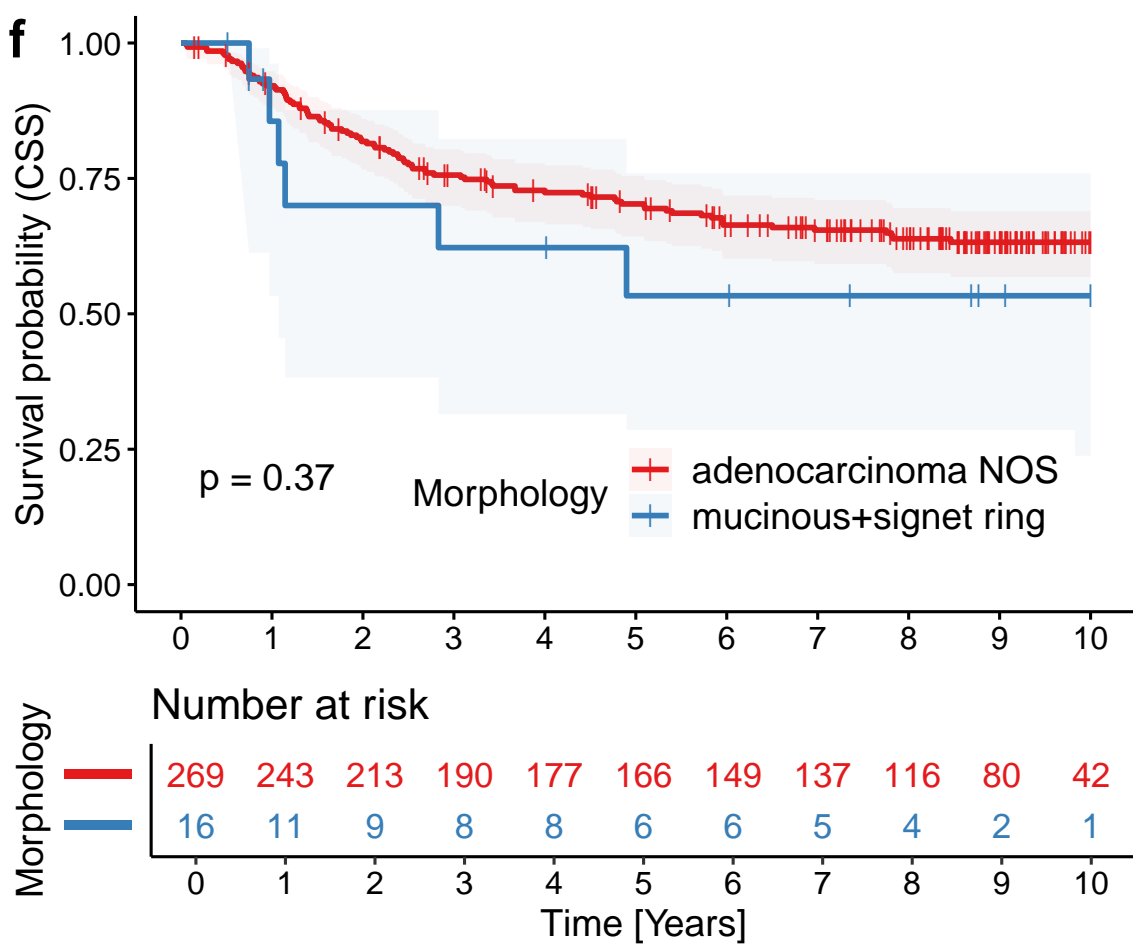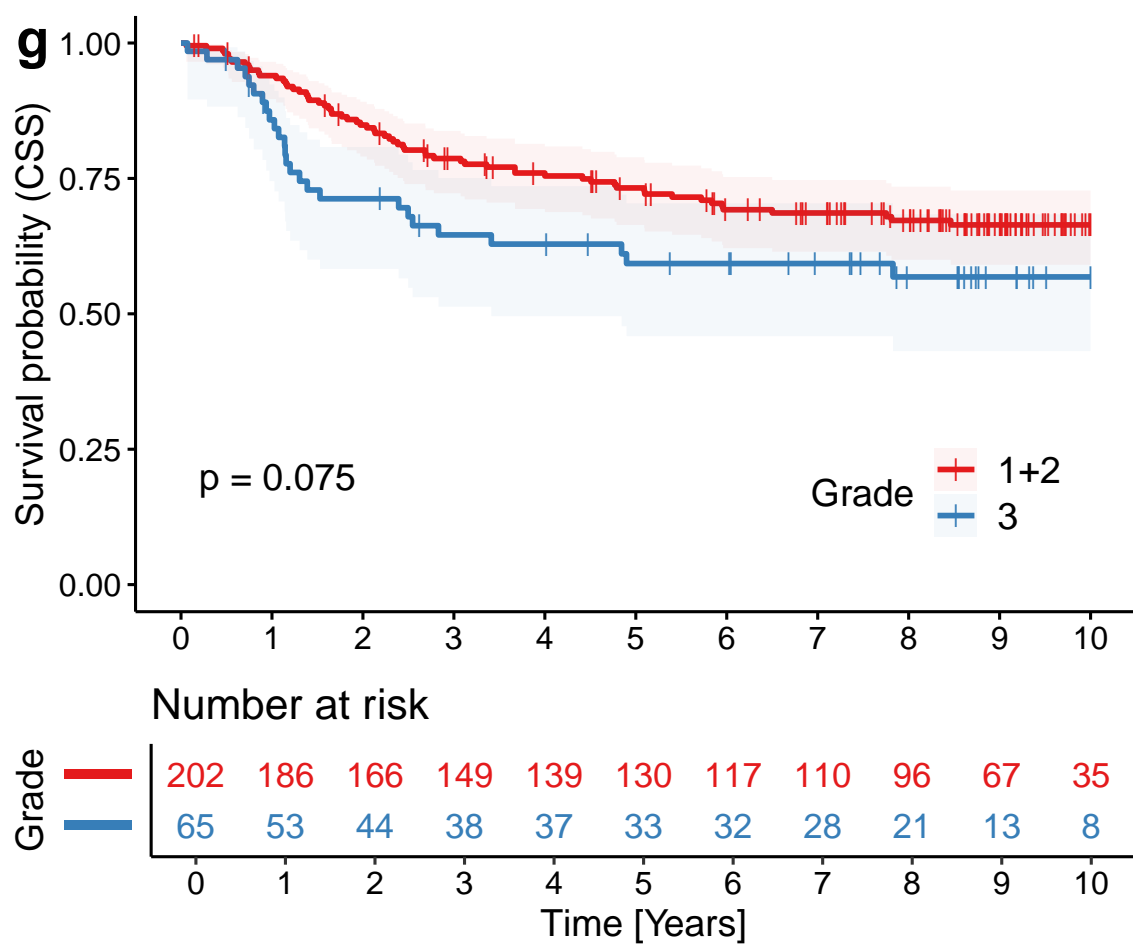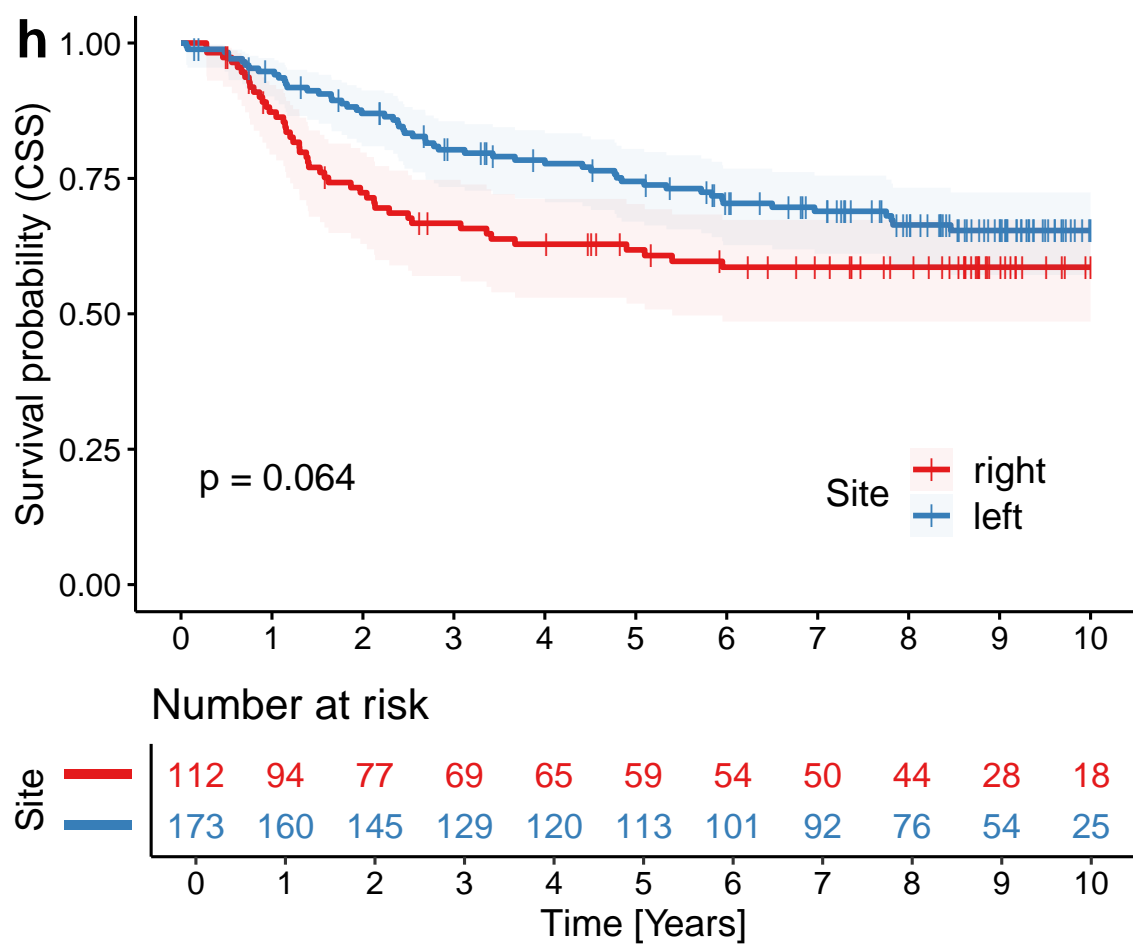

Supplement: Supplementary file 3 — Supplementary Figure 2. [file 41598_2022_22685_MOESM3_ESM.pdf]

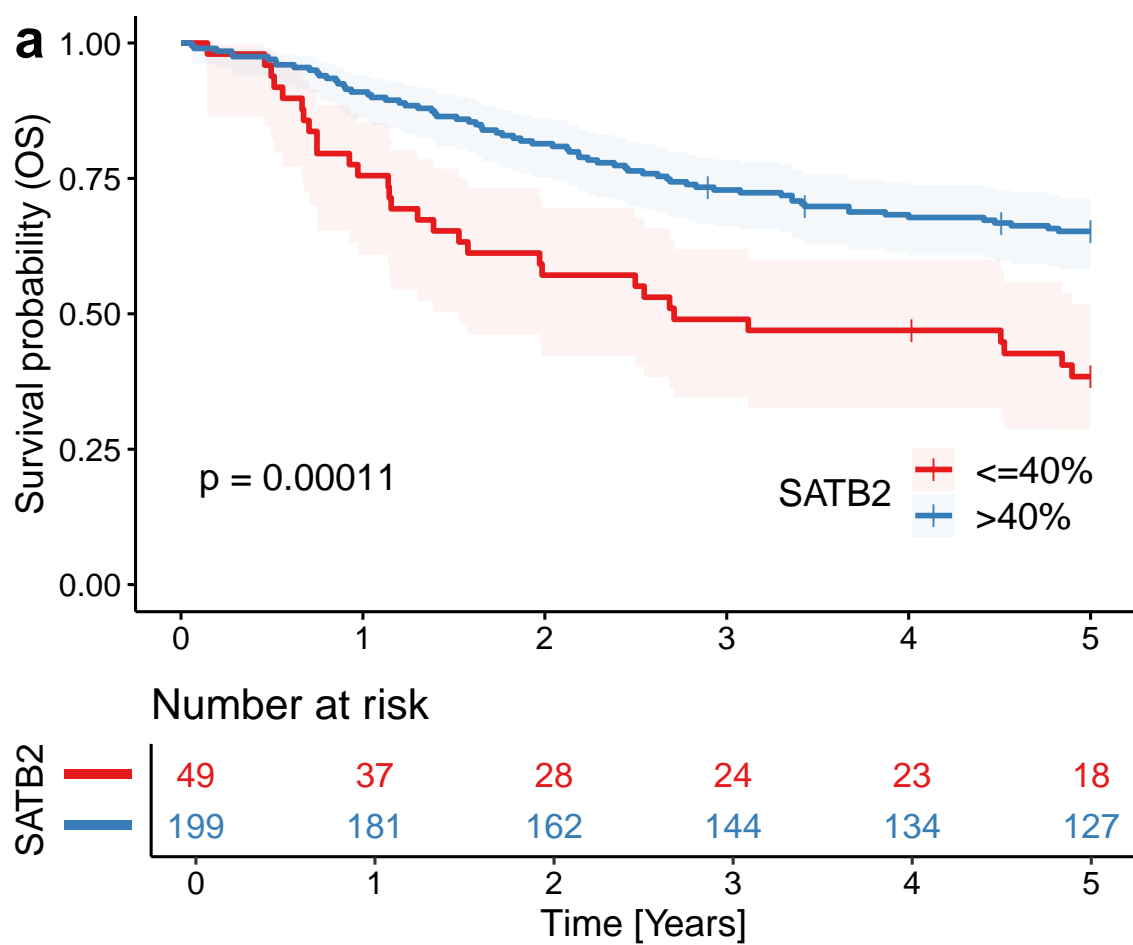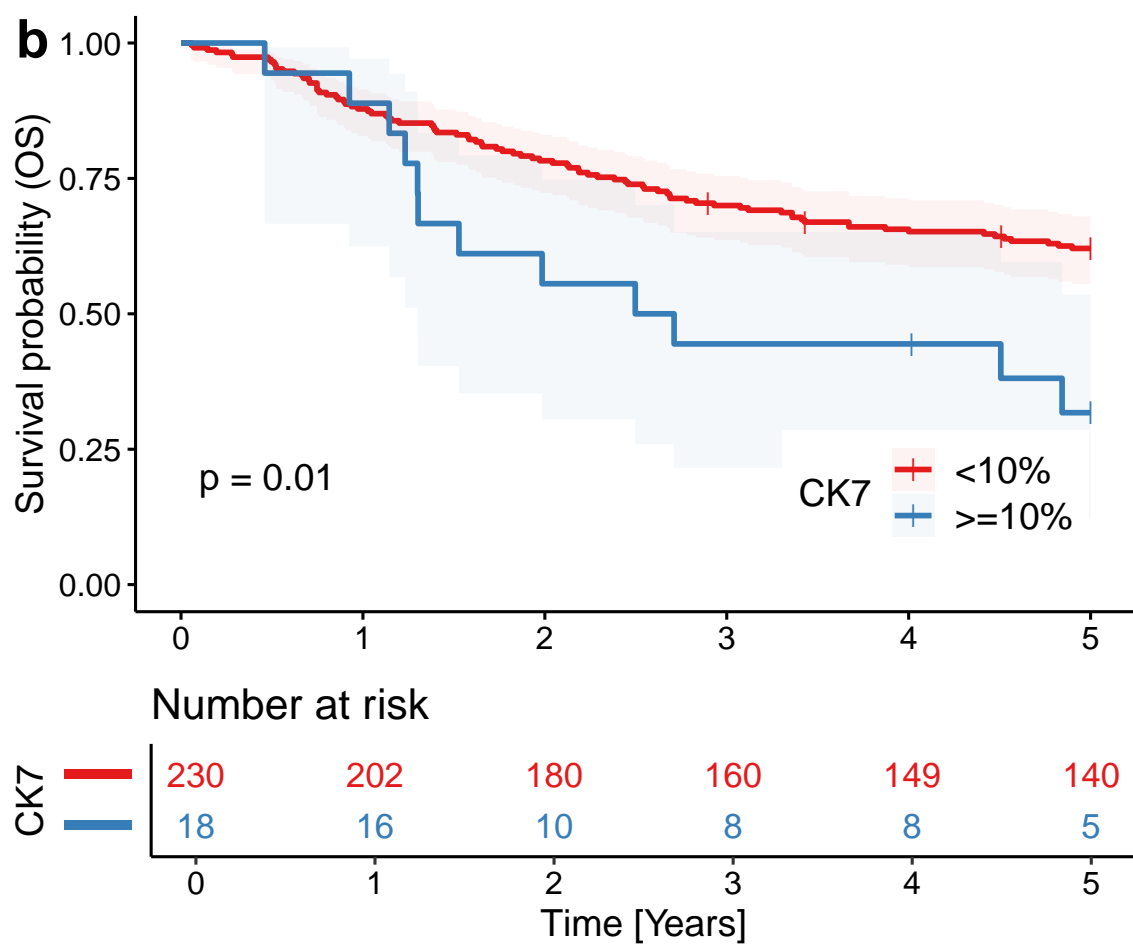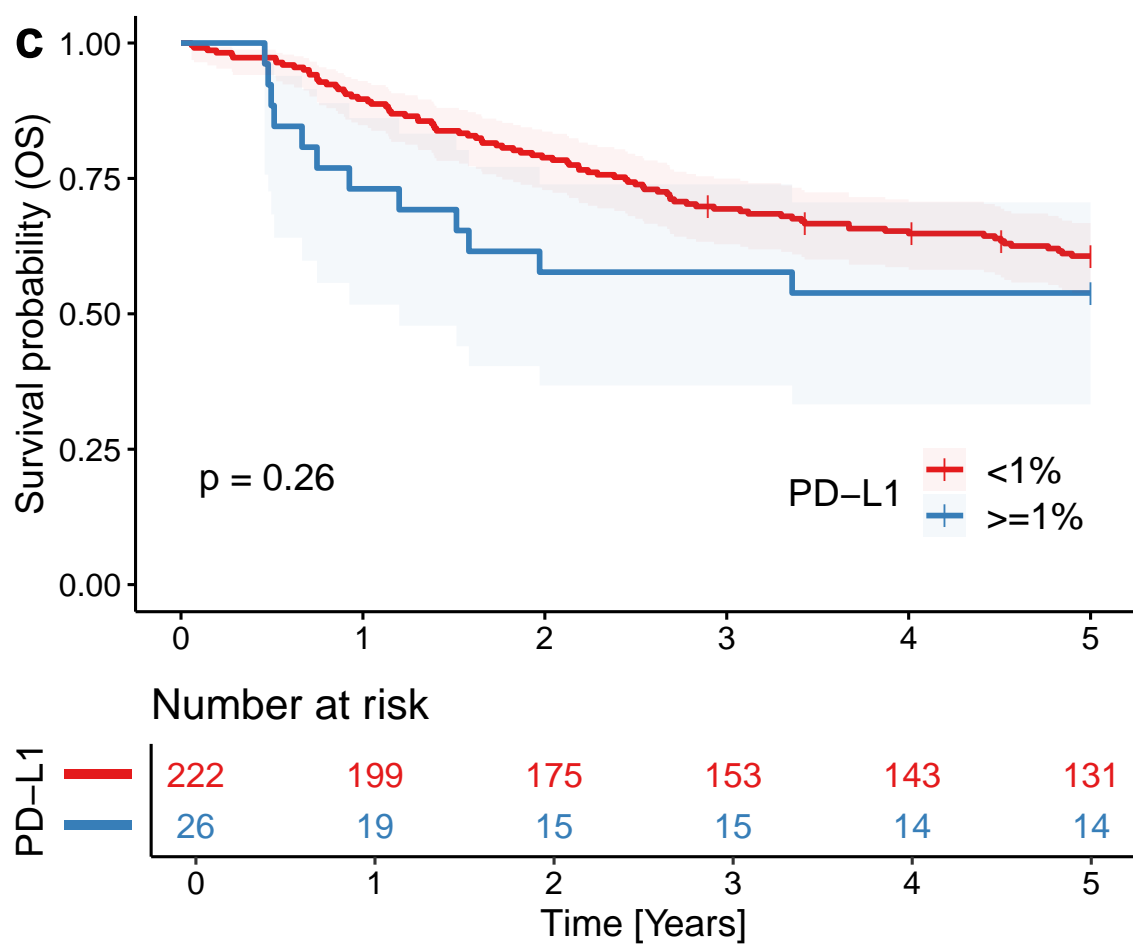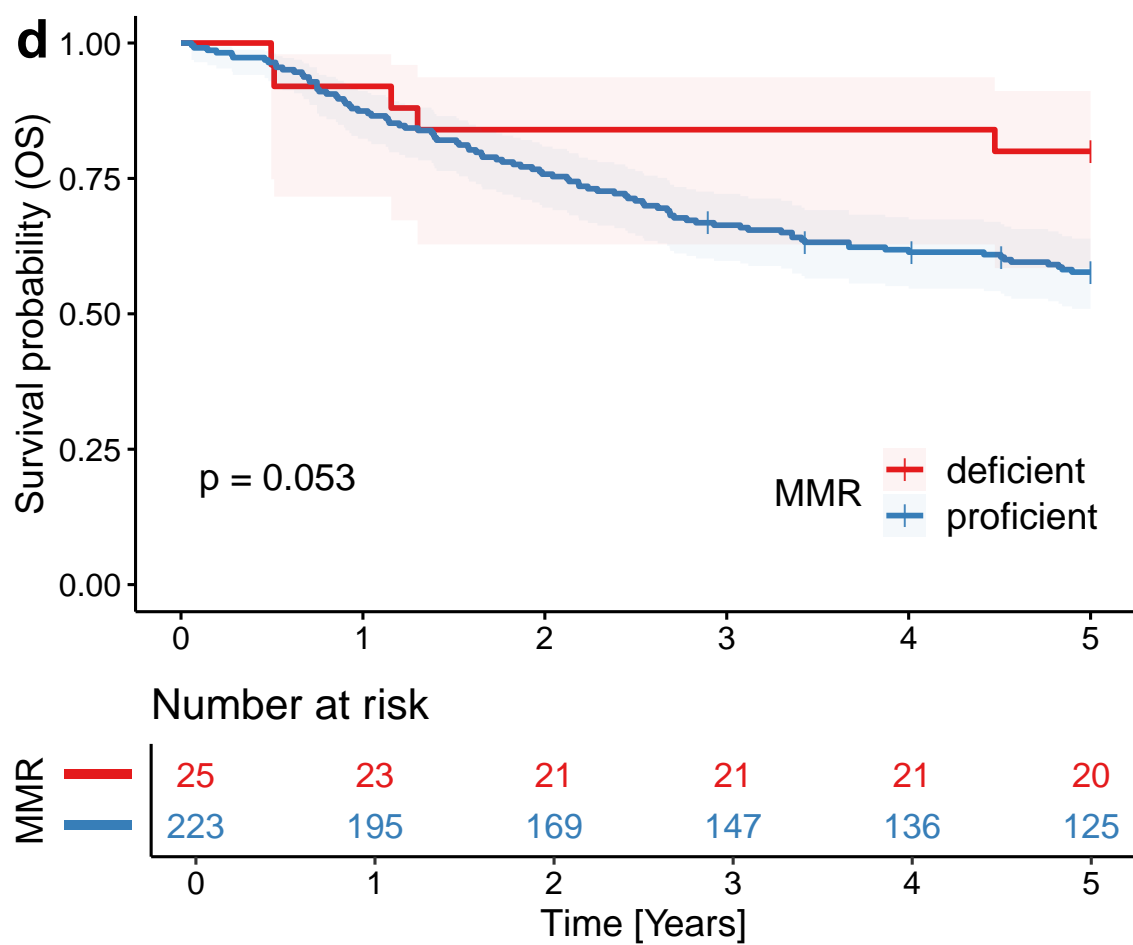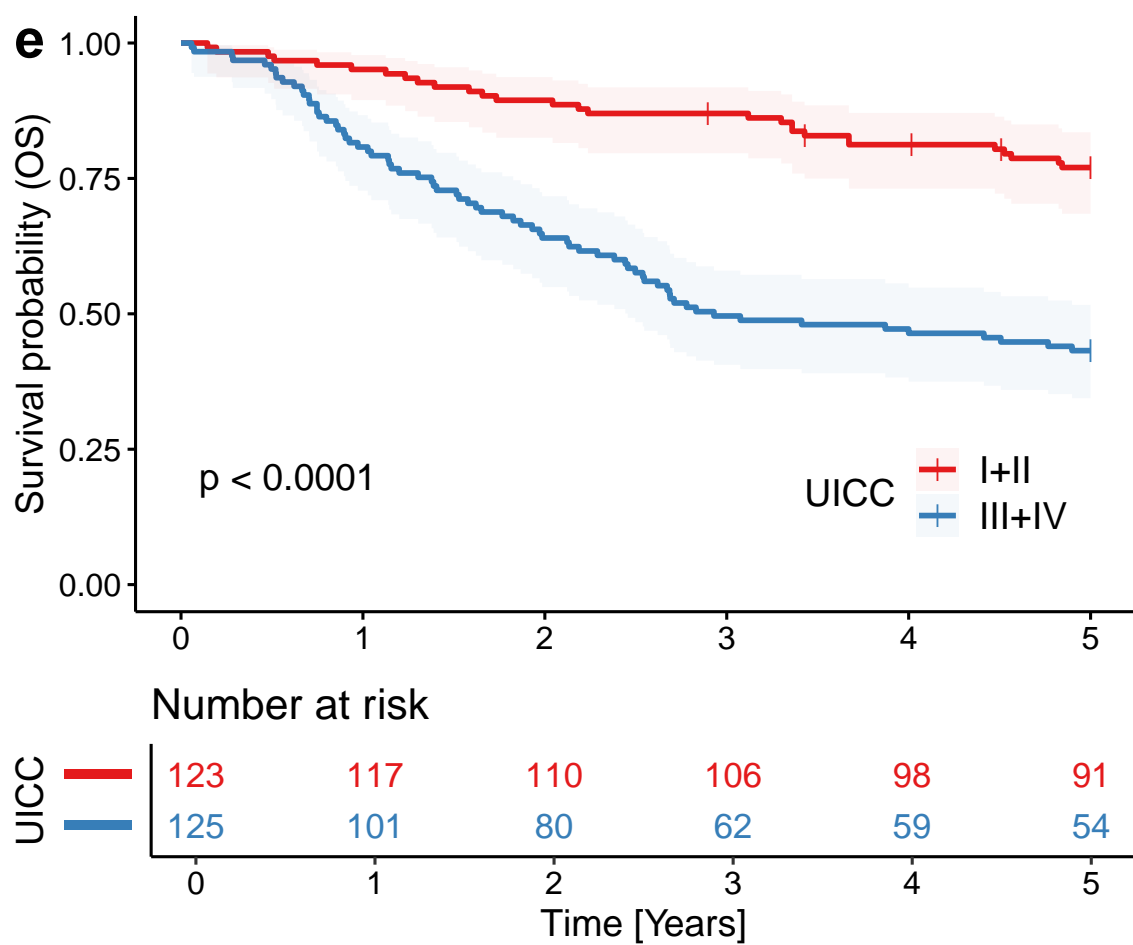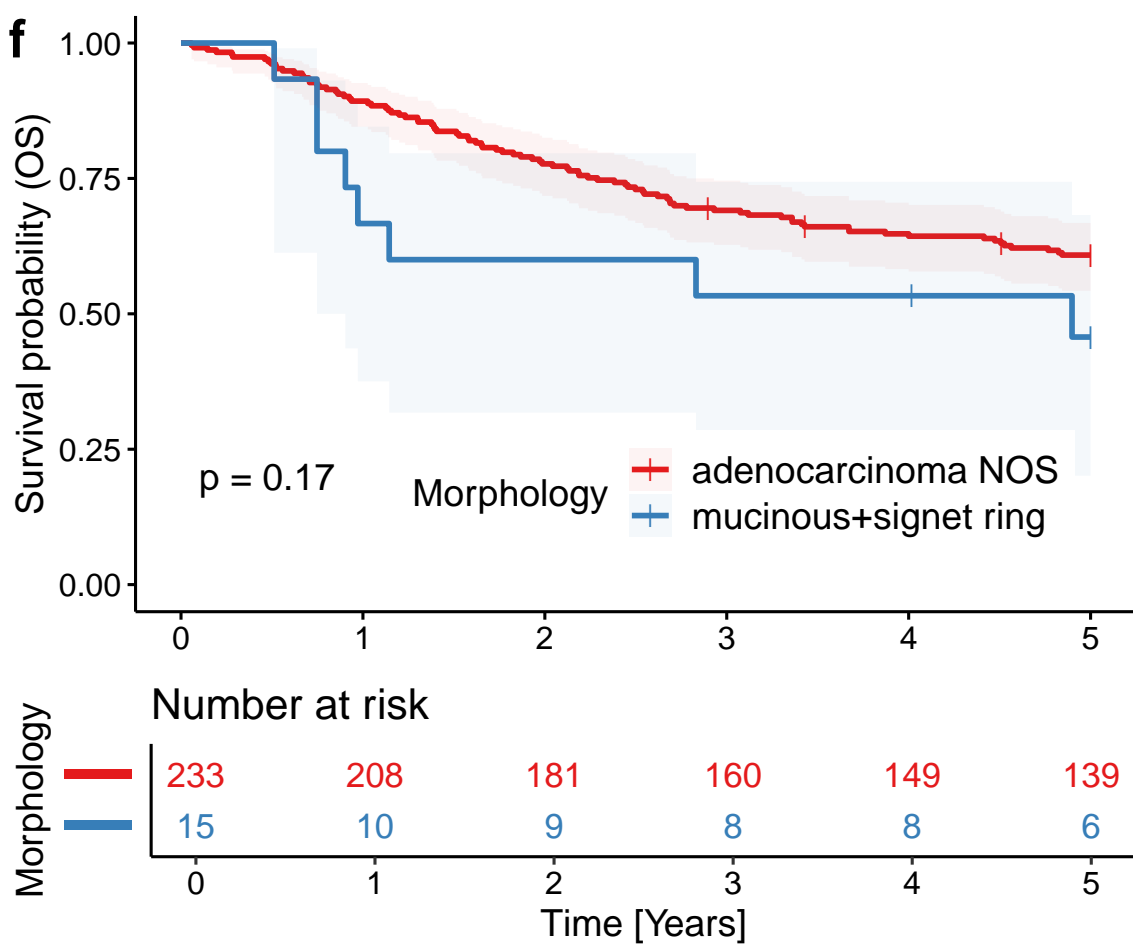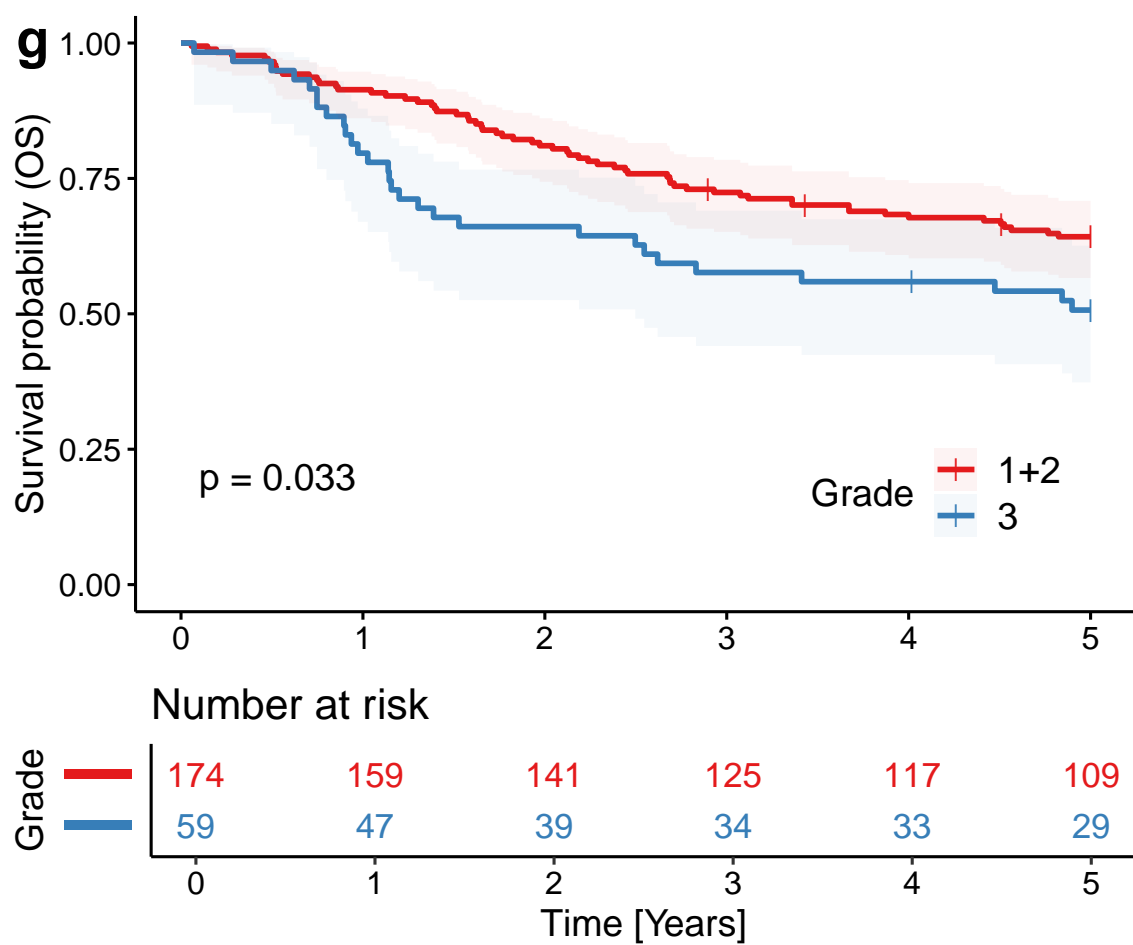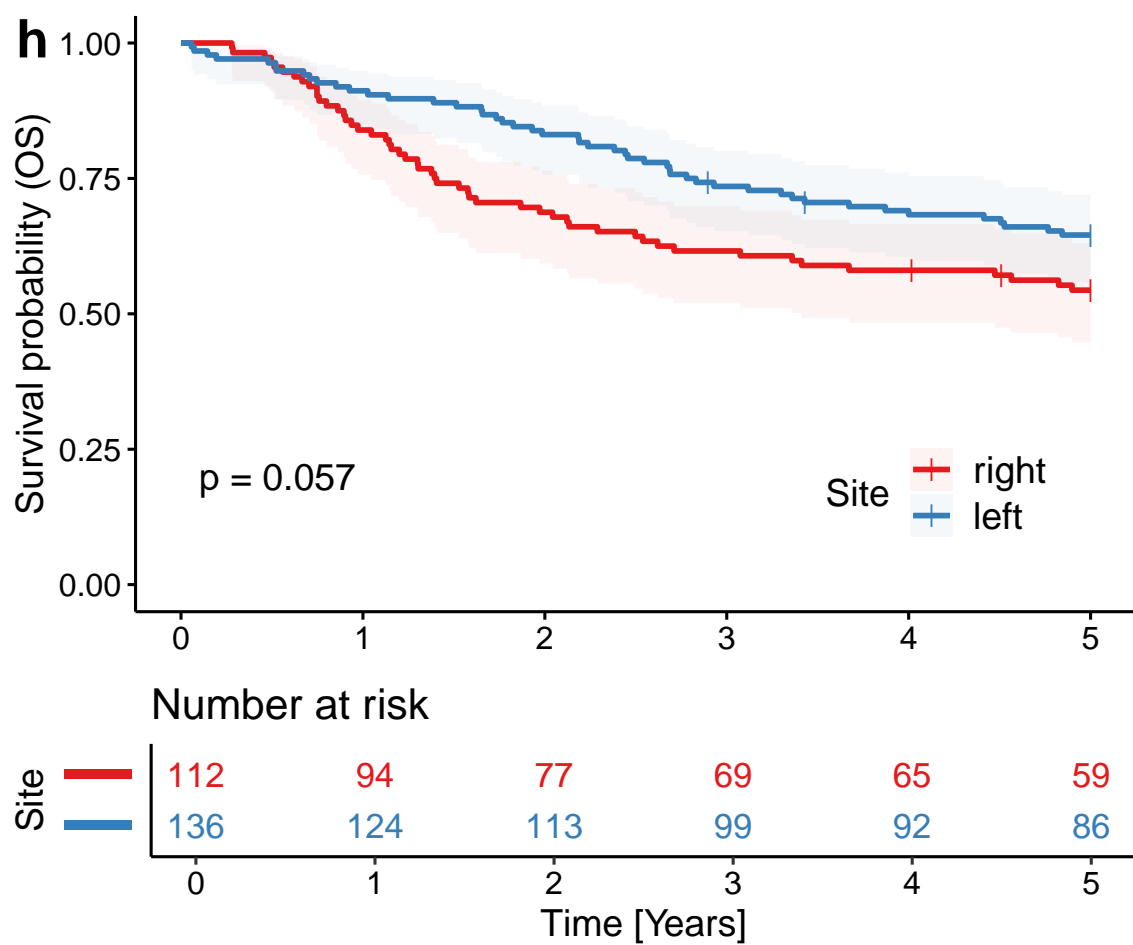

Supplement: Supplementary file 4 — Supplementary Figure 3. [file 41598_2022_22685_MOESM4_ESM.pdf]

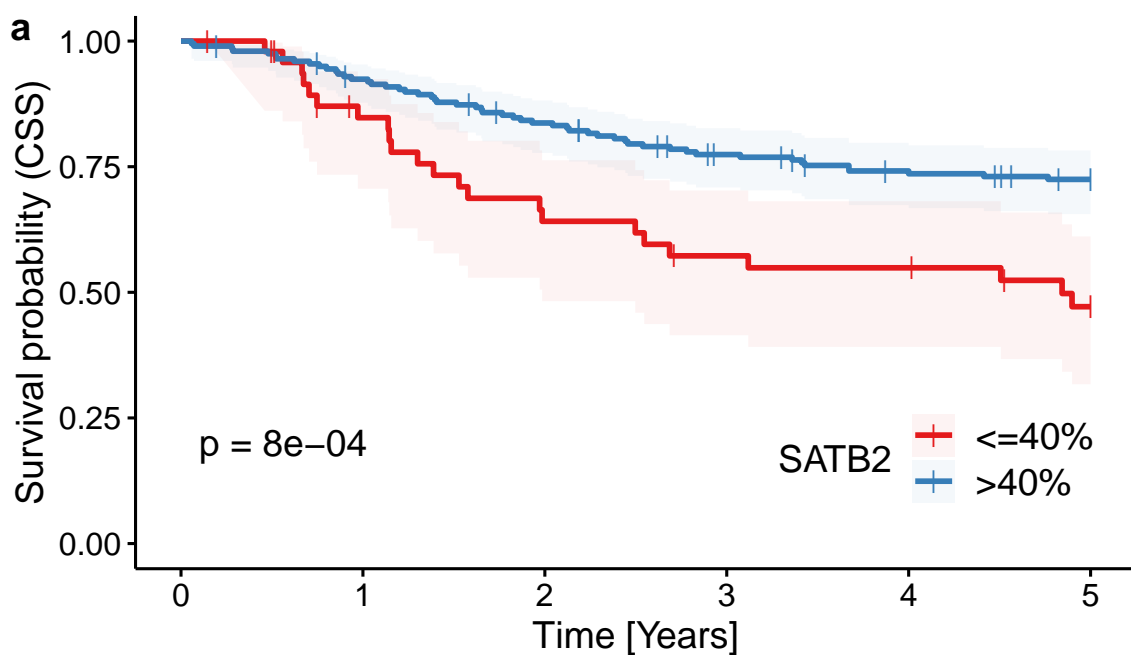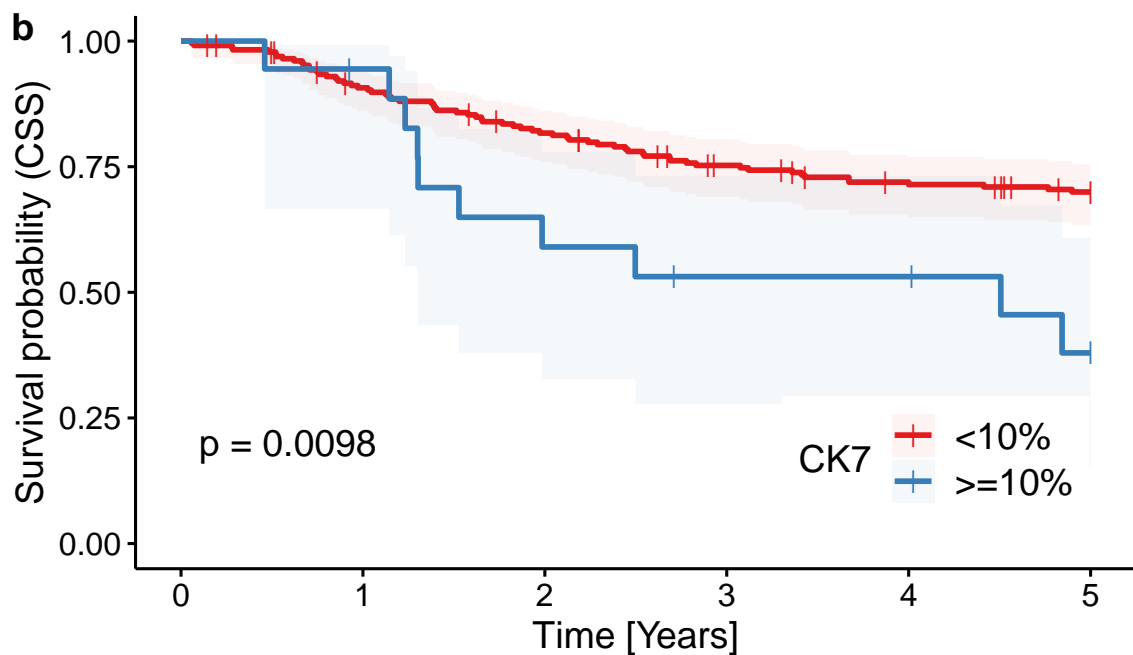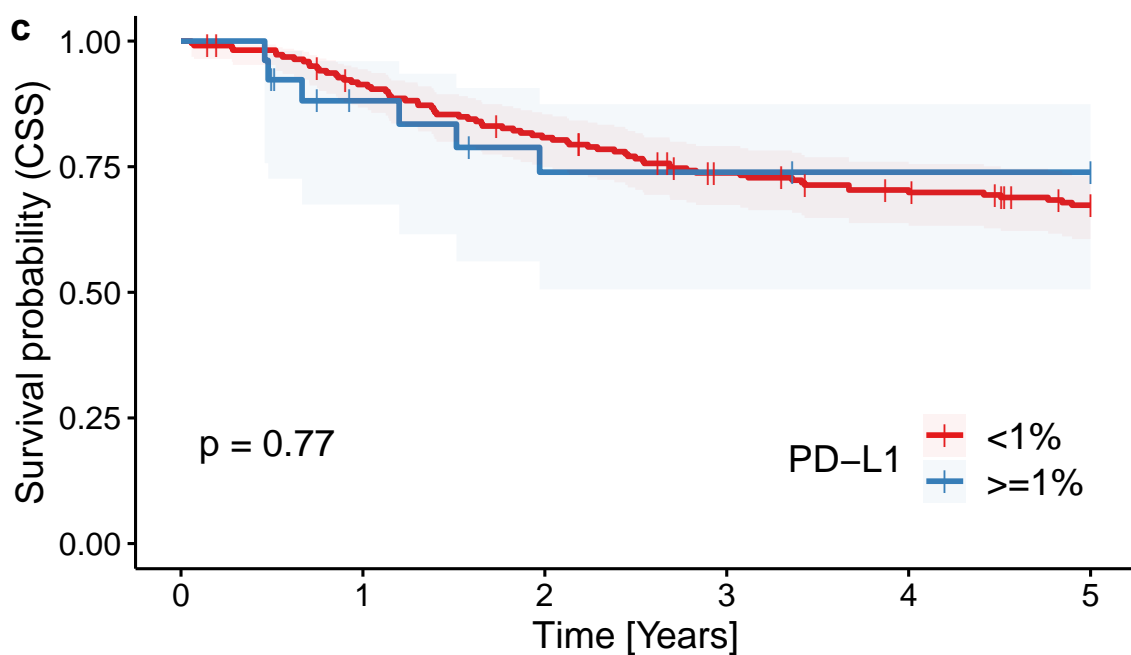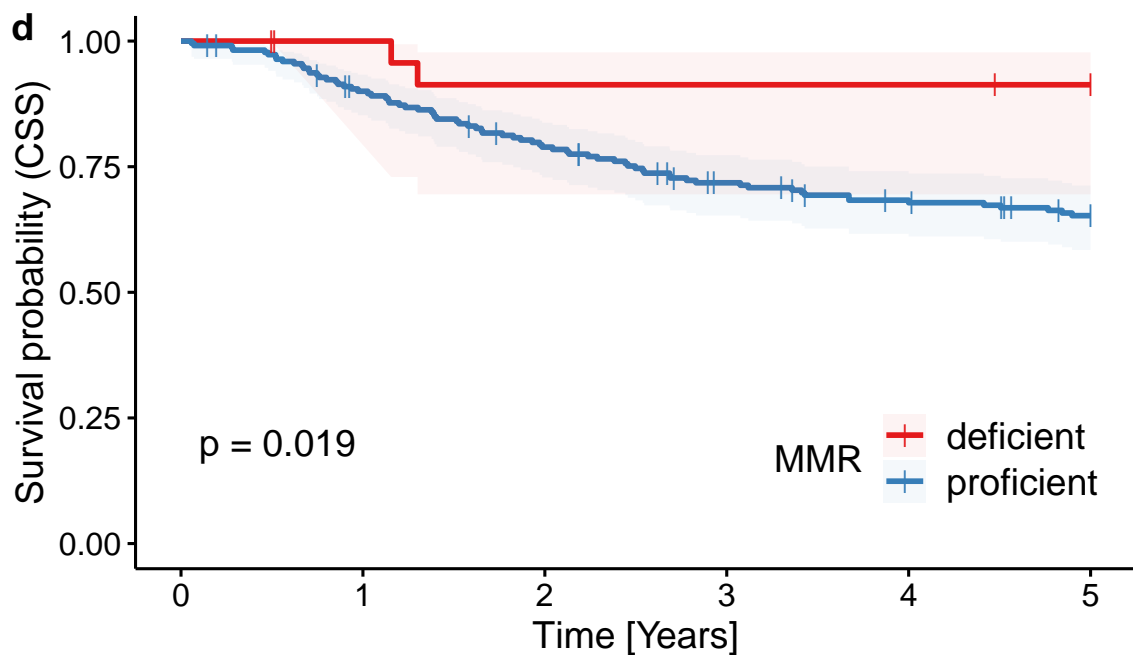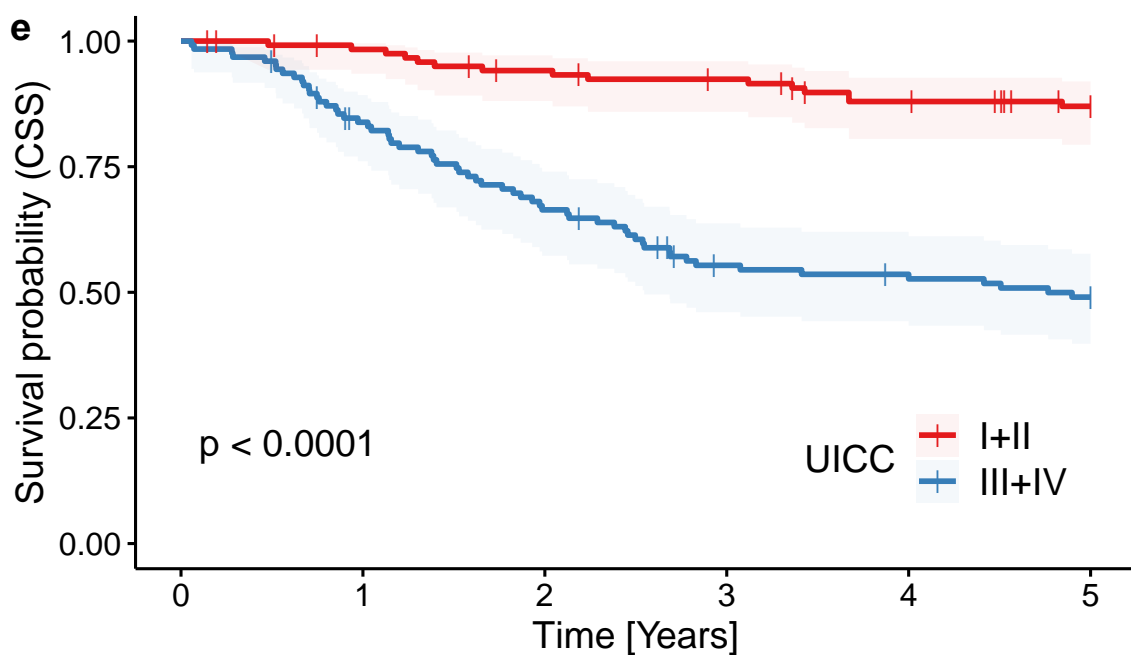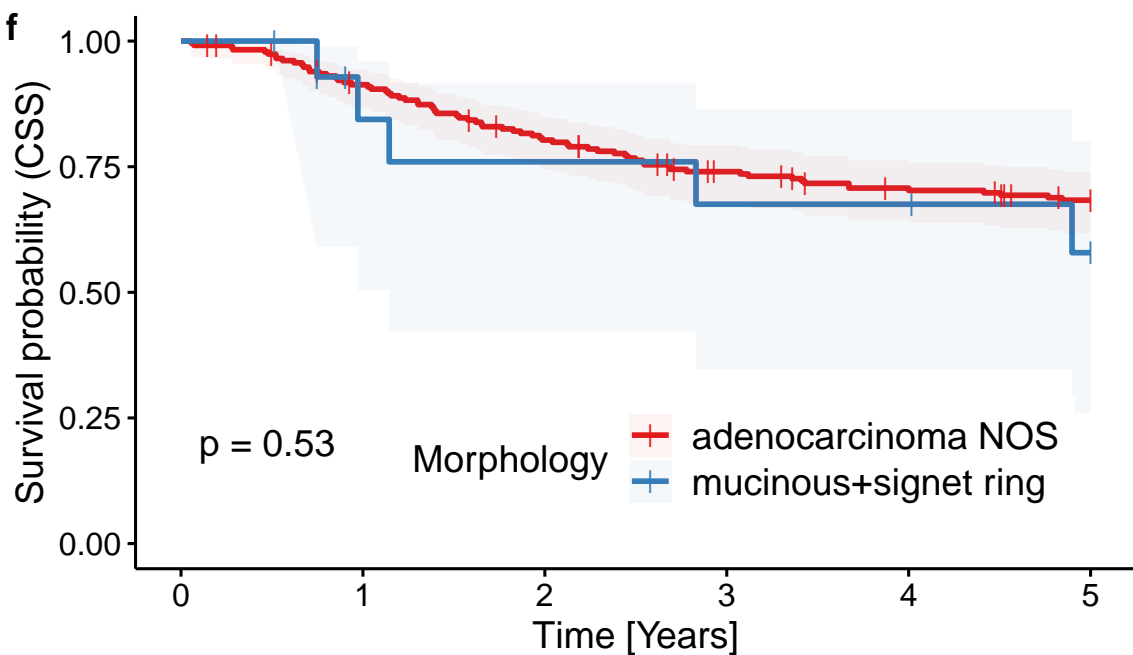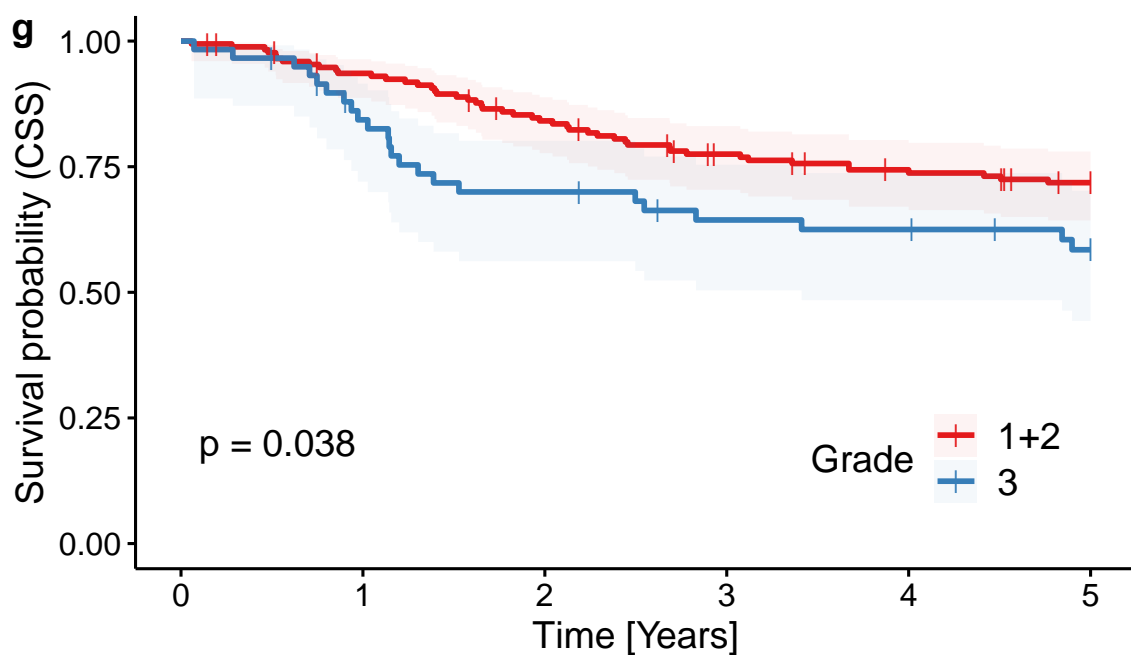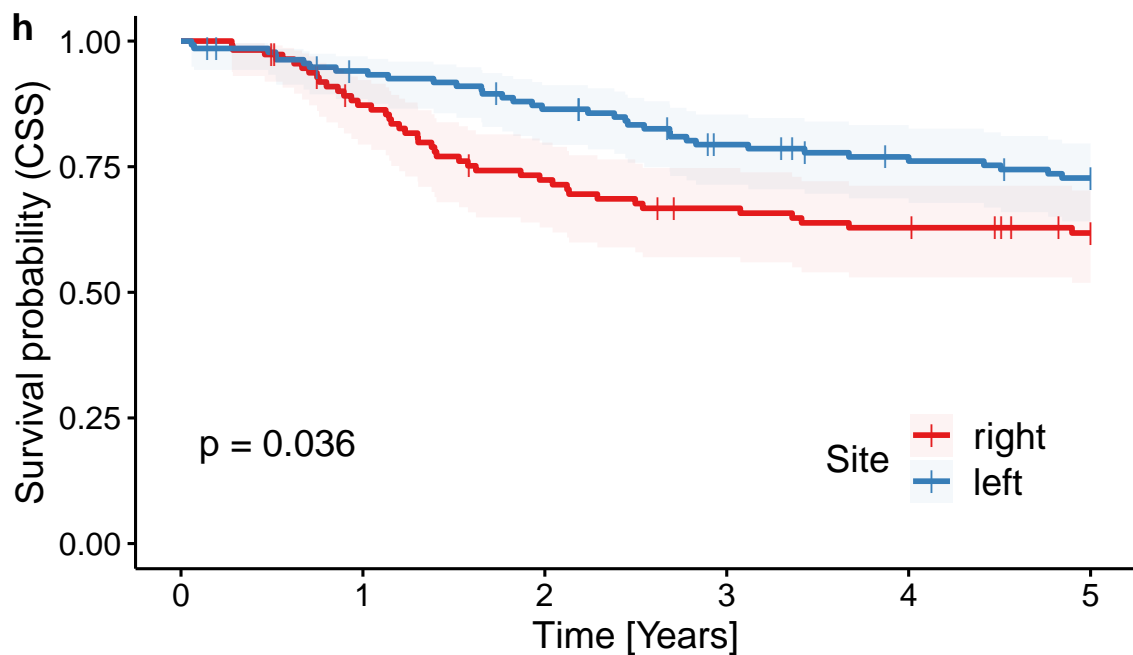

Supplement: Supplementary file 5 — Supplementary Figure 4. [file 41598_2022_22685_MOESM5_ESM.pdf]

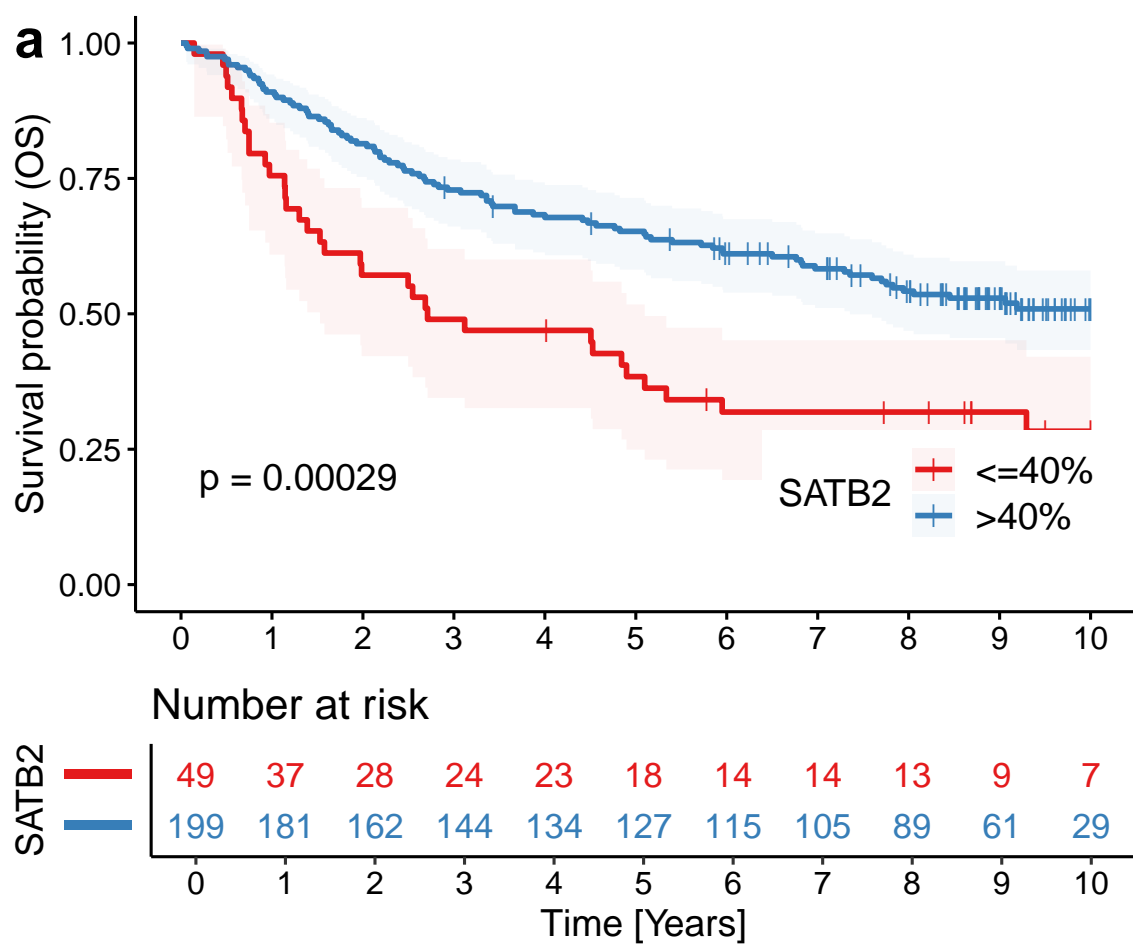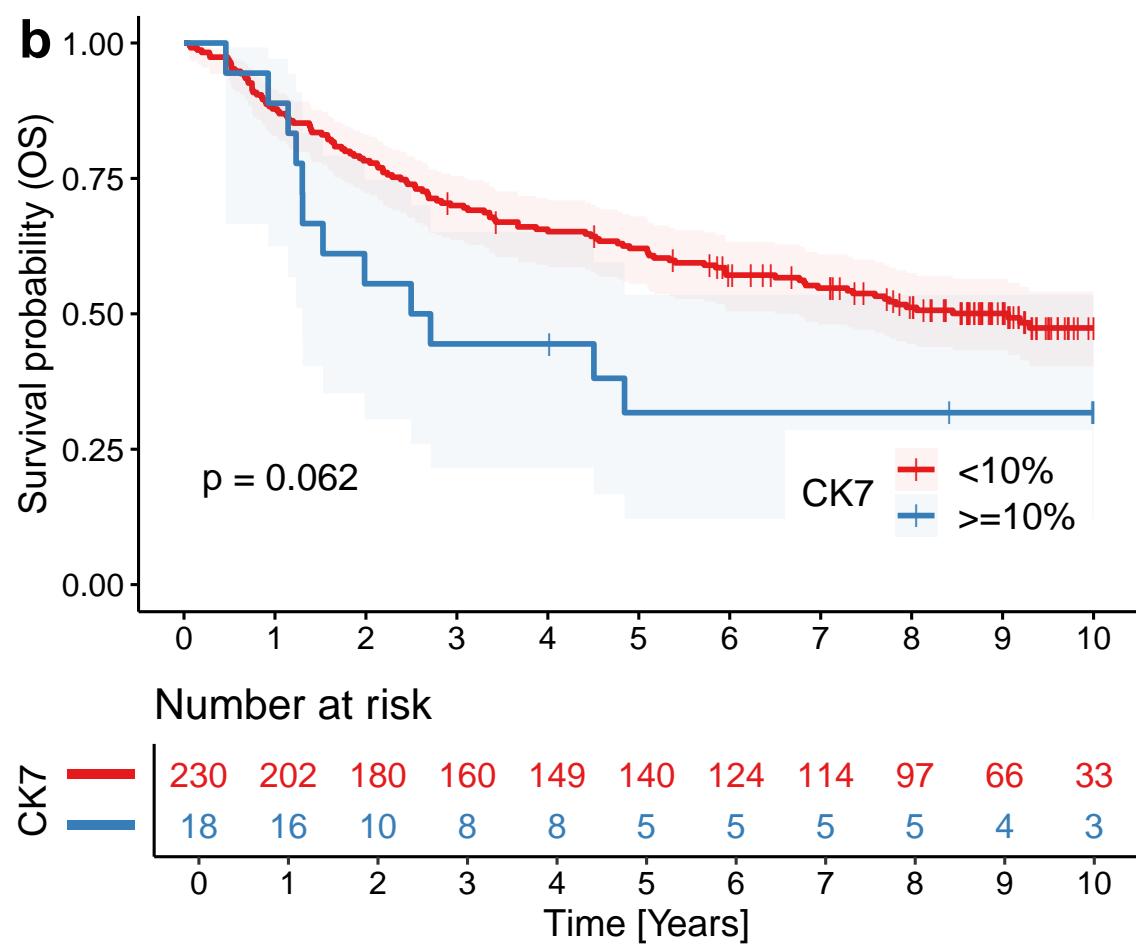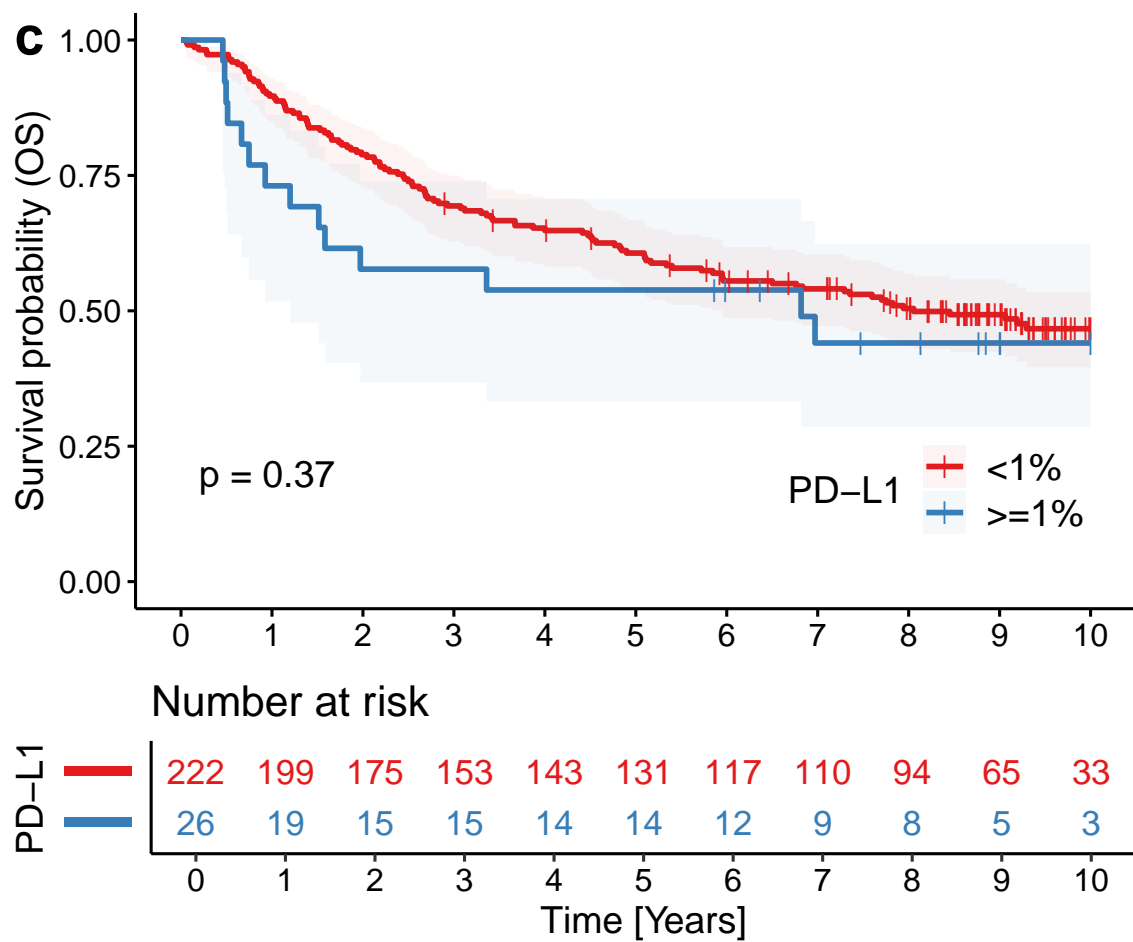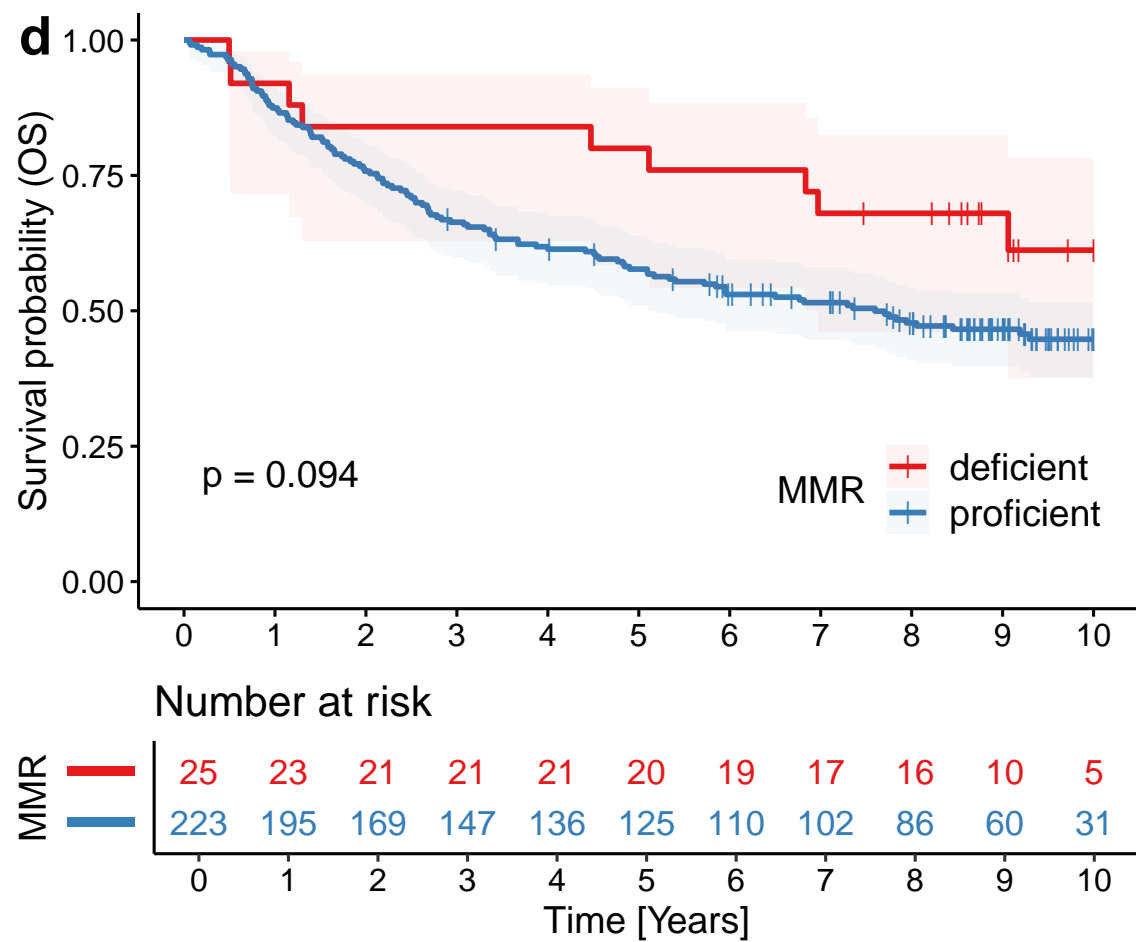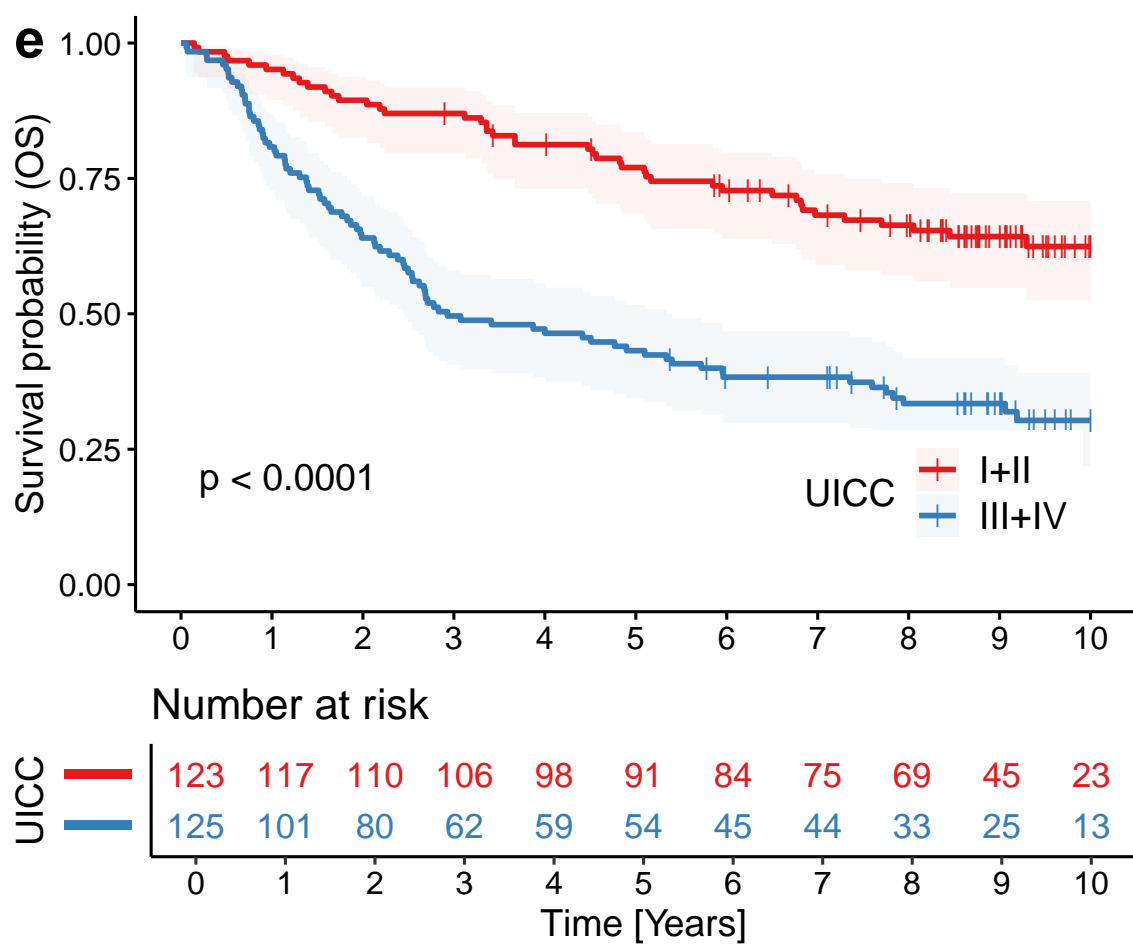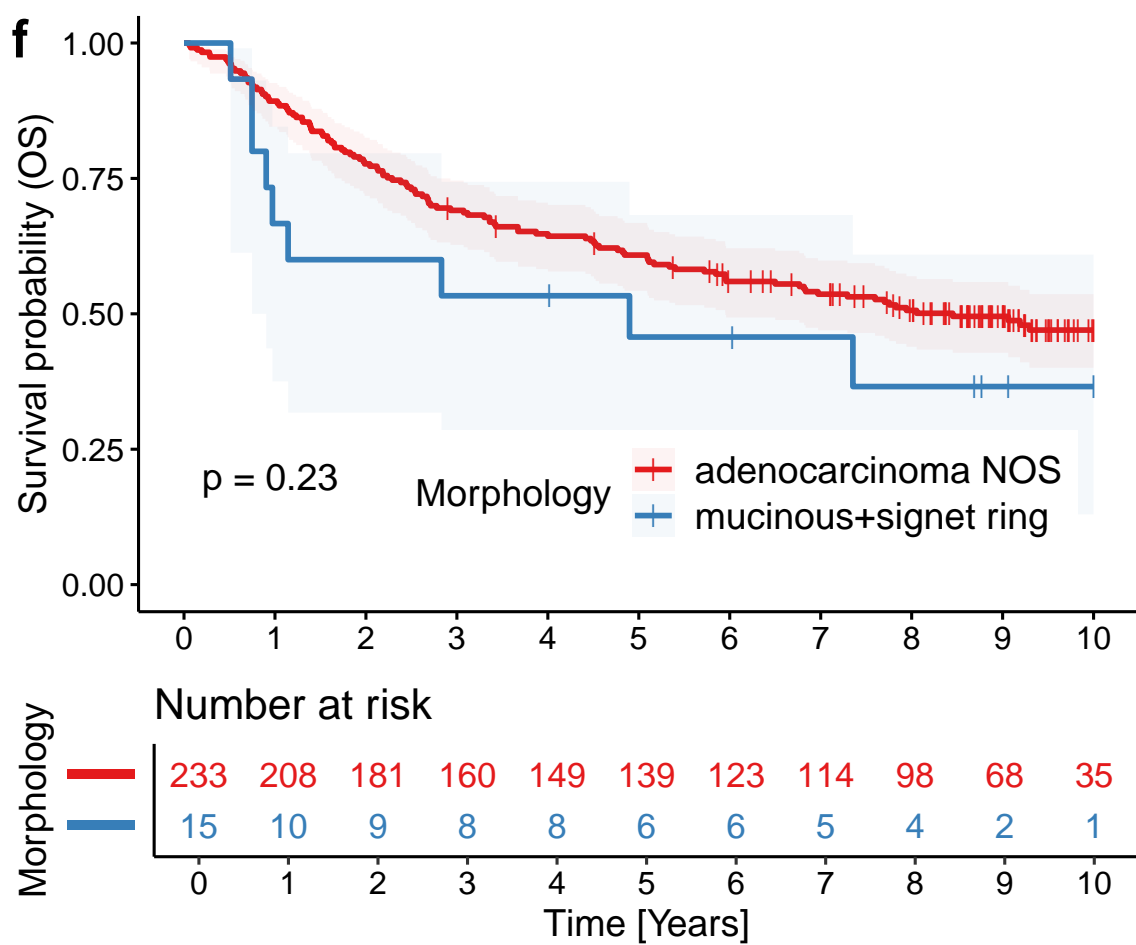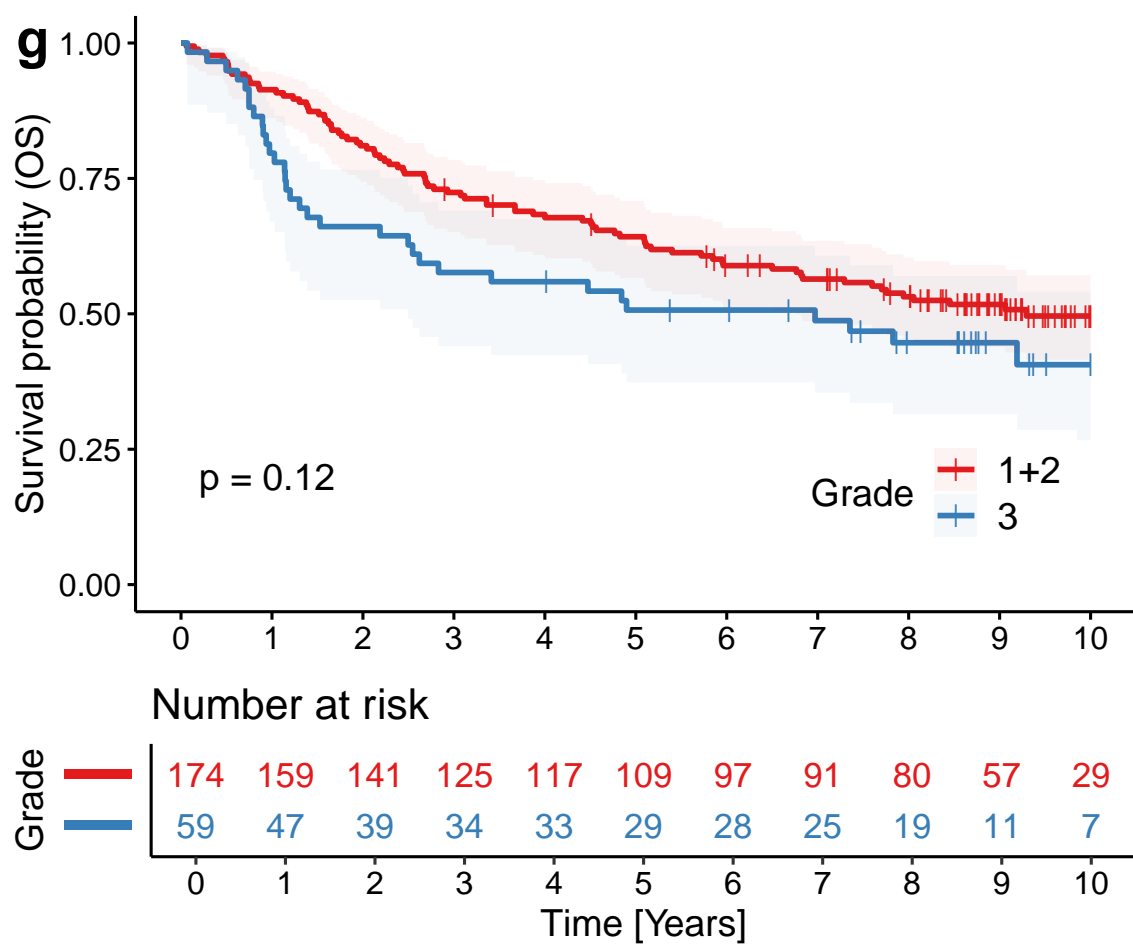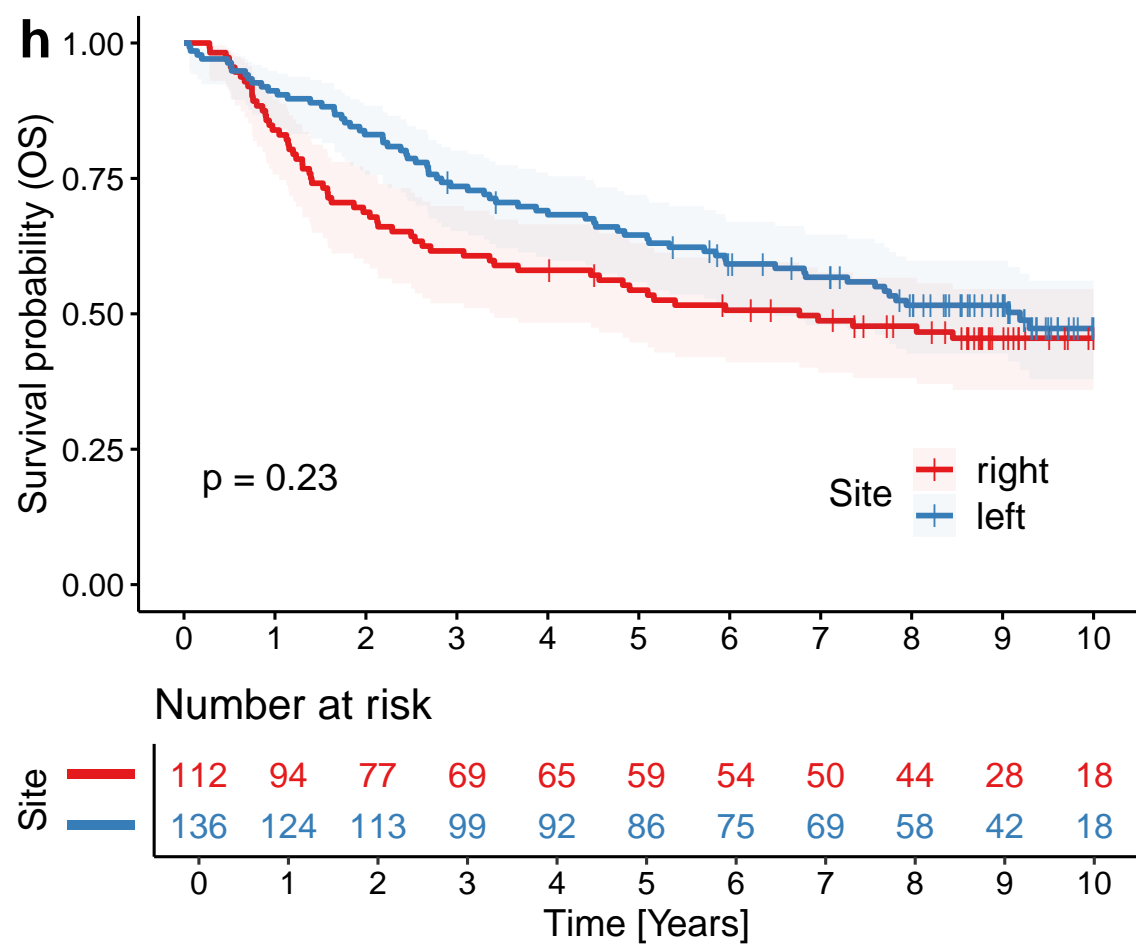

Supplement: Supplementary file 6 — Supplementary Figure 5. [file 41598_2022_22685_MOESM6_ESM.pdf]

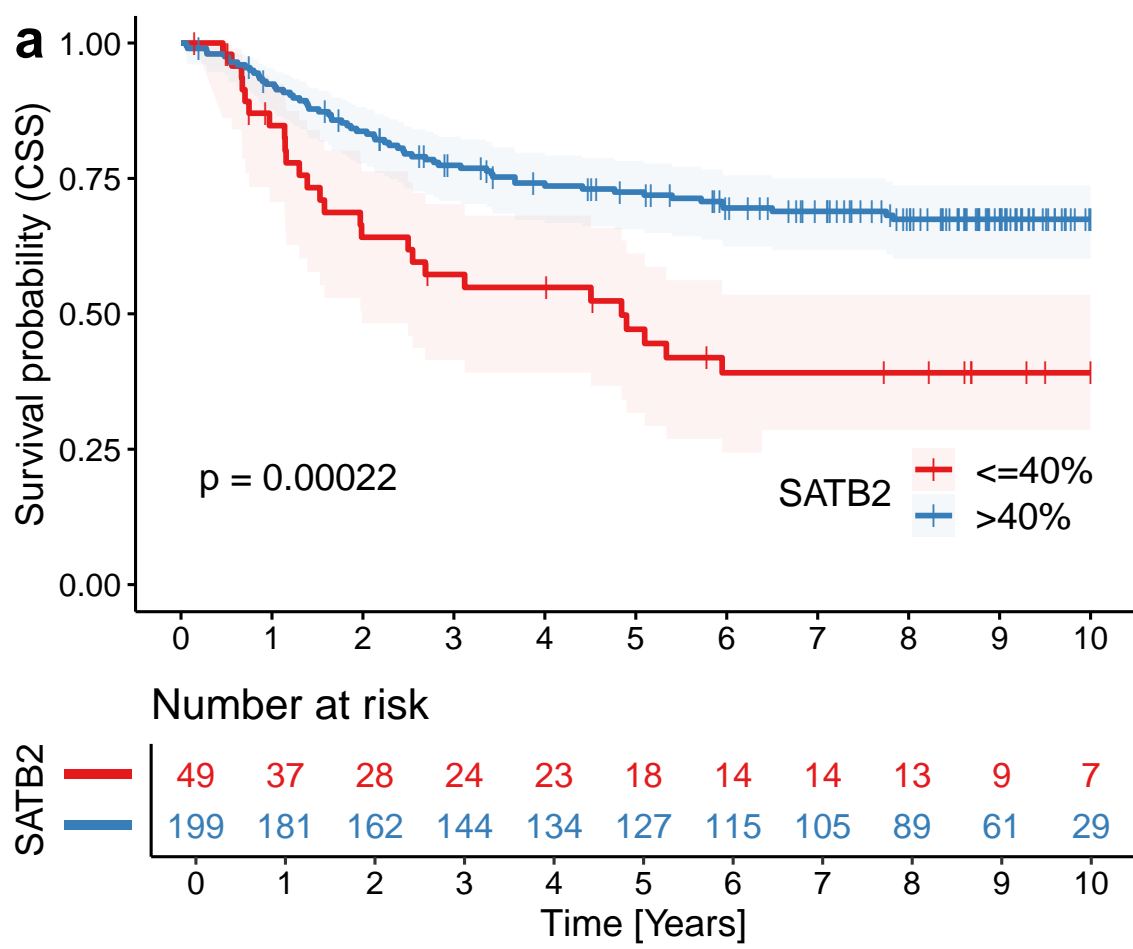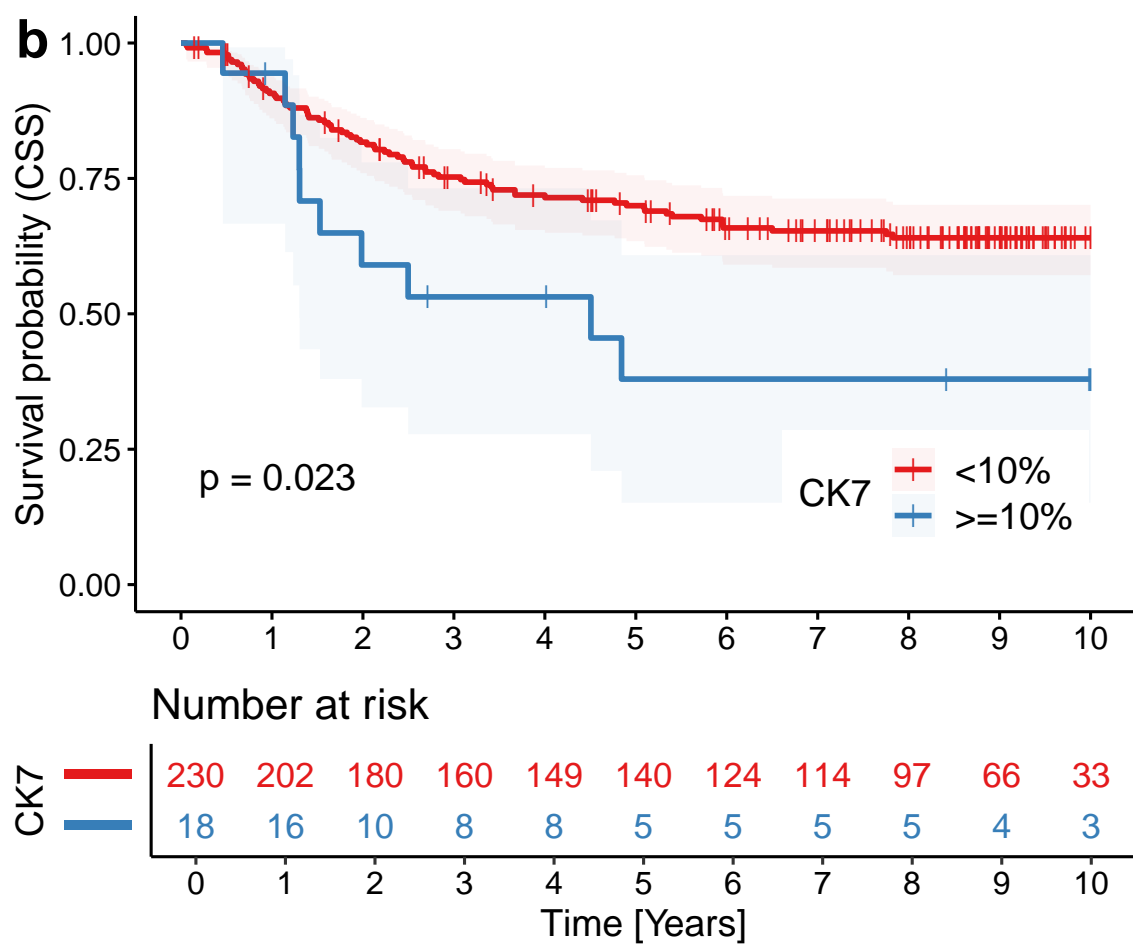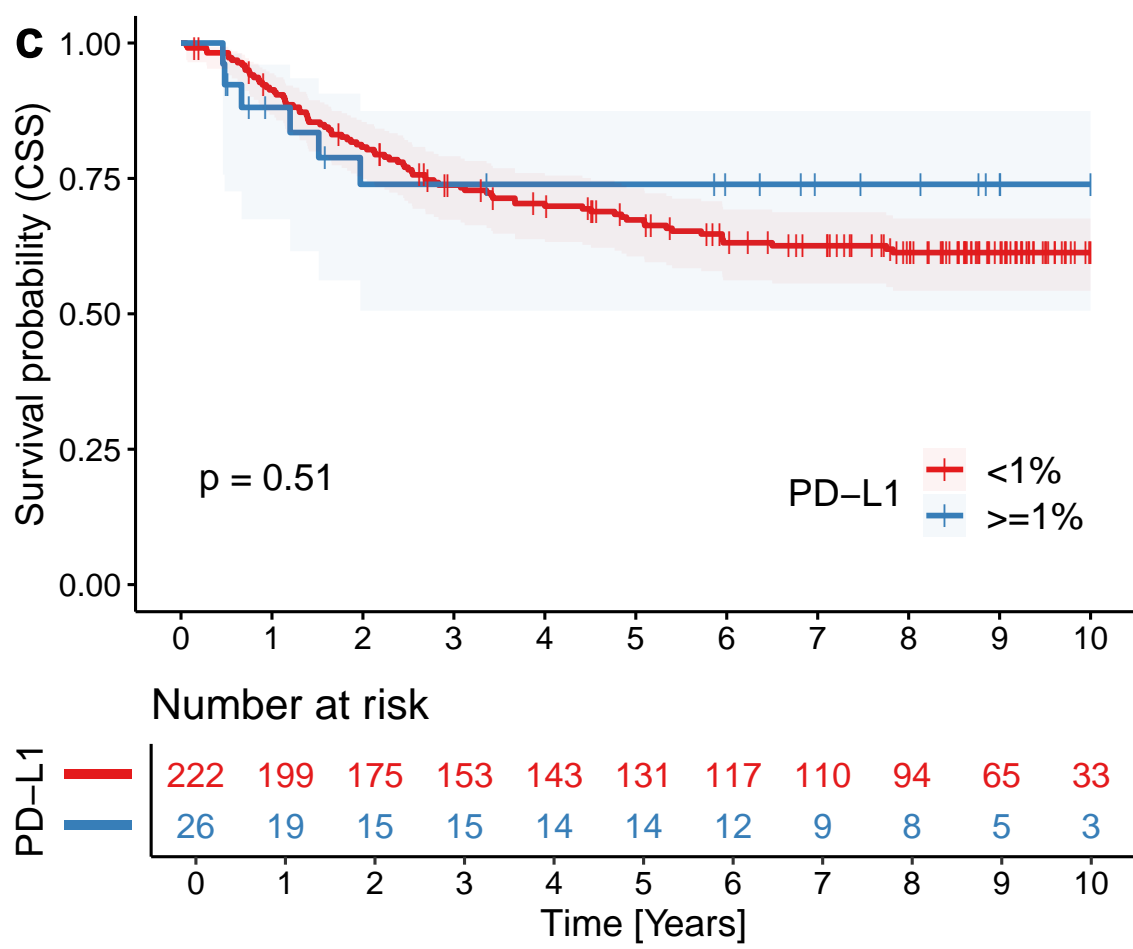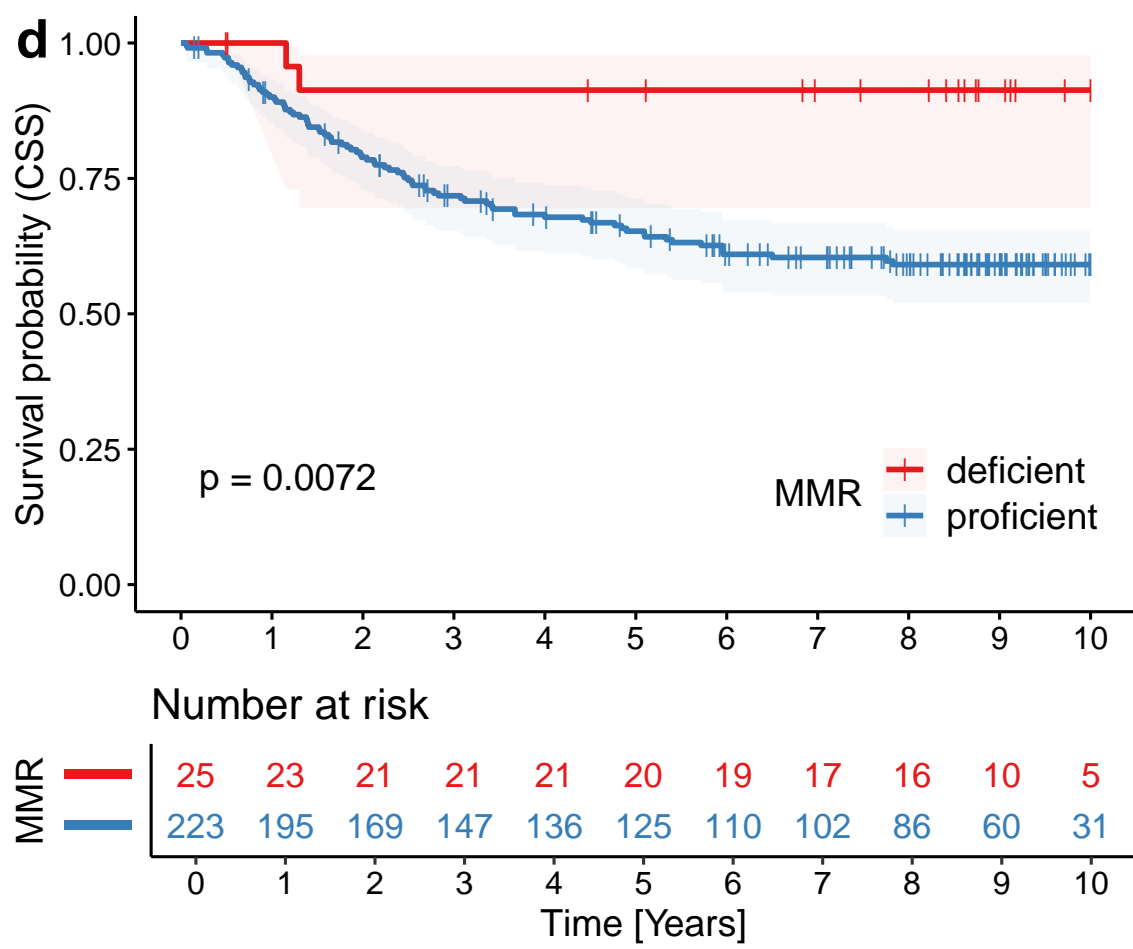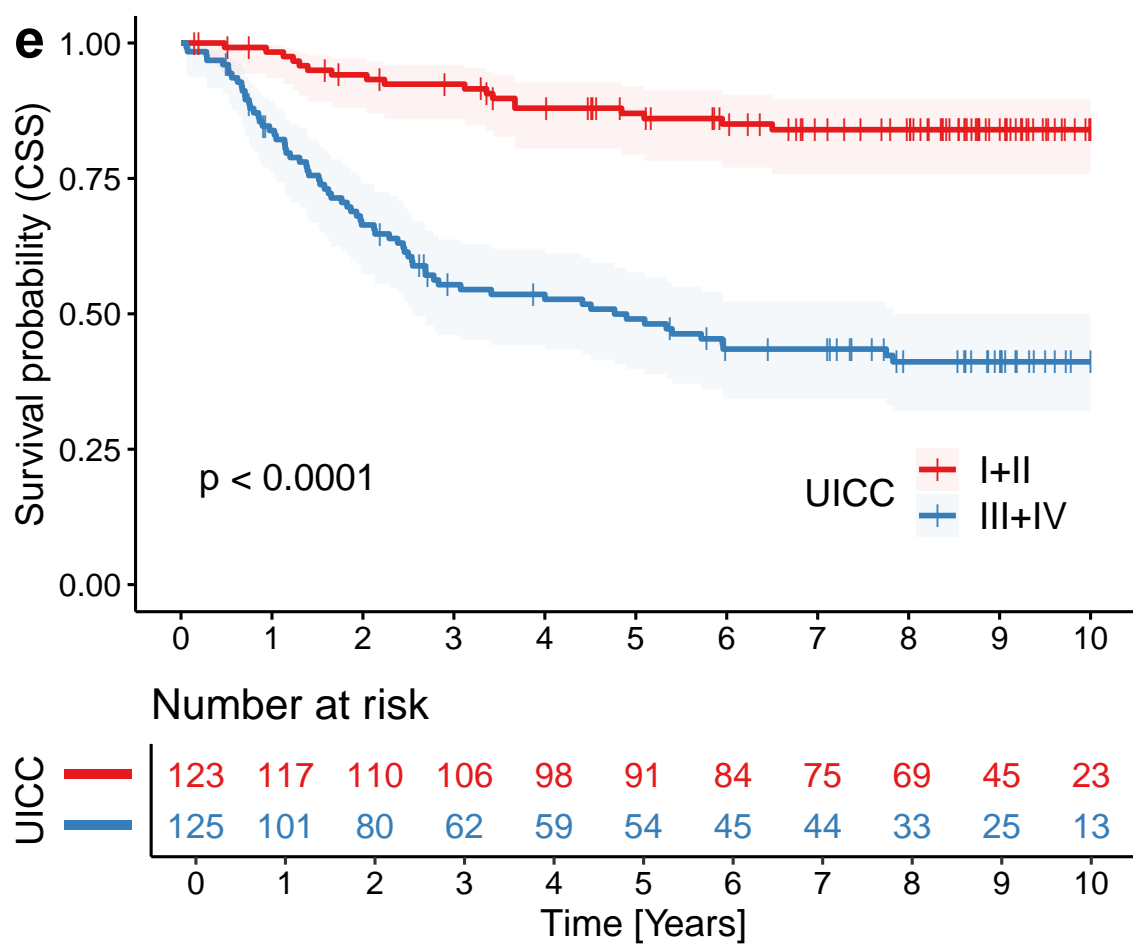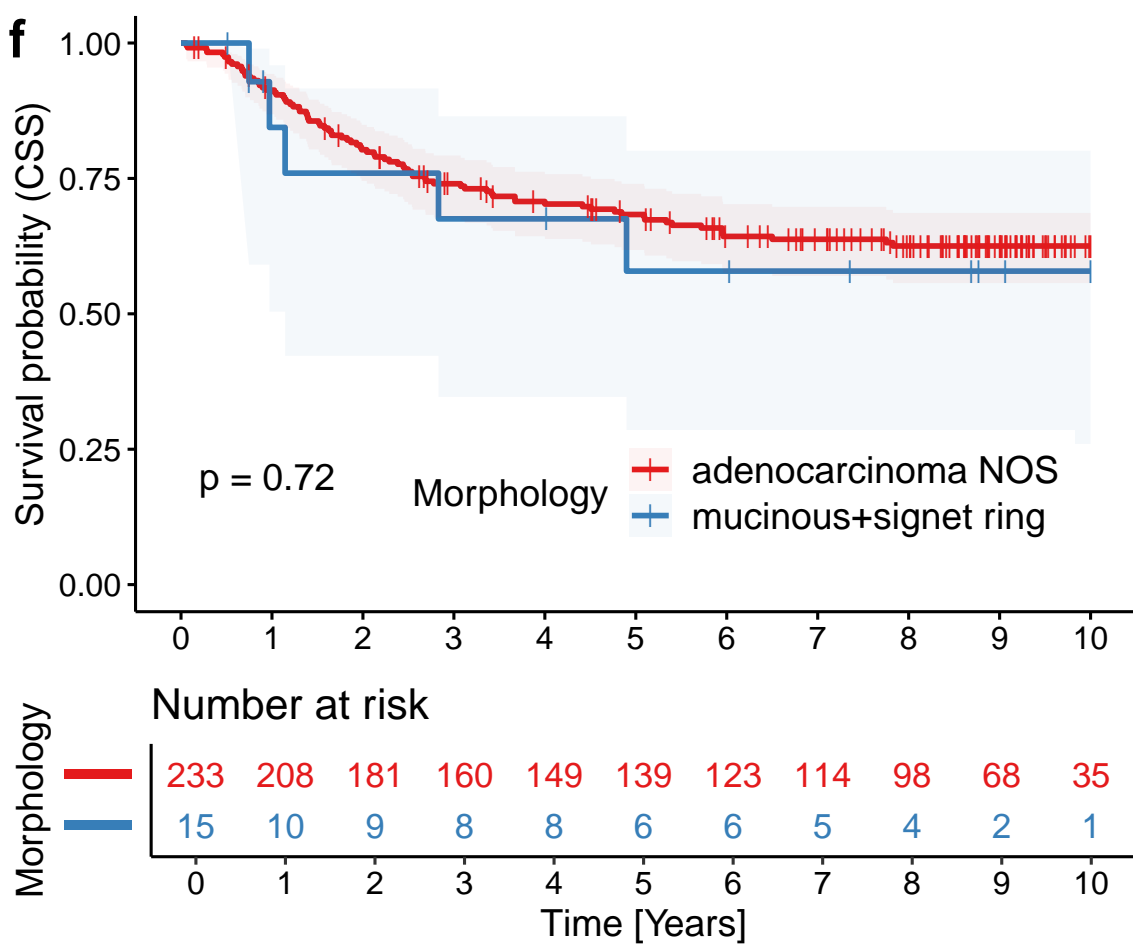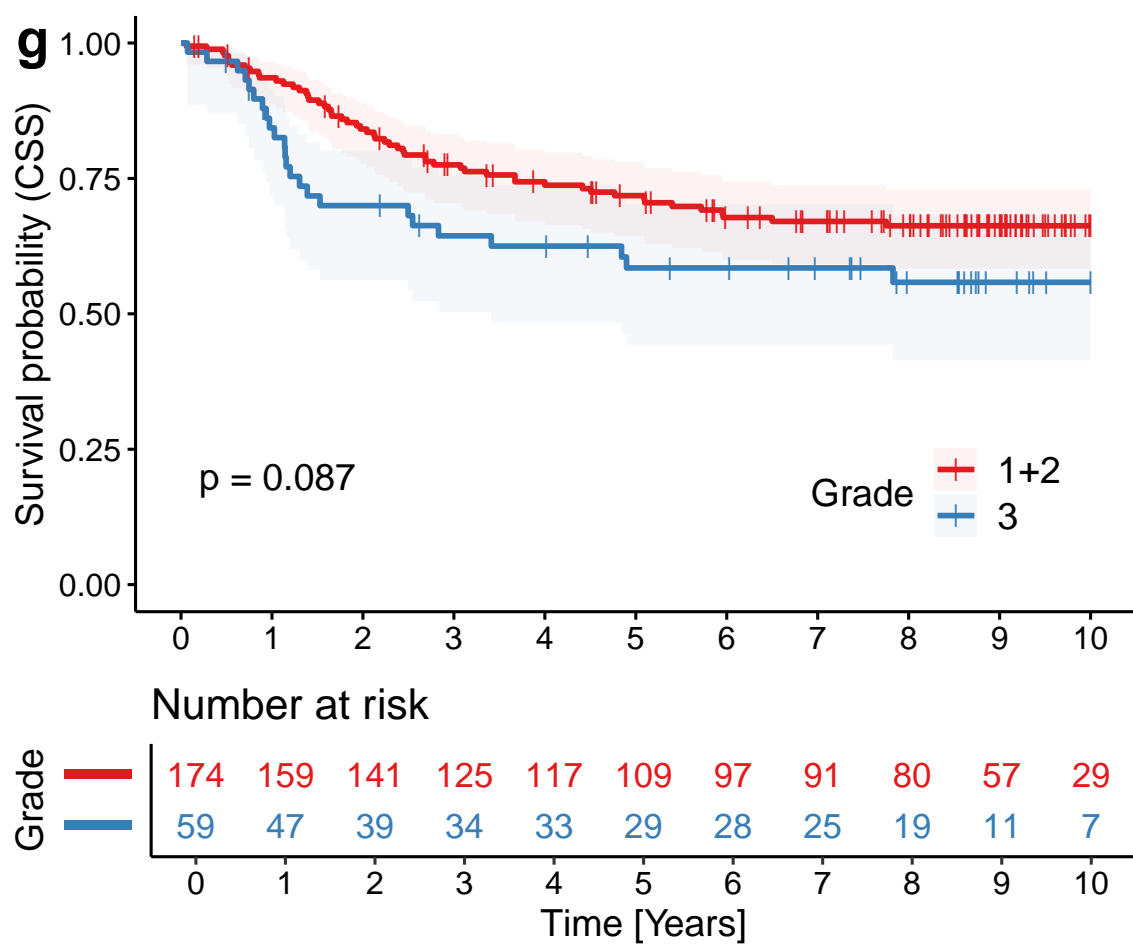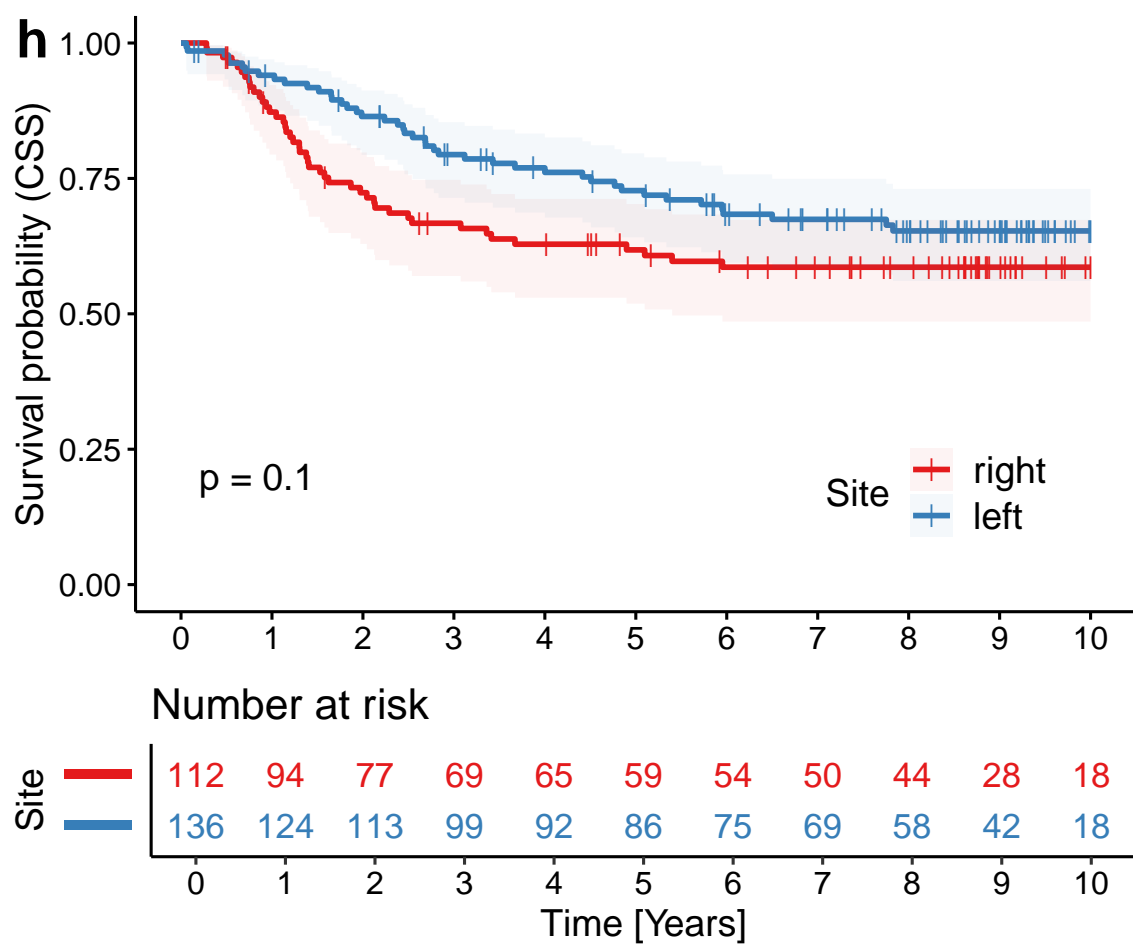

Supplement: Supplementary file 7 — Supplementary Figure 6. [file 41598_2022_22685_MOESM7_ESM.pdf]
