# Supplementary material for: Loss of SATB2 expression correlates with cytokeratin 7 and PD-L1 tumor cell positivity and aggressiveness in colorectal cancer
Source: Sci Rep. 2022 Nov 9;12:19152. doi: 10.1038/s41598-022-22685-0 (PMC9646713; doi:10.1038/s41598-022-22685-0)
Supplement: Supplementary file 9 — Supplementary Table 2. [file 41598_2022_22685_MOESM9_ESM.doc]

Supplementary Table 2 – entire cohort – 5-years follow up - survival analysis - univariate Kaplan-Meier analysis with the log-rank test, restricted mean survival time, Cox regression. Significant p values are in bold.

|  | **n** | **%** | **All deaths** | **Restricted mean OS (years)** | **OS Hazard ratio** | **OS**  **p value (log-rank test)** | **CRC related deaths** | **Restricted mean CSS (years)** | **CSS Hazard ratio** | **CSS p value (log-rank test)** |
| --- | --- | --- | --- | --- | --- | --- | --- | --- | --- | --- |
| SATB2 <= 40% | 54 | 18.9 | 31 | 3.157 | 2.17 | **0.00019** | 24 | 3.493 | 2.16 | **0.0012** |
| SATB2 >40% | 231 | 81.1 | 76 | 4.008 |  |  | 59 | 4.171 |  |  |
| CK7 >=10% | 19 | 6.7 | 12 | 3.124 | 2.13 | **0.012** | 10 | 3.378 | 2.28 | **0.012** |
| CK7 negative | 266 | 93.3 | 95 | 3.898 |  |  | 73 | 4.096 |  |  |
| PD-L1 >= 1% | 28 | 9.8 | 13 | 3.261 | 1.52 | 0.156 | 7 | 3.948 | 1.02 | 0.968 |
| PD-L1 negative | 257 | 90.2 | 94 | 3.910 |  |  | 76 | 4.063 |  |  |
| MMR-deficient | 25 | 8.8 | 5 | 4.317 | 2.16 | 0.085 | 2 | 4.672 | 4.26 | **0.028** |
| MMR-proficient | 260 | 91.2 | 102 | 3.801 |  |  | 81 | 3.992 |  |  |
| UICC I+II | 143 | 50.2 | 33 | 4.383 | 2.88 | **<0.0001** | 19 | 4.624 | 4.29 | **<0.0001** |
| UICC III+IV | 142 | 49.8 | 74 | 3.306 |  |  | 64 | 3.477 |  |  |
| Adenocarcinoma NOS | 269 | 94.4 | 98 | 3.894 | 1.98 | **0.046** | 77 | 4.071 | 1.69 | 0.21 |
| Mucinous+signet ring carcinoma | 16 | 5.6 | 9 | 3.051 |  |  | 6 | 3.620 |  |  |
| Grade 1+2 | 205 | 73.0 | 67 | 4.038 | 1.81 | **0.0031** | 52 | 4.206 | 1.78 | **0.012** |
| Grade 3 | 76 | 27.0 | 38 | 3.348 |  |  | 29 | 3.647 |  |  |
| Right sided CRC | 112 | 39.3 | 51 | 3.502 | 1.62 | **0.013** | 41 | 3.695 | 1.73 | **0.012** |
| Left sided CRC | 173 | 60.7 | 56 | 4.070 |  |  | 42 | 4.278 |  |  |
