# Supplementary material for: Loss of SATB2 expression correlates with cytokeratin 7 and PD-L1 tumor cell positivity and aggressiveness in colorectal cancer
Source: Sci Rep. 2022 Nov 9;12:19152. doi: 10.1038/s41598-022-22685-0 (PMC9646713; doi:10.1038/s41598-022-22685-0)
Supplement: Supplementary file 10 — Supplementary Table 3. [file 41598_2022_22685_MOESM10_ESM.doc]

Supplementary Table 3 – entire cohort – 10-years follow up - survival analysis - univariate Kaplan-Meier analysis with the log-rank test, restricted mean survival time, Cox regression. Significant p values are in bold.

|  | **n** | **%** | **All deaths** | **Restricted mean OS (years)** | **OS Hazard ratio** | **OS**  **p value (log-rank test)** | **CRC related deaths** | **Restricted mean CSS (years)** | **CSS Hazard ratio** | **CSS p value (log-rank test)** |
| --- | --- | --- | --- | --- | --- | --- | --- | --- | --- | --- |
| SATB2 <= 40% | 54 | 18.9 | 36 | 4.921 | 1.96 | **0.00042** | 28 | 5.633 | 2.21 | **0.00027** |
| SATB2 >40% | 231 | 81.1 | 104 | 6.943 |  |  | 70 | 7.648 |  |  |
| CK7 >=10% | 19 | 6.7 | 12 | 5.163 | 1.585 | 0.127 | 10 | 5.468 | 2.0 | **0.035** |
| CK7 negative | 266 | 93.3 | 135 | 6.751 |  |  | 88 | 7.412 |  |  |
| PD-L1 >= 1% | 28 | 9.8 | 16 | 5.527 | 1.46 | 0.15 | 8 | 7.329 | 0.98 | 0.97 |
| PD-L1 negative | 257 | 90.2 | 124 | 6.670 |  |  | 90 | 7.290 |  |  |
| MMR-deficient | 25 | 8.8 | 9 | 7.81 | 1.65 | 0.145 | 2 | 9.237 | 5.29 | **0.0091** |
| MMR-proficient | 260 | 91.2 | 131 | 6.44 |  |  | 96 | 7.107 |  |  |
| UICC I+II | 143 | 50.2 | 51 | 7.769 | 2.37 | **<0.0001** | 24 | 8.757 | 4.08 | **<0.0001** |
| UICC III+IV | 142 | 49.8 | 89 | 5.34 |  |  | 74 | 5.83 |  |  |
| Adenocarcinoma NOS | 269 | 94.4 | 130 | 6.654 | 1.77 | 0.084 | 6 | 6.287 | 1.46 | 0.37 |
| Mucinous+signet ring carcinoma | 16 | 5.6 | 10 | 4.967 |  |  | 92 | 7.338 |  |  |
| Grade 1+2 | 205 | 73.0 | 93 | 6.954 | 1.57 | **0.013** | 63 | 7.649 | 1.65 | **0.021** |
| Grade 3 | 76 | 27.0 | 44 | 5.570 |  |  | 32 | 6.412 |  |  |
| Right sided CRC | 112 | 39.3 | 60 | 5.924 | 1.34 | 0.084 | 44 | 6.641 | 1.45 | 0.064 |
| Left sided CRC | 173 | 60.7 | 80 | 6.973 |  |  | 54 | 7.705 |  |  |
