# Supplementary material for: Loss of SATB2 expression correlates with cytokeratin 7 and PD-L1 tumor cell positivity and aggressiveness in colorectal cancer
Source: Sci Rep. 2022 Nov 9;12:19152. doi: 10.1038/s41598-022-22685-0 (PMC9646713; doi:10.1038/s41598-022-22685-0)
Supplement: Supplementary file 11 — Supplementary Table 4. [file 41598_2022_22685_MOESM11_ESM.doc]

Supplementary Table 4 – neoadjuvant therapy-naïve cohort – 5-years follow up - survival analysis - univariate Kaplan-Meier analysis with the log-rank test, restricted mean survival time, Cox regression. Significant p values are in bold.

|  | **n** | **%** | **All deaths** | **Restricted mean OS (years)** | **OS Hazard ratio** | **OS**  **p value (log-rank test)** | **CRC related deaths** | **Restricted mean CSS (years)** | **CSS Hazard ratio** | **CSS p value (log-rank test)** |
| --- | --- | --- | --- | --- | --- | --- | --- | --- | --- | --- |
| SATB2 <= 40% | 49 | 19.8 | 30 | 3.022 | 2.28 | **0.00011** | 23 | 3.383 | 2.26 | **0.001** |
| SATB2 >40% | 199 | 80.2 | 69 | 3.961 |  |  | 53 | 4.133 |  |  |
| CK7 >=10% | 18 | 7.3 | 12 | 3.019 | 2.16 | **0.01** | 10 | 3.281 | 2.35 | **0.0098** |
| CK7 negative | 230 | 92.73 | 87 | 3.835 |  |  | 66 | 4.048 |  |  |
| PD-L1 >= 1% | 26 | 10.5 | 12 | 3.227 | 1.42 | 0.26 | 6 | 3.982 | 1.13 | 0.77 |
| PD-L1 negative | 222 | 89.5 | 87 | 3.840 |  |  | 70 | 4.0 |  |  |
| MMR-deficient | 23 | 9.3 | 5 | 4.317 | 2.37 | 0.053 | 2 | 4.672 | 4.60 | **0.019** |
| MMR-proficient | 225 | 90.7 | 94 | 3.715 |  |  | 74 | 3.921 |  |  |
| UICC I+II | 123 | 49.6 | 28 | 4.402 | 3.33 | **<0.0001** | 15 | 4.655 | 5.29 | **<0.0001** |
| UICC III+IV | 125 | 50.4 | 71 | 3.160 |  |  | 61 | 3.349 |  |  |
| Adenocarcinoma NOS | 233 | 94.0 | 91 | 3.814 | 1.65 | 0.17 | 71 | 4.002 | 1.33 | 0.53 |
| Mucinous+signet ring carcinoma | 15 | 6.0 | 8 | 3.183 |  |  | 5 | 3.838 |  |  |
| Grade 1+2 | 174 | 74.7 | 62 | 3.952 | 1.61 | **0.033** | 47 | 4.148 | 1.67 | **0.038** |
| Grade 3 | 59 | 25.3 | 29 | 3.362 |  |  | 23 | 3.614 |  |  |
| Right sided CRC | 112 | 45.2 | 51 | 3.502 | 1.46 | 0.057 | 41 | 3.695 | 1.62 | **0.036** |
| Left sided CRC | 136 | 54.8 | 48 | 4.001 |  |  | 35 | 4.240 |  |  |
