# Supplementary material for: Loss of SATB2 expression correlates with cytokeratin 7 and PD-L1 tumor cell positivity and aggressiveness in colorectal cancer
Source: Sci Rep. 2022 Nov 9;12:19152. doi: 10.1038/s41598-022-22685-0 (PMC9646713; doi:10.1038/s41598-022-22685-0)
Supplement: Supplementary file 12 — Supplementary Table 5. [file 41598_2022_22685_MOESM12_ESM.doc]

Supplementary Table 5 – neoadjuvant therapy-naïve cohort – 10-years follow up - survival analysis - univariate Kaplan-Meier analysis with the log-rank test, restricted mean survival time, Cox regression. Significant p values are in bold.

|  | **n** | **%** | **All deaths** | **Restricted mean OS (years)** | **OS Hazard ratio** | **OS**  **p value (log-rank test)** | **CRC related deaths** | **Restricted mean CSS (years)** | **CSS Hazard ratio** | **CSS p value (log-rank test)** |
| --- | --- | --- | --- | --- | --- | --- | --- | --- | --- | --- |
| SATB2 <= 40% | 49 | 19.8 | 34 | 4.621 | 2.04 | **0.00029** | 26 | 5.367 | 2.32 | **0.00022** |
| SATB2 >40% | 199 | 80.2 | 93 | 6.811 |  |  | 61 | 7.574 |  |  |
| CK7 >=10% | 18 | 7.3 | 12 | 4.606 | 1.75 | 0.062 | 10 | 5.178 | 2.12 | **0.023** |
| CK7 negative | 230 | 92.73 | 115 | 6.515 |  |  | 77 | 7.317 |  |  |
| PD-L1 >= 1% | 26 | 10.5 | 14 | 5.616 | 1.29 | 0.37 | 6 | 7.678 | 0.76 | 0.51 |
| PD-L1 negative | 222 | 89.5 | 113 | 6.468 |  |  | 81 | 7.133 |  |  |
| MMR-deficient | 23 | 9.3 | 9 | 7.81 | 1.77 | 0.094 | 2 | 9.237 | 5.52 | **0.0072** |
| MMR-proficient | 225 | 90.7 | 118 | 6.219 |  |  | 85 | 6.945 |  |  |
| UICC I+II | 123 | 49.6 | 43 | 7.816 | 2.76 | **<0.0001** | 18 | 8.881 | 5.20 | **<0.0001** |
| UICC III+IV | 125 | 50.4 | 84 | 4.964 |  |  | 69 | 5.503 |  |  |
| Adenocarcinoma NOS | 233 | 94.0 | 118 | 6.453 | 1.51 | 0.23 | 5 | 6.732 | 1.18 | 0.72 |
| Mucinous+signet ring carcinoma | 15 | 6.0 | 9 | 5.227 |  |  | 82 | 7.194 |  |  |
| Grade 1+2 | 174 | 74.7 | 84 | 6.723 | 1.38 | 0.12 | 55 | 7.515 | 1.52 | 0.087 |
| Grade 3 | 59 | 25.3 | 33 | 5.707 |  |  | 24 | 6.480 |  |  |
| Right sided CRC | 112 | 45.2 | 60 | 5.924 | 1.24 | 0.23 | 44 | 6.641 | 1.42 | 0.1 |
| Left sided CRC | 136 | 54.8 | 67 | 6.757 |  |  | 43 | 7.606 |  |  |
