# Supplementary material for: Loss of SATB2 expression correlates with cytokeratin 7 and PD-L1 tumor cell positivity and aggressiveness in colorectal cancer
Source: Sci Rep. 2022 Nov 9;12:19152. doi: 10.1038/s41598-022-22685-0 (PMC9646713; doi:10.1038/s41598-022-22685-0)
Supplement: Supplementary file 13 — Supplementary Table 6. [file 41598_2022_22685_MOESM13_ESM.doc]

Supplementary Table 6 – entire cohort – unweighted Cohen’s kappa test describing intercore reliability among two tumor samples in all three examined markers (SATB2, CK7, PD-L1) and interobserver reliability among pathologist 1 (JH) and pathologist 2 (RM).

|  | Cohen’s kappa among core 1 and 2 | Cohen’s kappa among consensus value and core 1 | Cohen’s kappa among consensus value and core 2 | Cohen’s kappa interobserver  Core 1 | Cohen’s kappa interobserver  Core 2 |
| --- | --- | --- | --- | --- | --- |
| **CK7** | 0.913 | 0.913 | 1 | NA | NA |
| **SATB2** | 0.85 | 0.931 | 0.919 | NA | NA |
| **PD-L1** | 0.518 | 0.684 | 0.662 | 0.795 | 0.884 |
