# Supplementary material for: Loss of SATB2 expression correlates with cytokeratin 7 and PD-L1 tumor cell positivity and aggressiveness in colorectal cancer
Source: Sci Rep. 2022 Nov 9;12:19152. doi: 10.1038/s41598-022-22685-0 (PMC9646713; doi:10.1038/s41598-022-22685-0)
Supplement: Supplementary file 14 — Supplementary Table 7. [file 41598_2022_22685_MOESM14_ESM.doc]

Supplementary Table 7 – entire cohort – overall survival and cancer specific survival analysis according to percentage of PD-L1 expression - univariate Kaplan-Meier analysis with the log-rank test, restricted mean survival time, Cox regression. Significant p-value in bold.

| **5- year follow up** | | | | | | | | | | | |
| --- | --- | --- | --- | --- | --- | --- | --- | --- | --- | --- | --- |
|  | **n** | **%** | **All deaths** | **Restricted mean OS (years)** | **OS Hazard ratio** | **OS**  **p value (log-rank test)** | **CRC related deaths** | **Restricted mean CSS (years)** | | **CSS Hazard ratio** | **CSS p value (log-rank test)** |
| PD-L1 50-100% | 4 | 1.4% | 3 | 3.910 | 2.42 | 0.062 | 1 | 3.838 | | 1.28 | 0.94 |
| PD-L1 1-49% | 24 | 8.4% | 10 | 3.446 | 1.34 |  | 6 | 3.976 | | 1.18 |  |
| PD-L1 <1% | 257 | 90.2% | 94 | 2.148 | 1 |  | 76 | 4.063 | | 1 |  |
| **10- year follow/up** | | | | | | | | | | | |
| PD-L1 50-100% | 4 | 1.4% | 4 | 2.641 | 2.71 | **0.01** | 1 | 7.171 | 1.19 | | 0.96 |
| PD-L1 2-49% | 24 | 8.4% | 12 | 6.033 | 1.53 |  | 7 | 7.392 | 1.15 | |  |
| PD-L1 <= 1% | 257 | 90.2% | 124 | 6.670 | 1 |  | 90 | 7.290 | 1 | |  |
