# Supplementary material for: Loss of SATB2 expression correlates with cytokeratin 7 and PD-L1 tumor cell positivity and aggressiveness in colorectal cancer
Source: Sci Rep. 2022 Nov 9;12:19152. doi: 10.1038/s41598-022-22685-0 (PMC9646713; doi:10.1038/s41598-022-22685-0)
Supplement: Supplementary file 15 — Supplementary Table 8. [file 41598_2022_22685_MOESM15_ESM.doc]

Supplementary Table 8 – neoadjuvant-therapy naïve cohort – overall survival and cancer specific survival analysis according to percentage of PD-L1 expression - univariate Kaplan-Meier analysis with the log-rank test, restricted mean survival time, Cox regression. Significant p-value in bold.

| **5 -year follow up** | | | | | | | | | | |
| --- | --- | --- | --- | --- | --- | --- | --- | --- | --- | --- |
|  | **n** | **%** | **All deaths** | **Restricted mean OS (years)** | **OS Hazard ratio** | **OS**  **p value (log-rank test)** | **CRC related deaths** | **Restricted mean OS (years)** | **CSS Hazard ratio** | **CSS p value (log-rank test)** |
| PD-L1 50-100% | 4 | 1.6% | 3 | 2.148 | 2.30 | 0.099 | 1 | 3.838 | 1.30 | 0.89 |
| PD-L1 1-49% | 22 | 8.9% | 9 | 3.424 | 1.40 |  | 5 | 4.013 | 1.21 |  |
| PD-L1 < 1% | 222 | 89.5% | 87 | 3.840 | 1 |  | 70 | 4.0 | 1 |  |
| **10- year follow/up** | | | | | | | | | | |
| PD-L1 50-100% | 4 | 1.6% | 4 | 6.468 | 2.58 | **0.019** | 1 | 7.171 | 1.43 | 0.74 |
| PD-L1 1-49% | 22 | 8.9% | 10 | 6.190 | 1.70 |  | 5 | 7.781 | 1.14 |  |
| PD-L1 < 1% | 222 | 89.5% | 113 | 2.641 | 1 |  | 81 | 7.133 | 1 |  |
