# Supplementary material for: Loss of SATB2 expression correlates with cytokeratin 7 and PD-L1 tumor cell positivity and aggressiveness in colorectal cancer
Source: Sci Rep. 2022 Nov 9;12:19152. doi: 10.1038/s41598-022-22685-0 (PMC9646713; doi:10.1038/s41598-022-22685-0)
Supplement: Supplementary file 16 — Supplementary Table 9. [file 41598_2022_22685_MOESM16_ESM.doc]

Supplementary Table 9 – Results of logistic regression in the neoadjuvant therapy-naïve cohort; significant and borderline insignificant associations are in bold, the main findings of the study are in bold and underlined – association of CK7 expression and SATB2 loss.

|  | SATB2<=40% (n=49) | SATB2 >40% (n=199) | CK7 >=10% (n=19) | CK7 negative (n=266) | PD-L1>= 1% (n=19) | PD-L1 negative (n=266) |
| --- | --- | --- | --- | --- | --- | --- |
| SATB2<=40% (n=49) |  |  | 13 (26.5%) | 36(73.5%) | 7 (14.3%) | 42 (85.7%) |
|  |  |  |  |  |  |  |
| SATB2 >40% (n=199) |  |  | 5 (2.5%) | 194 (97.5%) | 19 (9.5%) | 180 (90.5%) |
|  |  |  | OR=0.071 | **p<0.001** | OR=0.633 | p=0.335 |
| CK7 >=10% (n=18) | 13 (72.2%) | 5 (27.8%) |  |  | 2 (11.1%) | 16 (88.9%) |
|  |  |  |  |  |  |  |
| CK7 negative (n=230) | 36 (15.7%) | 194 (84.3%) |  |  | 24 (10.4%) | 206 (89.6%) |
|  | OR=0.071 | **p<0.001** |  |  | OR=1.073 | p=0.928 |
| PD-L1>= 1%(n=26) | 7 (26.9%) | 19 (73.1%) | 2 (7.7%) | 24 (92.3%) |  |  |
|  |  |  |  |  |  |  |
| PD-L1 negative (n=222) | 42 (18.9%) | 180 (81.1%) | 16 (7.2%) | 206 (92.8%) |  |  |
|  | OR=0.653 | p=0.335 | OR=1.073 | p=0.928 |  |  |
| MMR-proficient (n=223) | 42 (18.8%) | 181(81.2%) | 16 (7.2%) | 207 (92.8%) | 20 (9.0%) | 203 (91.0%) |
|  |  |  |  |  |  |  |
| MMR-deficient (n=25) | 7 (28.0%) | 18 (72.0%) | 2 (8.0%) | 23 (92.0%) | 6 (24.0%) | 19 (76.0%) |
|  | OR=1.676 | p=0.279 | OR=0.889 | p=0.88 | OR=0.312 | **p=0.026** |
| UICC I+II (n=123) | 17 (13.8%) | 106 (86.2%) | 9 (7.3%) | 114 (92.7%) | 16 (13.0%) | 107 (87.0%) |
|  |  |  |  |  |  |  |
| UICC III+IV (n=125) | 32 (25.6%) | 93 (74.4%) | 9 (7.2%) | 116 (92.8%) | 10 (8.0%) | 115 (92.0%) |
|  | OR=0.466 | **p=0.022** | OR=0.983 | p=0.912 | OR=0.582 | p=0.202 |
| Adenocarcinoma NOS (233) | 42 (18.0%) | 191 (82.0%) | 16 (6.9%) | 217 (93.1%) | 24 (10.3%) | 209 (89.7%) |
|  |  |  |  |  |  |  |
| Mucinous+signet ring carcinoma (n=15) | 7 (46.7%) | 8 (53.3%) | 2 (13.3%) | 13 (86.7%) | 2 (12.5%) | 13 (87.5%) |
|  | OR=5.979 | **p=0.011** | OR=0.479 | p=0.359 | OR=0.746 | p=0.711 |
| Grade 1+2 (n=174) | 29 (16.7%) | 145 (83.3%) | 9 (5.2%) | 165 (94.8%) | 16 (9.2%) | 158 (90.8%) |
|  |  |  |  |  |  |  |
| Grade 3 (n=59) | 15 (25.4%) | 44 (74.6%) | 7 (11.9%) | 52 (88.1%) | 7 (13.2%) | 52 (86.8%) |
|  | OR=0.587 | p=0.14 | OR=2.486 | **p=0.087** | OR=1.329 | p=0.554 |
| Right sided CRC (n=112) | 29 (25.9%) | 83 (74.1%) | 11 (9.8%) | 101 (90.2%) | 13 (11.6%) | 99 (88.4%) |
|  |  |  |  |  |  |  |
| Left sided CRC (n=136) | 20 (14.5%) | 116 (85.5%) | 7 (5.1%) | 129 (94.9%) | 13 (9.6%) | 123 (88.4%) |
|  | OR=2.027 | **p=0.029** | OR=0.498 | p=0.165 | OR=0.805 | p=0.601 |
